# Media use and vaccine resistance

**DOI:** 10.1093/pnasnexus/pgad146

**Published:** 2023-05-09

**Authors:** Jon Green, James N Druckman, Matthew A Baum, Katherine Ognyanova, Matthew D Simonson, Roy H Perlis, David Lazer

**Affiliations:** Network Science Institute, Northeastern University, Boston, MA 02148, United States; Shorenstein Center on Media, Politics and Public Policy, Harvard Kennedy School, Cambridge, MA 02138, United States; Department of Political Science, Northwestern University, Evanston, IL 60208, United States; Shorenstein Center on Media, Politics and Public Policy, Harvard Kennedy School, Cambridge, MA 02138, United States; School of Communication and Information, Rutgers University, Piscataway, NJ 08854, United States; Department of Political Science, University of Pennsylvania, Philadelphia, PA 19104, United States; Department of Psychiatry, Harvard Medical School, Boston, MA 02114, United States; Network Science Institute, Northeastern University, Boston, MA 02148, United States

## Abstract

Public health requires collective action—the public best addresses health crises when individuals engage in prosocial behaviors. Failure to do so can have dire societal and economic consequences. This was made clear by the disjointed, politicized response to COVID-19 in the United States. Perhaps no aspect of the pandemic exemplified this challenge more than the sizeable percentage of individuals who delayed or refused vaccination. While scholars, practitioners, and the government devised a range of communication strategies to persuade people to get vaccinated, much less attention has been paid to where the unvaccinated could be reached. We address this question using multiple waves of a large national survey as well as various secondary data sets. We find that the vaccine resistant seems to predictably obtain information from conservative media outlets (e.g. Fox News) while the vaccinated congregate around more liberal outlets (e.g. MSNBC). We also find consistent evidence that vaccine-resistant individuals often obtain COVID-19 information from various social media, most notably Facebook, rather than traditional media sources. Importantly, such individuals tend to exhibit low institutional trust. While our results do not suggest a failure of sites such as Facebook's institutional COVID-19 efforts, as the counterfactual of no efforts is unknown, they do highlight an opportunity to reach those who are less likely to take vital actions in the service of public health.

**Significance Statement**

Public health campaigns often seek to persuade citizens to engage in particular behaviors—such as getting vaccinated. While considerable work identifies what messages are most or least effective, much less is known about where within the information environment noncompliant individuals reside. We address this issue by exploring where COVID-19 vaccine-hesitant individuals congregate. Original and secondary survey data show that the unvaccinated are much less likely to access institutional, mainstream, or liberal sources. They are more likely attended to conservative outlets (e.g. Fox and Newsmax) and some social media platforms (e.g. Facebook). They also exhibit relatively low levels of trust in mainstream institutions. This highlights the importance of interventions on large social media platforms, such as Facebook, where incidental exposure to such information is likely.

## Introduction

The onset of COVID-19 led much of the world to turn its attention to the medical community for guidance. Within a year, scientists created and began to distribute a vaccine. Yet, an equally daunting challenge involved persuading people to get vaccinated against the disease. While social scientists have evaluated a range of interventions aimed at persuading the unvaccinated (1–3), they have provided scant insight into *where* to reach them. This is a vital issue: a public health campaign can only succeed if the messages are delivered to the relevant population (4). We address this topic by identifying the media sources unvaccinated Americans tended to use during the COVID-19 pandemic.

We focus specifically on media that other research identifies as being likely venues. Most notably, this includes social media, which has received an enormous amount of attention since the start of the pandemic (5). We look closely at Facebook, given that the size of its user community far exceeds all other social media except for YouTube (6), and they put forth clear efforts to promote COVID-19 preventive behaviors (including partnering with the World Health Organization).^1^ Scholars also frequently discuss Facebook as a possible haven for general vaccine resistance (4, 7–9). These contradictory trends—efforts to promote healthful behavior while facing criticism for being a key place of resistance—make Facebook a crucial outlet to study. The company ostensibly has the intent to promote vaccination (and hence is a promising platform for interventions), but its services may appeal to those who intend to not vaccinate.

We additionally study the presence of the unvaccinated on the major cable news networks—Fox, MSNBC, and CNN—all of which saw their ratings increase dramatically with the pandemic while providing starkly distinct coverage (10). Of particular interest is Fox News given prior evidence that viewership correlated with opposition to a host of COVID-19 preventive attitudes and behaviors (11–14). Taking into account the general skepticism expressed by right-wing media, we also look at Newsmax, a far-right cable news and digital media outlet. Finally, we evaluate seeking direct information from the Trump and Biden administrations, who held frequent briefings followed by many Americans.

To be clear, we are agnostic as to potential causal mechanisms. It could be that particular media lead to vaccine resistance and/or that skeptics choose these sources (due to their distrust in mainstream media and political institutions or because of another factor orthogonal to vaccination). In fact, as we will explain, we suspect that selection of media by those who do not trust vaccines likely shapes the relationship rather than individuals being influenced by media. Regardless, our interest lies in identifying where the unvaccinated reside in the media ecosystem, a question that has received surprisingly little empirical attention.

By identifying the media locations of the unvaccinated, we not only offer insight about COVID-19 vaccination, which will continue to be a challenge for the foreseeable future, but we also show where those who seem distrustful of the medical and scientific establishment congregate. Our findings suggest that trust is a key mechanism of media selection. This information serves an important purpose for those interested in reaching low-trust, vaccine-resistant individuals with any medical or health communication. Put another way, we identify the media that seem most essential as locations to pursue public health campaigns and provide some insight into what strategies may be most effective.

## Analytic considerations in studying media and vaccination

We are interested in the relationship between COVID-19 vaccination and the consumption of relevant COVID-19 information from various media. We focus on COVID-19 information because our interest lies in mechanisms by which the nonvaccinated attend to information directly relevant to the disease (and potentially vaccination). This means our media measures need to capture *exposure* to and *consumption* of *COVID-19 information*. Doing so is notoriously difficult. In our survey, we ask, “In the last 24 hours, did you get any news or information related to the current coronavirus (COVID-19) outbreak from the following sources?,” with answer options including Facebook, Fox News, Newsmax, MSNBC, CNN, and The Biden administration (the first wave of our data preceded Biden, and thus we asked about the Trump administration in that wave). If the respondent did not choose any of the sources, we coded them as “none of these,” which is a category of interest given that these difficult to reach individuals may have low institutional trust (15) and may be less attentive to current affairs.

This particular phrasing of the media question serves several purposes. First, it captures both exposure *and* consumption of COVID-19 information. This latter point is crucial since people access media for widely varying purposes, and we care about COVID-19; moreover, questions about specific events are easier to answer and tend to be well calibrated (16, 17). Second, by asking about consumption in the previous 24 h, we reduce the cognitive load on respondents and minimize the likelihood and extent of overreporting (18). Third, the measure is agnostic to the precise means by which the respondent accessed the given source. This is crucial since consumers may encounter information from, say, a given cable news outlet on television, on the outlet's web page, in that outlet's app, on a social media news feed, by clicking on a link shared by a friend, or via various other channels, etc. (see Appendix Fig. D5 for results showing the range of avenues by which respondents in our data report consuming information from Fox, CNN, and Newsmax).

**Fig. 1.**
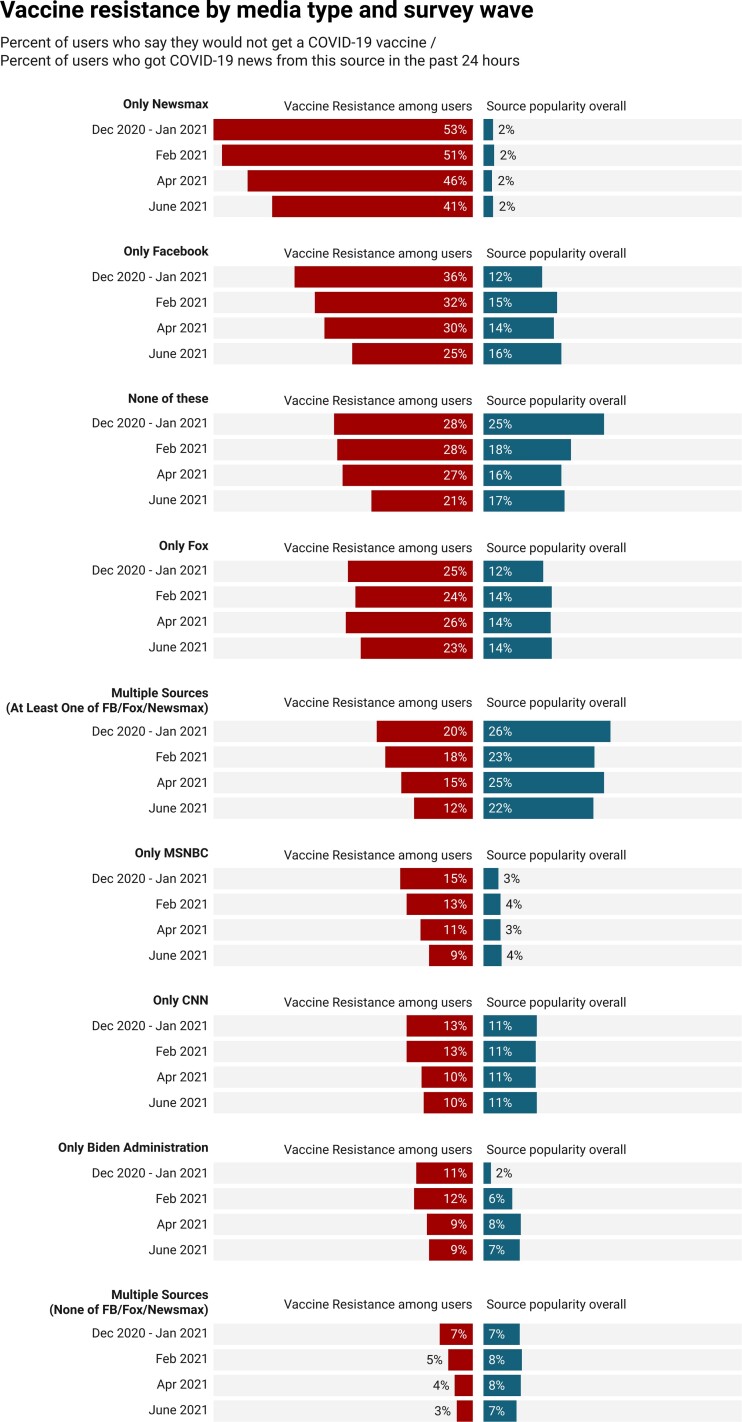
Proportions indicating vaccine refusal by media type and survey wave. Left bars reflect the proportion of respondents in the given media type who were vaccine resistant in the given wave; right bars reflect the relative size of each media-type group by wave. For example, 36, 32, 30, and 25% of the Facebook-only group reported vaccine resistance, and this group constituted 12, 15, 14, and 16% of all respondents, in each respective wave. The Newsmax-only group reported vaccine resistance rates of 53, 51, 46, and 41%, respectively, but only accounted for ∼2% of respondents in each wave.

The measure is of course not perfect. The most notable threat concerns accuracy with the possibility of respondents’ misreporting exposure and consumption (19, 20). This possibility is difficult to definitively assess. For example, we find that 12 to 16% use only Facebook, which is similar to the percentage who report using only Fox (see Fig. 1). Yet, behavioral data suggest that television news exposure far outweighs online news access via news URLs (21, 22). These comparisons, though, focus on news outlets, whereas we include any information on COVID-19 from Facebook, regardless of whether it came from news per se (see Appendix A for a discussion of the various components of Facebook). For instance, the modal Facebook information sources are posts from friends and family (see Appendix Fig. D3). It also could be that COVID-19-specific information access patterns differ from those for general news or political news. Further, our measures are extremely reliable. Among those who responded to our surveys in multiple waves, we find correlations well >0.80 in terms of naming a given source in both waves, even though respondents could reasonably have distinct answers given sources can change (see Appendix Tables D1 and D2).

We present additional secondary data that employ distinct question wordings and sampling frames to assess the robustness of our initial findings. These varying measures are not necessarily identical to ours (i.e. not always specifying the purpose of the media or the same timeframe as our measure). Nonetheless, they allow us to assess the replicability of the findings from our original data with distinct samples. In short, any measure of media consumption, certainly self-reports, is imperfect and should not be interpreted as a literal point estimate as they surely are inexact. However, the steps we take provide confidence that our measures capture where people generally consume COVID-19 information, allowing us to assess which audiences are likelier to include vaccine-resistant US adults.^2^

We also carefully considered the most appropriate measure of vaccination behavior. Our survey asked respondents what best describes their vaccination status, with answers including fully vaccinated, vaccinated with one dose and waiting for the second, not vaccinated but planning on it, not vaccinated and still deciding, and not vaccinated and will not get vaccinated. In Appendix F (Figs. F1–F4), we compare our vaccination rate responses (i.e. having received at least one dose) with administrative data from the Center for Disease Control (CDC). The data are overall comparable. For example, after 2021 April, the state-level first- and second-dose percent data correlate at 0.80 or higher (Figs. F5–F8). Moreover, for reasons we explain in Appendix F (and in contrast to prior findings regarding survey-based vaccination estimates (23)), the differences between our survey-based estimates of vaccination rates and the CDC's administrative records are at least partially attributable to difficulties in linking booster shots with initial doses in the administrative data. This results in the CDC reporting implausibly high vaccination rates in some states following the introduction of booster shots (in some cases exceeding 100%). We thus have confidence in our vaccine measure.

## Results

We base our primary analyses on a multiwave online survey, called the COVID States project. It includes ∼20,000 respondents per wave, recruited via the survey vendor PureSpectrum using quota samples designed to approximate state demographics and are further reweighted to national benchmarks where applicable (see Materials and methods). This approach typically produces good approximations to national trends (24, 25), although below we also will use secondary data from probability samples.

Four consecutive waves of this survey contain the aforementioned COVID-19 media question.^3^ Respondents are grouped into media types according to which, and how many, of the sources they select: none, one (and if so, which), or multiple (grouped by whether at least one of the multiple sources was Facebook, Fox News, or Newsmax). Consistent with prior work (3, 26–28), we define respondents as being vaccine resistant if they report both that they are not vaccinated and that, if they could choose when to receive a COVID-19 vaccine, they would not take one.

Figure 1 shows wave-by-wave proportions of vaccine resistance by source (see Appendix Tables E1–E5 for a descriptive overview of the compositions for each media-type group). Specifically, the “only Newsmax” group reports the highest levels of vaccine resistance but is a much smaller percentage of the population than the others (as shown by the percentages to the right of the bars in Fig. 1). We also find that the “only Facebook” group is consistently among the most vaccine resistant. Interestingly, the “none of these” group—respondents who indicated that they did not access any of the listed media for COVID-19 information—registers relatively high levels of resistance.^4^ This is a heterogeneous group, comprised of a mix of individuals who may turn to alternative media sources, friends and family members, or no sources at all for COVID-19 information.

**Fig. 2.**
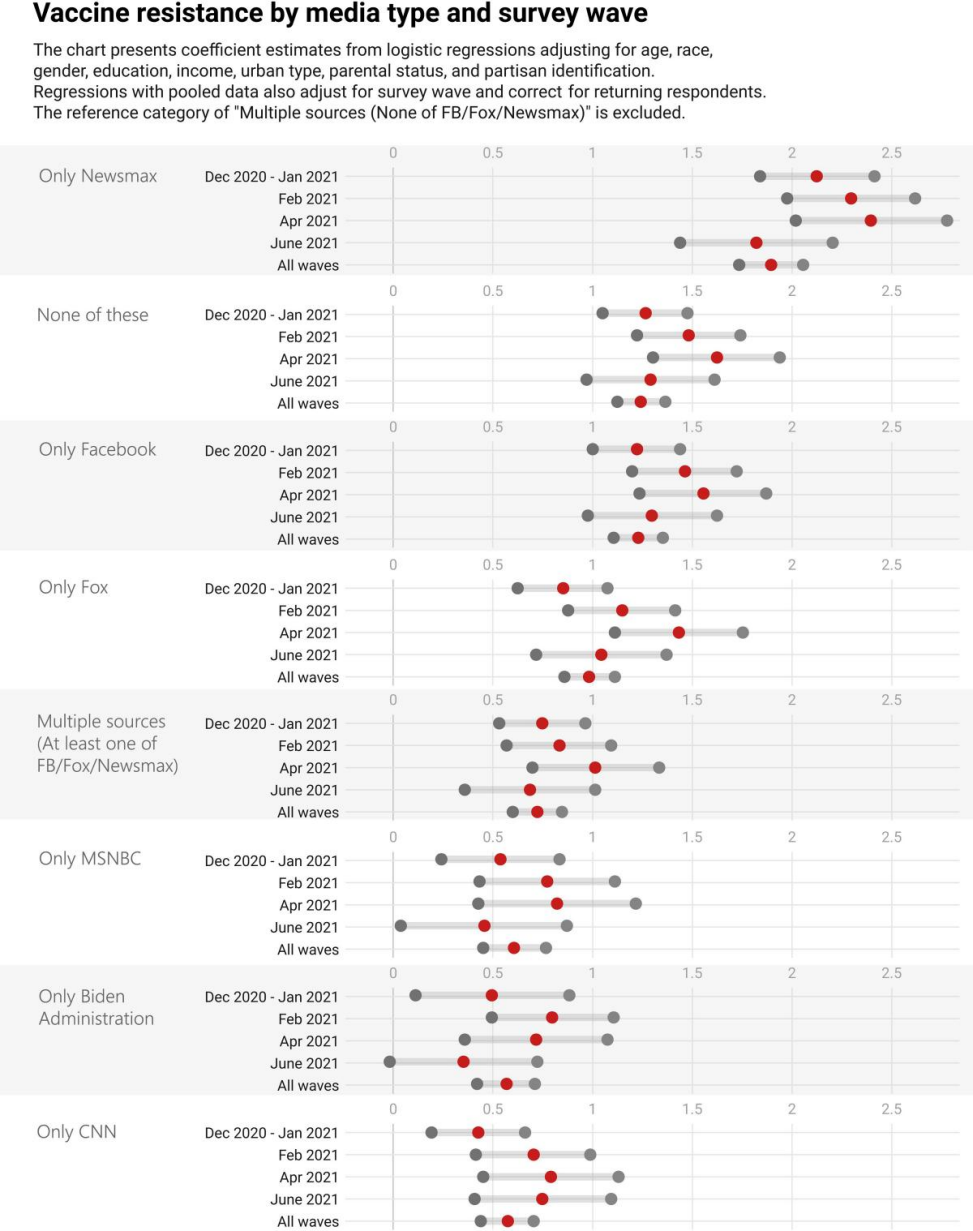
Regression coefficients (middle dots) and 95% uncertainty intervals (boundary dots) for media type by survey wave. Coefficients can be interpreted as the change in log odds of refusing COVID-19 vaccination when moving from this reference media type to the media type described by the coefficient. We include media type as the key independent variable of interest, using the category with the lowest rate of vaccine resistance (i.e. multiple sources and none of Facebook/Fox News/Newsmax) as the reference category. We estimate identical models for each of the four separate survey waves, controlling for census region, race, gender, age, education, income, urban type, parental status, and partisan identification (full regression tables are included in Appendix Tables B1–B5). The fifth model combines data from these waves, adjusting for wave and correcting for returning respondents using a general estimating equation (29).

We build on these descriptive results using a series of logistic regressions, with vaccine resistance as the outcome (and accounting for sociodemographic and political characteristics) (see Appendix Tables B1–B5 for the regressions). We present the results in Fig. 2. The findings are stark and support the descriptive statistics reported in Fig. 1. Those who only obtained COVID-19 information from Newsmax consistently exhibit the most vaccine-resistant attitudes. Those who only relied on Fox News also exhibit consistent vaccinate resistance, albeit not to the same degree. These results reflect the well-known partisan polarization over COVID-19 in general and the vaccine in particular (2). Notably, as in Fig. 1, those who reported use of Facebook and no other source for COVID-19 information in the previous 24 h are significantly more likely to be vaccine resistant (relative to the reference group of respondents who reported consuming information from multiple sources, none of which were Facebook, Fox, or Newsmax). The Facebook result suggests that interventions would benefit from focusing on Facebook, given the large vaccine resistance relationship and the sizeable number of people on the platform. The “none of these” source result is more perplexing since by definition it is not clear where to reach these individuals. That we see some, although significantly less, resistance in the MSNBC, Biden administration, and CNN groups likely reflects the high threshold for the excluded category, which includes people who do not use Facebook or the conservative outlets but receive information from some mix of all of these (provaccine) sources.

We next analyzed respondents who appeared in the earliest survey wave, did not report being vaccinated in that wave,^5^ and returned for at least one subsequent wave.^6^ For this set of individuals, we estimate the probability of being vaccinated in the last wave in which they responded as a function of the media type reported in the initial wave.^7^ We adjust for self-reported intentions to vaccinate and other sociodemographic and political characteristics from the initial wave as well as which wave represents the most recent response for that respondent.^8^ We are thus estimating the extent to which initial media types are associated with subsequent vaccination after accounting for their (increasing) baseline probability of getting vaccinated overall. Put another way, we investigate whether initial consumption choices for COVID-19 information are associated with subsequent vaccination behaviors, controlling for initial vaccine enthusiasm/resistance. These panel data offer an even stronger test of where the unvaccinated congregate, though again, despite the over-time component, results cannot be interpreted as causal. Rather, this test identifies whether using particular media at one point in time meant that later the person was less or more likely to be vaccinated (which could be due to the media content or to factors that led them to consume that media in the first place), after accounting for their initial indication of whether or not they intended to vaccinate.

**Fig. 3.**
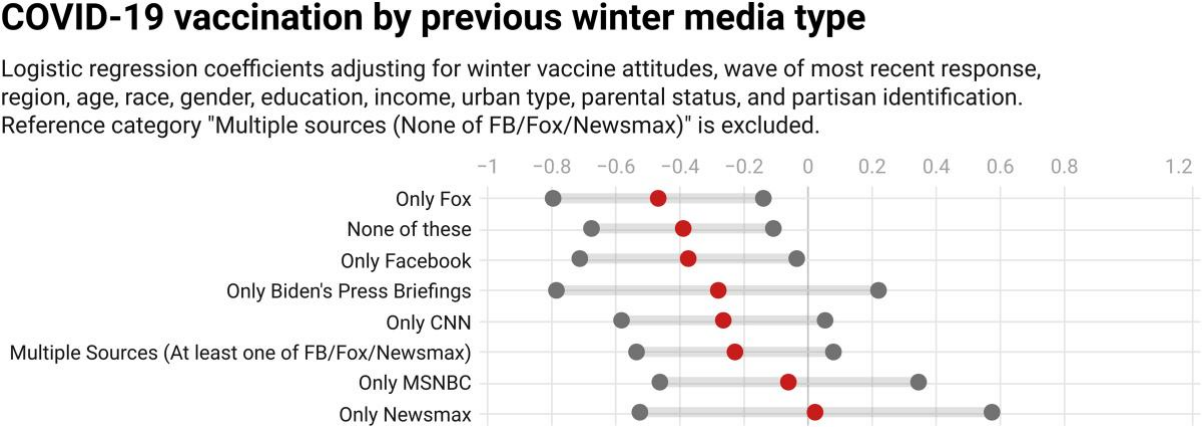
Regression coefficients (middle dots) and 95% uncertainty intervals (boundary dots) for 2020 December to 2021 January media type's relationship with subsequent vaccination. *N* = 6,084 respondents who reported not being vaccinated in the initial wave and returned in a subsequent wave. Regression estimates of vaccination in the most recent wave in which the respondent appeared as a function of baseline demographic covariates, wave of most recent response, and vaccination attitudes in the initial wave.

We find that members of the “only Fox,” “none of these,” and “only Facebook” media types (as measured in winter 2020–2021) are significantly *less* likely to subsequently report being vaccinated than the reference category of “multiple sources, none of Facebook/Fox/Newsmax,” as shown in Fig. 3. Substantively, respondents in the “only Fox,” “none of these,” and “only Facebook” media types in the December to January survey wave were only 63, 68, and 69% as likely, respectively, as those in the reference category to report being vaccinated by the last subsequent wave in which they responded. Of note, the coefficient for “only Newsmax” is near 0 with a high degree of uncertainty, reflecting both its small size (only 132 of 6,084 respondents included in the analysis are in this category) and the high rates of baseline vaccine resistance (61 of these respondents, or 46%, reported in the initial wave that they would not get vaccinated). In this model specification, initial-wave age, college education, income, and (Democratic) partisanship were positively associated with subsequent vaccination, while having children in the household carried a negative association, after accounting for initial-wave vaccination intention (Appendix Table B6). While these panel data offer the strongest test, we next turn to other cross-sectional data sets to further investigate the relationships.

### Kaiser survey analysis

The analyses thus far rely on data collected from nonprobability online samples. Yet, we recognize traditional survey methods emphasize probability samples (30). Thus, we next conduct an analogous analysis using secondary probability-sampled survey data.

We compare the results from our original data with those from a survey conducted by the Kaiser Family Foundation in 2021 January using probability sampling, the data from which are publicly available via the Roper Center's iPoll (see additional details on the sample in Appendix C). This survey asked a series of two-step media consumption questions. Respondents were asked whether they had received information about the COVID-19 vaccine from a given source type (e.g. social media) in the previous 2 weeks. If they answered that they received any information from the given source type, they were asked from which particular sources (e.g. Facebook and Twitter). This item differs from our question but, like ours, has the advantage of specifying a time frame and being COVID-19 specific. The survey also asked respondents for their vaccination status—whether they are currently vaccinated, waiting to see, will get vaccinated if required, or definitely will not be vaccinated (or refused to answer). A useful element of this survey is that it asks about a large range of social media including YouTube, Twitter, Instagram, or another platform not specified. This enables us to assess whether the Facebook dynamic is unique to that platform (a question we address further with our own data below). That said, the sample size for this survey is too small to differentiate these other social media, and thus we group them into “other social media.”

**Fig. 4.**
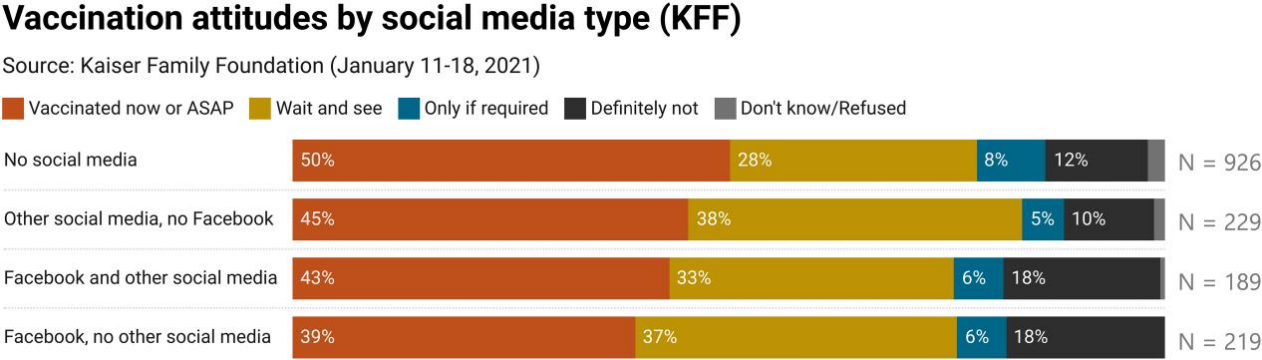
Vaccination attitudes by social media type, Kaiser. Other social media types include YouTube, Twitter, Instagram, or another platform not specified. Cumulative proportions represent within-group distributions of attitudes toward vaccination after applying national-level survey weights. Subgroup sample size, after applying survey weights, is labeled to the right of the bars.

We find that, in the probability sample recruited by Kaiser, Facebook users are more vaccine resistant than those who use other social media. Respondents who reported using Facebook for information about the COVID-19 vaccine in the previous 2 weeks were significantly *less* enthusiastic about and more resistant to taking the vaccine than those who did not. Specifically, 41% of the Facebook group reported either already being vaccinated or intending to get vaccinated as soon as possible; 49% of all other respondents said the same. By contrast, 18% of the Facebook group reported that they would “definitely not” take the COVID-19 vaccine (i.e. vaccine resistant), compared with just 11% of all other respondents and 10% of respondents who reported using other social media sources but not Facebook. These differences are statistically significant at *P* < 0.01. Figure 4 shows cumulative proportions of COVID-19 vaccine attitudes by the full intersection of using Facebook and other sources of social media for related information.

**Fig. 5.**
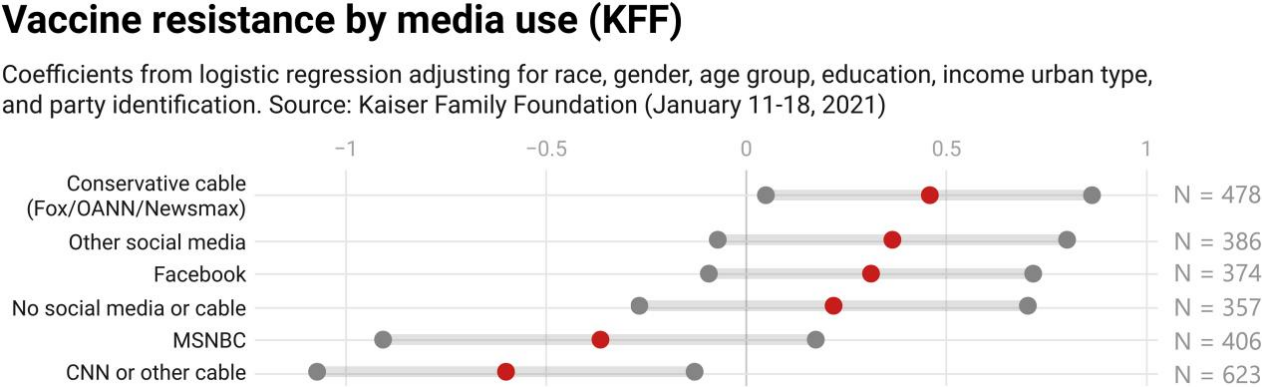
Regression coefficients (middle dots) and 95% uncertainty intervals (boundary dots) for COVID-19 vaccine media sources, Kaiser Family Foundation data.

These data also allow us to compare other media sources including Fox and other conservative outlets, MSNBC, and CNN or other cable media (the results are unchanged if we do not group the other conservative outlets with Fox or other cable with CNN). In Fig. 5, we present results from a multivariate logistic regression with resistance (definitely not get vaccinated) as the outcome variable and the media sources as the independent variables, controlling for available demographic indicators (see Appendix Table B7 for the regressions). We find, again, significant resistance among conservative media users and significant nonresistance among CNN users. We do not, however, see an MSNBC relationship. The association between social media use (both Facebook and other platforms) and vaccine resistance is just short of statistical significance when adjusting for additional forms of media consumption and demographic characteristics, though given the large point estimate and relatively small sample size, it is possible that this is due to a lack of statistical power. Given our prior findings (particularly with the panel data), this association suggests a possible connection that we probe further with yet another data source.

### Real Clear Opinion analysis

**Fig. 6.**
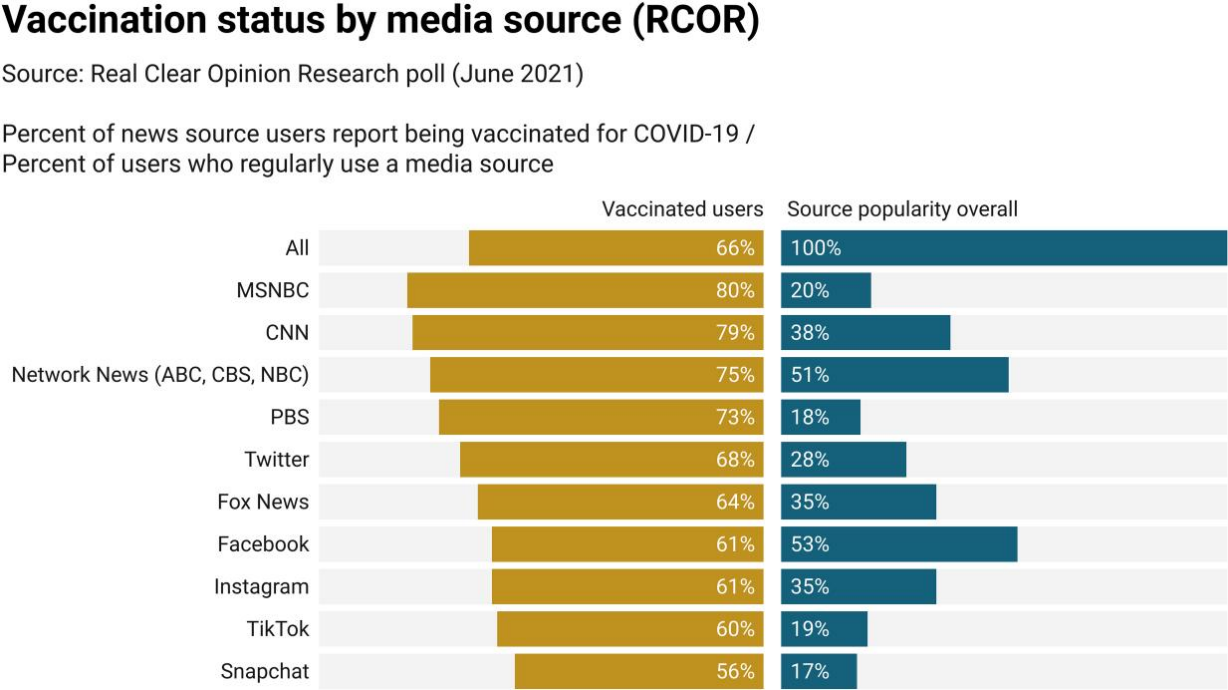
Vaccination status by media source, Real Clear Opinion Research. Individual sources sorted by percent of users reporting vaccination. Source popularity (right) shows the unweighted share of respondents who reported regularly using a source.

We turn to a separate nonprobability, quota-sampled (of registered voters) Real Clear Opinion Research poll conducted in 2021 June (31), the raw data from which were shared with the authors. This survey asked respondents to report regularly using a variety of media sources (thus, it is not ideal since it is not COVID-19 specific) and whether they had received a dose of the vaccine. We present descriptive results in Fig. 6. We find vaccination rates exceeding the national average (66%, reflected in the top bar) among those who relied on CNN or MSNBC (as well as other mainstream networks). Interestingly, Fox News users are not significantly lower than the national average, perhaps reflecting a difference between general users and those who specifically seek COVID-19 information. We also find vaccination rates below the national average among respondents who report regularly visiting Facebook (61%), compared with the national average. We see too, in contrast to the Kaiser descriptive results, that users of other social media (other than Twitter) register vaccine rates similarly low as Facebook.

**Fig. 7.**
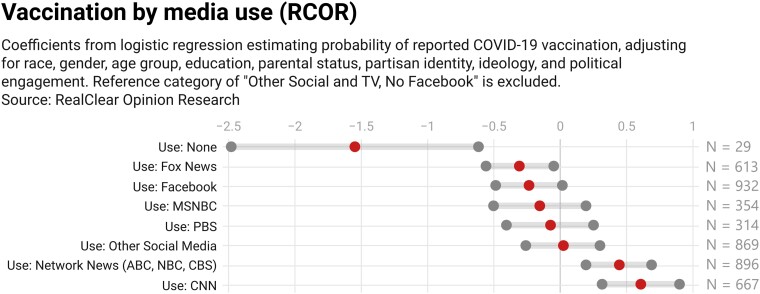
Regression coefficients (middle dots) and 95% uncertainty intervals (boundary dots) for association between media type and vaccination. *N* = 1,762 respondents. Regression also adjusts for race, gender, age group, education, income, urban type, parental status, partisan identification, ideology, and political engagement.

Unlike the Kaiser data, in this instance, a multivariate analysis finds that consuming none of the listed media sources is negatively associated with reported vaccination against COVID-19, though this is a small group of respondents (Fig. 7). Getting information from Fox News is also negatively associated with vaccination against COVID-19; the coefficient for consuming such information from Facebook is negative and marginally short of conventional significance levels (*P* = 0.07 for a two-tailed test). Network television sources, as well as CNN, are positively and significantly associated with vaccination.

### COVID States alternate media consumption item

Thus far, we see a consistent pattern showing that social media seems to be a popular venue among those who are vaccine resistant. The Kaiser results suggested the possibility that Facebook differs from other social media, but the Real Clear Opinion Research findings do not (although again that item did not focus on COVID-19). We sought to address this inconsistency by amending our own question in a fifth survey wave. We replaced the media consumption item described above with two items that ask about a wider range of social media use. For a set of 10 social media sources, we asked respondents to report consumption with respect to COVID-19 information in two ways: whether they used the source for this purpose in the previous 24 h and how important they considered the source generally on a scale from 1 (not at all important) to 4 (very important). As these items ask about a wider range of sources within the category of social media, we do not combine the results with information regarding respondents’ consumption of traditional media.

**Fig. 8.**
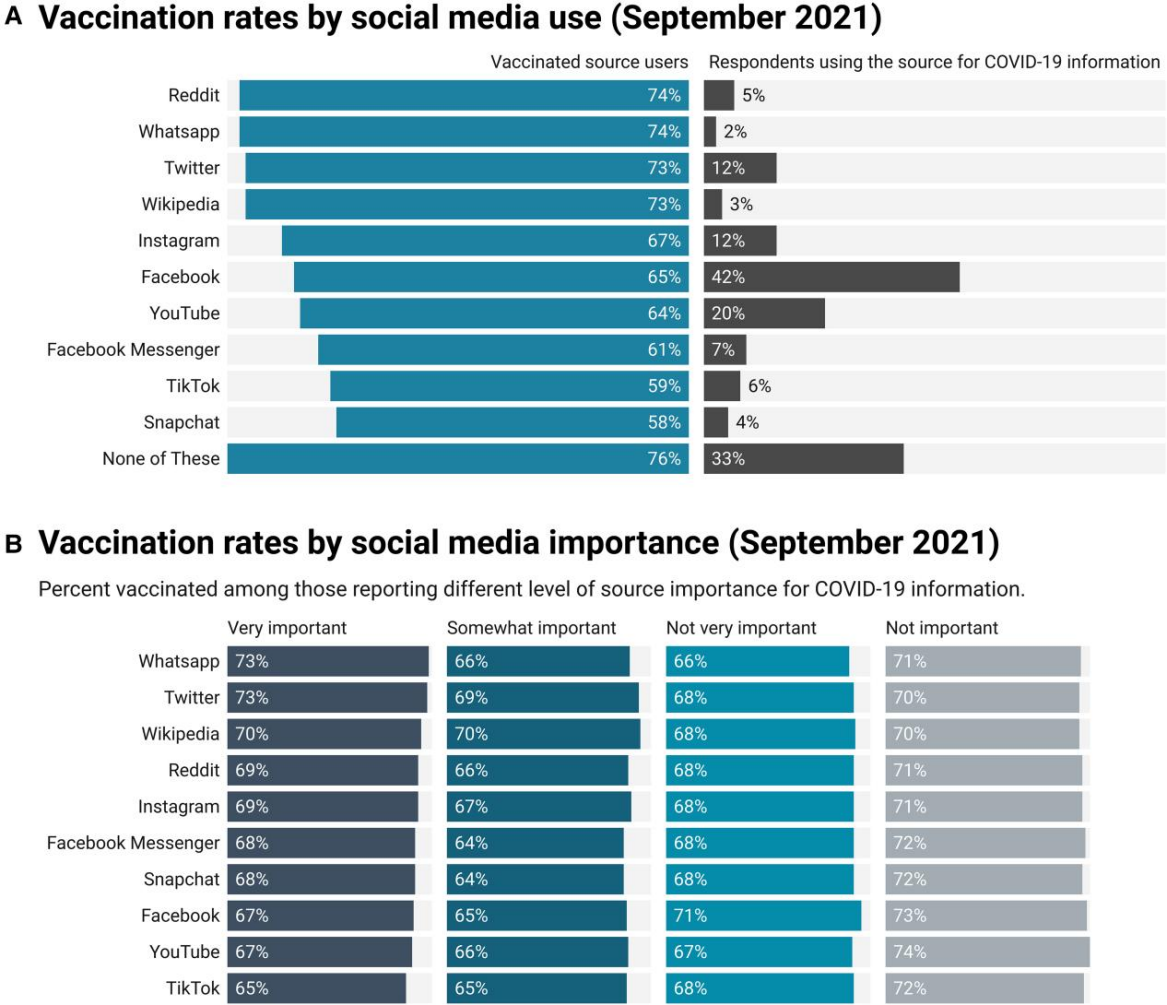
Proportion vaccinated by social media use. A) shows proportion vaccinated among respondents who said they had used the source for information about COVID-19 in the previous 24 h. B) shows the same proportion by reported importance of the source.

Figure 8 shows vaccination rates by reported use in the previous 24 h (panel A) and reported importance of a given source (panel B) (see Appendix Fig. D2 for a more detailed breakdown by age group). Lower scores indicate *more* vaccine hesitancy/resistance. As in waves described in our initial analyses, we find that respondents who used Facebook for COVID-19 information in the previous 24 h reported lower vaccination rates—both compared with respondents who did not report using any such sources and compared with respondents who reported using sites such as Reddit, WhatsApp, Twitter, and Wikipedia. In addition, respondents who reported using Facebook Messenger for COVID-19 information had even lower vaccination rates. Facebook and Facebook Messenger use are also associated with notable and significant differences in vaccination rates between those who consider them to be important sources of information regarding COVID-19 and those who consider them to be unimportant. That said, other social media also draw in the unvaccinated—including YouTube, TikTok, and Snapchat, although the percentages of respondents who report using these latter platforms for COVID-19 information are substantially smaller than for Facebook.

**Fig. 9.**
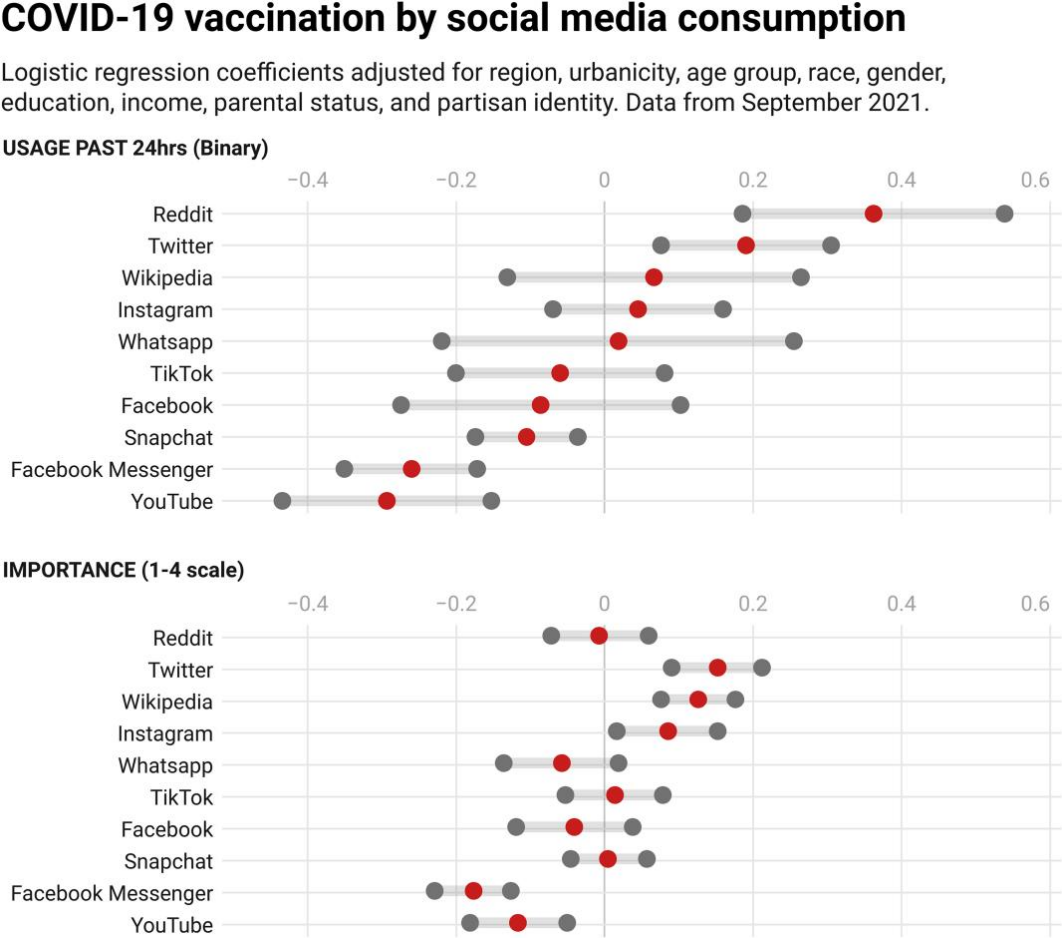
Regression coefficients (middle dots) and 95% uncertainty intervals (boundary dots) for associations between social media use (top) and importance (bottom) and vaccination. *N* = 20,974 respondents who answered recent use battery and 16,563 respondents who answered social media importance battery. Regression also adjusts for census region, race, gender, age, education, income, urban type, parental status, and partisan identification.

Corresponding multivariate results, shown in Fig. 9, show both recent use and perceived importance of Facebook Messenger (as well as YouTube and Snapchat) and are significantly associated with lower probabilities of being vaccinated, but this relationship does not carry over to Facebook itself, which suggests that distinct Facebook platforms may operate differently.

### CBS survey analysis

**Fig. 10.**
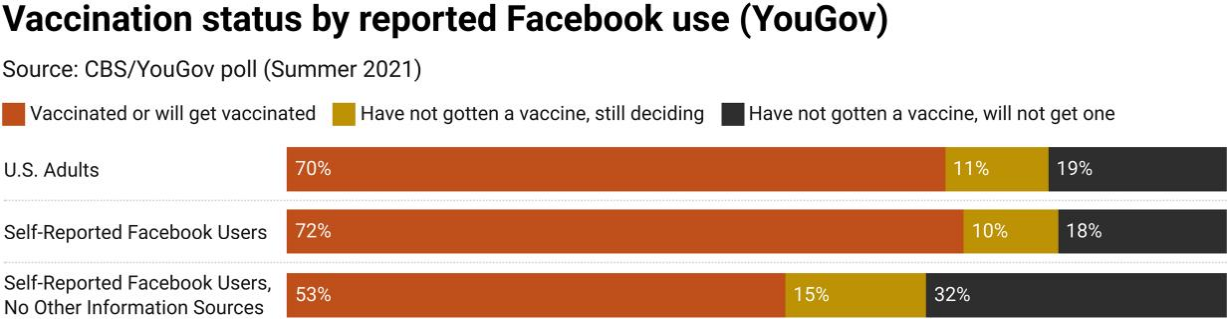
Vaccination status by reported Facebook use, CBC/YouGov poll data.

A final confirmatory analysis comes from a nonprobability, quota-sampled CBS/YouGov survey, presented with their permission, in Fig. 10. Specifically, CBS/YouGov separately provided us with vaccination status/attitudes for all adults in their survey: for those who report using Facebook regularly and for those who report using Facebook *and no other provided sources* regularly (results from corresponding regressions conducted by CBS/YouGov, published with their permission, are included in Appendix Table B9). This is not an ideal measure since, like the Real Clear Opinion Research data, it does not isolate using Facebook specifically for COVID-19 information. Nonetheless, we find that those who rely on Facebook for information are distinct from those for whom Facebook is one of multiple sources of information. Self-reported Facebook users have very similar rates of vaccination and vaccine refusal to the general public (70% vaccinated or will be vaccinated vs. 19% refusing vaccines). However, those for whom Facebook is the *only* provided source they report using regularly have substantially lower vaccination rates (53% vaccinated or will get vaccinated) and substantially higher rates of vaccine refusal (32%).

### Trust as a mechanism

**Fig. 11.**
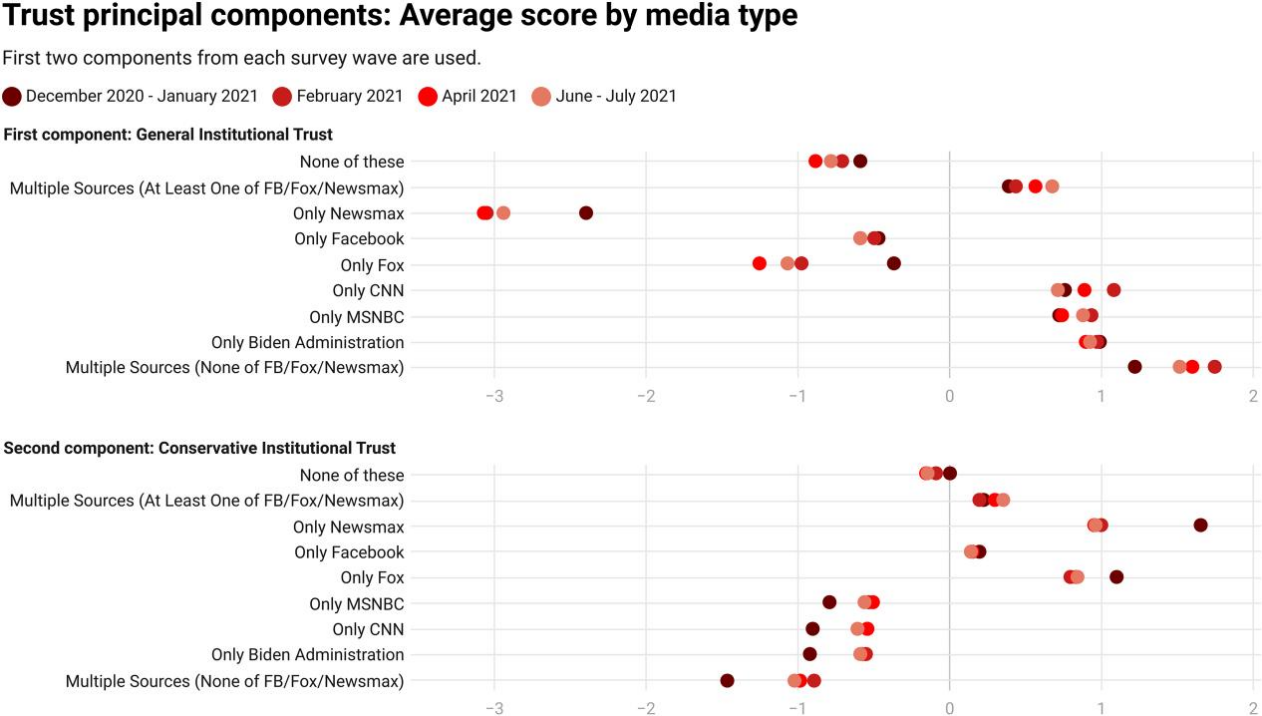
Average loadings for the first two dimensions of principal components of institutional trust battery by media type. The first component reflects general institutional trust (trust in people/institutions such as Congress, the news media, the CDC, the FDA, and Dr. Anthony Fauci load highly on this dimension); the second component reflects trust in more conservative people/institutions (trust in people/institutions such as Donald Trump, the police, and, banks load highly on this dimension). See Appendix E for further discussion.

The pattern of results presents a clear picture: conservative media users (Fox News and other outlets) tend to be vaccine resistant, liberal users (MSNBC and CNN) tend to be vaccinated, and social media (especially Facebook in the absence of other sources) tends to be a hub of vaccine resistance, reaching conventional levels of statistical significance in most cases and approaching it in the rest. We realize that interventions on conservative media may be untenable, but social media seems ripe as a mechanism through which to direct provaccine messaging (32). In so doing, relying on mainstream health institutions as the source may not be effective. Indeed, our surveys included items that asked respondents about their trust in a wide range of institutions that reduce to two dimensions (shown further in Appendix Tables E10 and E11): a general institutional dimension (e.g. trust in the CDC, FDA, Dr. Fauci, and Joe Biden) and a conservative institutional dimension (e.g. trust in the police and Donald Trump; see Jost (32)). In Fig. 11, we show that those who use what turned out to be the vaccine-resistant outlets display low trust in the first dimension that includes prominent public health officials and institutions and more trust in the second dimension that includes police, banks, and Donald Trump. It seems likely that trust is a mechanism that brings together the users and their media choices.

These findings have notable implications when it comes to social media (especially Facebook, which was the focus of our analysis due to its popularity). While this does not rule out misinformation playing a role in people's decisions, it does suggest that those who lack trust select into particular media where they are more or less likely to be exposed to misleading claims. Indeed, low trust and misinformation consumption likely cause one another such that the independent contributions of each to vaccine behaviors are difficult to disentangle. This pattern coheres with other work that suggest the amount of misinformation shared on Facebook is a tiny fraction of its content (20, 34, 35) and that, in fact, social media often promotes knowledge (36). In short, our results suggest that those who do not trust institutions (and may therefore avoid mainstream sources of information) are more likely to receive their COVID-19 information from social media such as Facebook, and their mistrust could contribute to vaccine resistance in a variety of ways. Notably, though, “only Facebook” users exhibit trust that more closely resembles the “none of these” group (i.e. those who did not access the listed media for COVID-19 information) than Fox or Newsmax consumers.

The trust findings do not mean that these individuals are unswayable. Instead, it suggests that messaging on social media such as Facebook may be more effective invoking speakers such as Donald Trump or other authority figures and not health providers or scientists (on the effectiveness of Trump messaging; see Pink et al. and Larsen et al. (2, 32)). These are the actors who the vaccine resistant are likely to trust. Finally, it is telling that those who report not receiving information about COVID-19 from any sources exhibit low levels of trust in the first general institutional dimension but do not tend to be distinctly high or low on the second conservative institutional dimension. This population may be not only hard to reach but also difficult to sway, a conclusion consistent with Lee and Chu's (37) finding that “political outsiders” are notably unvaccinated.

## Conclusion

Our results add to what we know about the media in the public sphere. While we only interrogate a single issue, it is an exceptionally important one with implications for future public health emergencies. Most interestingly, in the case of COVID-19 vaccinations, we find little evidence of social media platforms functioning as places where collective deliberation promotes positive social outcomes. Rather, it seems that, at least in certain contexts, they play a much less salubrious role. Of course, more work is needed, looking at media choice in similar contexts, employing distinct approaches. While we have confidence in our self-report measures, we also recognize that they are imperfect and require complimentary efforts.

The findings presented here do not directly indict social media, as those who rely on it for COVID-19-related information are in many cases distrustful of—or simply not exposed to—such information from credible, mainstream news sources. Indeed, we find that individuals who report not receiving COVID-19 information from traditional news sources (i.e. those in the “only Facebook” or “none of these” groups in our four survey waves) are demographically distinct. Those who only receive COVID-19 information from Facebook are significantly more likely to be White, not college educated, female, lower income, and Republican, have children in their households, and live in rural areas (Appendix Table B12). Those who report not receiving such information from any of the sources we asked about were also significantly more likely to be White, female, and Republican and were significantly less likely to have children in their household (Appendix Table B13). All this is to say that selection into these different media types likely informs the relationships we observe. Those who are less trusting of mainstream institutions and less attentive to current affairs are both less likely to encounter and accept information that would encourage them to get vaccinated.

Our findings accentuate the importance of using technology for social good. The efforts of social media companies such as Facebook may have been quite effective relative to what vaccination resistance would look like without their initiatives. Yet, given the platform's exceptionally broad reach, more interventions should be pursued. The most vital point is that communication strategies to promote positive collective actions need to carefully consider *where* the most reticent reside.

## Materials and methods

### Researcher-collected data

The primary data used for these analyses come from a large semimonthly survey collected by the author team. Participants for this survey are recruited via PureSpectrum, using quota sampling to approximate the demographic composition of each of the 50 US states and the District of Columbia. Participants included for analyses are those that passed two closed-ended and one open-ended attention check and passed additional checks for speeding and straightlining.

The resulting sample sizes for each wave used in our primary analyses are as follows:

- 2020 December to 2021 January: 25,640
- 2021 February: 21,500
- 2021 April: 21,733
- 2021 June to July: 20,669

For results that rely on individual survey waves (i.e. all between-subjects analyses), we report cross-tabulated results from these data using national weights, which are set based on national benchmarks for race/ethnicity, age, gender, education, and living in urban/suburban/rural areas. Regression results do not use survey weights.

We also conduct within-subjects analysis using a panel of respondents who completed the 2020 December to 2021 January wave and at least one other wave between then and the 2021 June to July wave, identifying returning respondents using the unique respondent ID provided by PureSpectrum. We only conduct regression analyses on this subset of respondents and do not reweight them.

Finally, we conduct secondary analyses on additional survey waves separately. The timing and sample sizes for those waves are as follows:

- 2021 August to September: 21,079
- 2021 November to December: 22,277
- 2021 December to 2022 January: 22,961
- 2022 March to April: 22,234

### Public data

We reanalyze data collected by the Kaiser Family Foundation, who fielded a probability-sampled telephone survey of 1,563 adults between 2021 January 11 and 18. This survey included an oversample of 332 prepaid (pay-as-you-go) telephone numbers and was reweighted to national benchmarks for the US population.

These data are publicly available through the Roper Center under study number 31118182.

We report cross-tabulated results from this survey that use their national survey weight and regression results using the unweighted data. Question wordings for relevant items in this survey are included in Appendix C.

### Shared data

We report cross-tabulated and regression-based results from data shared with our research team by Real Clear Opinion Research. This survey is a national sample of 1,762 registered voters, quota-sampled via Lucid from 2021 June 21 to 24, weighted to national benchmarks for the US population.

### External results published with permission

We report cross-tabulated results shared with our research team, and published with permission, from CBS News. This survey is a national quota sample of 2,238 adults recruited via YouGov's panel from 2021 July 14 to 17, weighted to national benchmarks for the US population.

## Supplementary Material

pgad146_Supplementary_Data

## Data Availability

Data and code necessary to reproduce results in this manuscript can be found on the Harvard Dataverse: https://doi.org/10.7910/DVN/3EYFML. Additional data collected by CBS/YouGov may be shared at the organization’s discretion (contact Kabir Khanna at KhannaK@cbsnews.com).

## Footnotes

1 See https://www.who.int/news/item/11-08-2021-who-facebook-and-praekelt.org-provide-critical-mobile-access-to-covid-19-information-for-vulnerable-communities and https://about.meta.com/actions/responding-to-covid-19/.

2 We recognize potential partisan overreporting for some of the partisan outlets we include, particularly Fox News and Newsmax. We suspect overreporting on these outlets might occur among stronger Republicans who also tend to be vaccine hesitant (2), thus potentially inflating the correlation with vaccine hesitancy. See Appendix Tables E2–E5 for demographic characteristics of respondents in each media type group by wave.

3 For the purposes of these analyses, if we refer to the administration, we are discussing the “Biden administration,” (we exclude that item from the initial wave when Trump was in office), unless we are specifically referring to the initial wave alone.

4 Unlike the only Newsmax group, this group comprises a sizable share of the population. This partially reflects that many Americans do not regularly access news, including on social media (28).

5 As very few people had the opportunity to be vaccinated by 2021 January, nearly all respondents in this wave reported not being vaccinated.

6 We removed 87 respondents who either reported inconsistent age and ZIP code information when they returned or reported not being vaccinated after having reported being vaccinated. These filters and alternate specifications are discussed further in Appendix Table B6.

7 As moving from being open to vaccination to being vaccine resistant is a rare event, accounting for just 231 (4%) of respondents in the panel, we do not use vaccine resistance as our outcome for this portion of the analysis.

8 In Appendix Table B6, we show that adjusting for the national vaccination rate at the time of this most recent response instead of the wave of the most recent response does not substantively change our results.
