# Correction to: Media use and vaccine resistance

**DOI:** 10.1093/pnasnexus/pgad376

**Published:** 2023-11-15

This is a correction to: Jon Green, James N Druckman, Matthew A Baum, Katherine Ognyanova, Matthew D Simonson, Roy H Perlis, David Lazer, Media use and vaccine resistance, *PNAS Nexus*, Volume 2, Issue 5, May 2023, pgad146, https://doi.org/10.1093/pnasnexus/pgad146

During a retroactive audit conducted by *PNAS Nexus*, it was discovered that this paper was missing a statement acknowledging compliance with the *PNAS Nexus* Human and Animal Participants and Clinical Trials policy:

All data collection and procedures in this manuscript were approved by the institutional review boards at Northeastern (#20-04-12), Rutgers (#Pro2020000977), and Harvard (#IRB20-0593) Universities. All participants in the study provided their informed consent.

This error has been corrected in the original article.

