# Supplementary material for: Media use and vaccine resistance
Source: PNAS Nexus. 2023 May 9;2(5):pgad146. doi: 10.1093/pnasnexus/pgad146 (PMC10178922; doi:10.1093/pnasnexus/pgad146)
Supplement: pgad146_Supplementary_Data [file pgad146_supplementary_data.zip › PNASNEXUS-PNASNEXUS-2022-00931-s05.pdf]

Appendix A: Theoretical and Analytical Considerations in the Study of Media

Discussion of Alternative Media Measures

In the text, we briefly discussed our use of survey self-reports as opposed to measuring media exposure using objective metrics. Here we elaborate on that topic; to be clear, we agree that in many cases, objective metrics offer a superior approach. However, in our case, the survey measures had certain advantages, as elaborated upon in the text. One of the main objective approaches employ digital trace data that tracks behavior. For example, Muise et al. (2022) use Nielsen panels for both television and web browsing behavior, finding that partisan segregation on television is far more extensive that in on-line news consumption, and TV news audiences are generally far larger than online news audiences. For example, they report that 10% of all TV news consumers nearly exclusively use Fox. Allen et al. (2020) use desktop, mobile, and television news data and gauge passive consumption using imputation based on referrals, finding users on average spend three times as many minutes per day consuming television news (not counting local news) than on-line news.

These approaches serve the authors' purposes well and provide important points of comparison (also see, e.g., Parry et al. 2021; Konitzer et al. 2021), but the strategy has some downsides in our specific case. First, some of the estimates are imperfect at capturing consumption; even Allen et al.'s admirable imputation approach relies on the assumption that referrals consistently correspond with impressions (and consumption/engagement), and only capture news consumption accompanied by a referring URL. Exposure does not imply consumption (Conrad, Keusch, and Schober 2021; Konitzer et al. 2021), and users frequently encounter information on social media sites that is not associated with a link to a specific news source. Second, users can often access particular sources in a number of ways. As mentioned in the text, consumers may encounter information from, say, a given cable outlet on television, on the outlet's web page, in that outlet's app, on a social media news feed, by clicking on a link shared by a friend, or via various other channels. This diversity of access points are typically not captured by behavioral data (Barthel et al. 2020). Third, our interest in COVID-19 specific information complicates behavioral data too since we would need knowledge of specific content. Such acute topical focus likely increases the accuracy of our measures. For instance, Guess et al. (2019) study a construct closer to ours, looking at social media activity. They conclude, "The good news is that self-reports are correlated with observed behavior… self-reports of social media use are meaningful; they are (perhaps surprisingly) accurate and correlated with our objective measure…" (254; also see Settle 2018).

To be clear, we recognize our survey measure is far from perfect and in many cases behavior measures are preferable. We simply suggest that, for us, survey measures have some advantages. One final note is that we recognize that our audience size estimates may strike some as inaccurate. For instance, we estimate that 12-14% of the population consume COVID-19 information exclusively from Fox while other work suggests 8%-10% of Americans are majority Fox watchers (Allen et al. 2020). This (relatively modest in terms of percentage points) difference could be due to at least three possible factors. First, given our focus, we asked about a

limited number of sources. It is possible that some of our exclusive Fox watchers actually obtain a majority of their information from local or national mainstream networks—put another way, "Fox only" refers to "only" relative to the sources about which we asked. Second, we focus explicitly on COVID-19 information whereas the other estimates are more general. Third, the lower estimate comes from Nielsen data, but it is possible that people consume Fox News outside of their home (see Appendix D). Others may suspect, as noted in the text, that we over-estimate Facebook consumption given audience sizes for television typically substantially outpace on-line news (and we find similar levels) (Allen et al. 2020). Muise et al. (2022) suggest TV outweigh on-line by a factor of 5 to 1, although it is important to note that that ratio refers to how many Americans are segregated into politically homogeneous environments on television versus the internet and not absolute consumption (our focus). Allen et al (2020) report that television, sans local news, has audiences three times as large as online. The main point of difference though is its focus on news specifically whereas we care about any COVID-19 news or information on Facebook. As we show in Appendix D, users report encountering news and information about COVID-19 on Facebook via a variety of in-platform sources, some of which may include an external URL of the sort that would be captures in Allen et al.'s imputation but many of which would not be.

Another approach to studying media is to implement an experiment where the researcher controls exposure and observes consumption; however, our interest lies in choices made in naturalistic settings and thus even an experiment with media choice likely would not emulate day-to-day exposure that often comes as a by-product. This approach also focuses on media affecting preferences whereas we suspect that selection into media was the process at work in our case. This vital point aside, even if one were to believe an impact of media on preferences, the experimental approach would have downsides in this context. Many such experiments incentivize (randomly assigned) individuals to deactivate their accounts (Allcott et al. 2020) or access particular content (Levy 2021). These designs are not straightforwardly applicable to our focus for a few reasons (aside from selection processes). For one, there is a strong likelihood of a pre-treatment effect such that participants already have formed vaccine preferences (i.e., even before vaccines were available), and thus a lack of a treatment effect (e.g., those who deactivated do not become more open to vaccines) could reflect that individuals already formed strong (hesitancy) intentions due to prior Facebook exposure (and short-term deactivation is insufficient). This is a notable threat when it comes to highly salient, personally relevant attitudes (Druckman and Leeper 2012), such as those involving health decisions. Thus, a null result from this design could be misleading since the effect already occurred. Further, since deactivation means no exposure, it cannot test if a distinct intervention would alter vaccine resistant attitudes (i.e., interventions could still matter quite a bit but would not be detected by a deactivation design). Another problem with a deactivation design is self-selection into the study such that those prone to be more vaccine hesitant also tend to be less trusting of institutions and more likely to be removed from the political process (Hegland et al. 2022). Such individuals who skew right also tend to be less likely to participate in surveys (Kennedy et al. 2021); while this includes the surveys we analyze, it likely would be more acute in a study that requires consent to

control over which media one may access. In short, a deactivation design does not apply straightforwardly to the study of social media and vaccine resistance due to the likelihood of pre-treatment and study selection effects. And, perhaps most important of all, as mentioned, we actually suspect that selection into media is the underlying process rather than media affecting preferences.

What is Facebook?

In studying Facebook, one must articulate exactly what it is, as "Facebook" is many things. It sometimes supports person-to-person connections, sometimes enables a broadcast from one to millions, and so on. It affords four distinct channels of information flow that have very different properties: groups, pages, friends, and advertising. Facebook Messenger, embedded within Facebook, also allows person-to-person communication. Groups involve essentially limitless communications from many to many, with the largest groups having millions of members. Pages entail an information flow from one to many with the most sizable pages including more than 100 million followers and quasi-broadcast functionality. In principle, Facebook users could directly go to friends', groups', or pages' profiles (while ads are shown to them directly). However, scholars typically assume that information largely flows from these sources to individuals via Feeds (formerly News Feeds) and that most users have vast inventories of content they could potentially receive from these four channels. That said, Feeds algorithmically choose a sequence of content from the groups, pages, and friends to which a user is connected. They also select ads eligible for users to see, with opaque machine learning methods and optimands (presumably, in part, promoting continued engagement with the site). Facebook Feeds, in turn, have an unambiguous relationship with information consumption, where content within Feeds provides glimpses of content that may provoke further engagement such as shares, clicks, comments, and reactions. The cumulative effect of those glimpses may be cognitively consequential (though there is little research on this question). Facebook, in short, constitutes a complex communication system that is used in extremely heterogeneous ways. (See Appendix Figure D3 for a descriptive overview of the different channels through which individuals report seeing COVID-19 information on Facebook).

References

Allcott, Hunt, Luca Braghieri, Sarah Eichmeyer, and Matthew Gentzkow. 2020. "The Welfare Effects of Social Media." *American Economic Review* 110 (3): 629–76.

Allen, Jennifer, Baird Howland, Markus Mobius, David Rothschild, and Duncan J. Watts. 2020. "Evaluating the Fake News Problem at the Scale of the Information Ecosystem." *Science Advances* 6 (14): eaay3539. https://doi.org/10.1126/sciadv.aay3539.

Barthel, Michael, Amy Mitchell, Dorene Asare-Marfo, Courtney Kennedy, and Kirsten Worden. 2020. "Measuring News Consumption in a Digital Era." Pew Research Center. https://www.pewresearch.org/journalism/2020/12/08/measuring-news-consumption-in-a-digital-era/.

Conrad, Frederick G., Florian Keusch, and Michael F. Schober. 2021. "New Data in Social and Behavioral Research." *Public Opinion Quarterly* 85 (S1): 253–63.

Druckman, James N., and Thomas J. Leeper. 2012. "Learning More from Political Communication Experiments: Pretreatment and Its Effects." *American Journal of Political Science* 56: 875–96.

Guess, Andrew, Kevin Munger, Jonathan Nagler, and Joshua Tucker. 2019. "How Accurate Are Survey Responses on Social Media and Politics?" *Political Communication* 36 (2): 241–58.

Hegland, Austin, Annie Li Zhang, Brianna Zichettella, and Josh Pasek. 2022. "A Partisan Pandemic: How COVID-19 Was Primed for Polarization." *The Annals of the American Academy of Political and Social Science* 700 (1): 55–72.

Kennedy, Courtney, Jesse Lopez, Scott Keeter, Arnold Lau, Nick Hatley, and Nick Bertoni. 2021. "Confronting 2016 and 2020 Polling Limitations." Pew Research Center. https://www.pewresearch.org/methods/wp-content/uploads/sites/10/2021/04/PM_04.08.21_polling.limitations.pdf.

Konitzer, Tobias, Jennifer Allen, Stephanie Eckman, Baird Howland, Markus Mobius, David Rothschild, and Duncan Watts. 2021. "Comparing Estimates of News Consumption from Survey and Passively Collected Behavioral Data." *Public Opinion Quarterly* 85 (S1): 347–70.

Levy, Ro'ee. 2021. "Social Media, News Consumption, and Polarization." *American Economic Review* 111 (3): 831–70.

Muise, Daniel, Homa Hosseinmardi, Baird Howland, Markus Mobius, David Rothschild, and Duncan J. Watts. 2022. "Quantifying Partisan News Diets in Web and TV Audiences." *Science Advances* 8 (28): eabn0083. https://doi.org/10.1126/sciadv.abn0083.

Parry, Douglas A., Brittany I. Davidson, Craig J. R. Sewall, Jacob T. Fisher, Hannah Mieczkowski, and Daniel S. Quintana. 2021. "A Systematic Review and Meta-Analysis of Discrepancies between Logged and Self-Reported Digital Media Use." *Nature Human Behaviour* 5 (11): 1535–47. https://doi.org/10.1038/s41562-021-01117-5.

Settle, Jamie. 2018. *Frenemies: How Social Media Polarizes America*. Cambridge University Press.
