# Supplementary material for: Media use and vaccine resistance
Source: PNAS Nexus. 2023 May 9;2(5):pgad146. doi: 10.1093/pnasnexus/pgad146 (PMC10178922; doi:10.1093/pnasnexus/pgad146)

Vaccine resistance by media type and survey wave

Percent of users who say they would not get a COVID-19 vaccine /
Percent of users who got COVID-19 news from this source in the past 24 hours

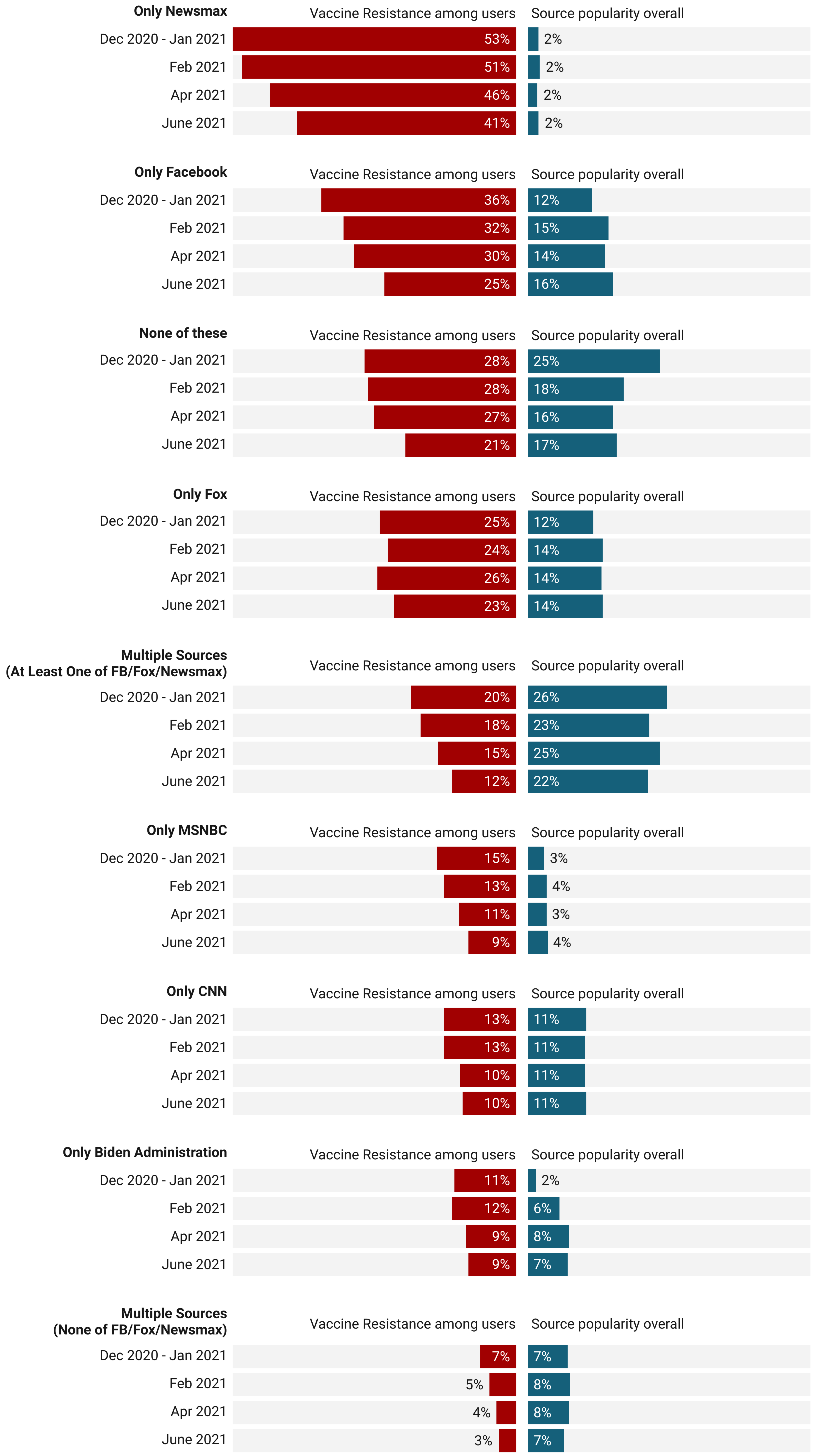

Supplement: pgad146_Supplementary_Data [file pgad146_supplementary_data.zip › PNASNEXUS-PNASNEXUS-2022-00931-s06.pdf]
