# Supplementary material for: Media use and vaccine resistance
Source: PNAS Nexus. 2023 May 9;2(5):pgad146. doi: 10.1093/pnasnexus/pgad146 (PMC10178922; doi:10.1093/pnasnexus/pgad146)
Supplement: pgad146_Supplementary_Data [file pgad146_supplementary_data.zip › PNASNEXUS-PNASNEXUS-2022-00931-s01.pdf]

Appendix F: Vaccination rates

The data used in this study was collected by the COVID States Project (covidstates.org). This multi-university initiative collects detailed state-level information about the coronavirus pandemic, its impact on communities, and the public attitudes related to the disease. The project uses data from a large-scale online non-probability survey with census-based quotas, controls for data quality, and post-stratification weighting.

Previous research has suggested that large-scale non-probability surveys have data problems (Bradley et al, 2021), including significantly overestimating vaccination rates compared to data from the Centers for Disease Control and Prevention (CDC).

In this appendix, we compare data from seven survey waves of the COVID States Project to CDC vaccination data. We show that our data tends to underestimate CDC percentages for one and two administered doses, and overestimate those for booster doses. At the same time, our state-level data is highly correlated with CDC estimates.

Important to note, the estimates of vaccination rates offered by CDC have significant issues. For example, for certain states (including New Hampshire, Rhode Island, Massachusetts, Connecticut, New Jersey, and New York), the CDC estimates for the number of adults with at least a single shot of the COVID-19 vaccine exceeds the number of people living in those states. The CDC does not offer a full explanation as to why this is the case, but does note, in an explanation as to why coverage metrics are capped at 95%:[1]

> *When possible, CDC links a person's first, second, and booster doses together. However, linking is sometimes not possible because CDC does not receive personally identifiable information about vaccine doses. This can lead to over-estimates of first doses and under-estimates of subsequent doses.*

This explanation does fit the pattern of deviations of estimates of the vaccination rates from the COVID States Project, as discussed below. This pattern of over/under estimates of vaccination rates, yielding sometimes impossible numbers and unknown heterogeneity in error at the state level suggests that caution should be exercised in using the CDC and state reports of vaccination rates as ground truth, or to calibrate surveys.

The COVID States Project data shown here comes from seven survey waves described in Table A1 below. Our surveys use state-level representative quotas for race/ethnicity, age, and gender. In addition to balancing on these dimensions, the data is reweighted to match the U.S. population with respect to race/ethnicity, age, gender, education, living in urban, suburban, or rural areas.

Throughout this appendix, estimates from survey data collected over a particular time period are compared to CDC data averaged over the same period. Figures F1, F2, and F3 compare COVID

---

[1]From: https://covid.cdc.gov/covid-data-tracker/#vaccinations_vacc-people-onedose-pop-5yr, April 30th, 2022, clicking "The percent of the population coverage metrics are capped at 95%. Learn how CDC estimates vaccination coverage." for a pop up window).

States survey data with CDC data for national percent of US adults who have received one, two, or three doses of the COVID-19 vaccine.

Table F1. *Data used in this appendix*

Short wave name	Total N	Time period	Data on dose 1	Data on dose 2	Data on dose 3
Feb-21	21,500	02/05/2021-03/01/2021	YES	NO	NO
Apr-21	21,733	04/01/2021-05/03/2021	YES	YES	NO
Jun-21	20,669	06/09/2021-07/07/2021	YES	YES	NO
Sep-21	21,079	08/26/2021-09/27/2021	YES	YES	YES
Nov-21	22,277	11/03/2021-12/02/2021	YES	YES	YES
Jan-22	22,961	12/22/2021-01/24/2022	YES	YES	YES
Mar-22	22,234	03/02/2022-04/04/2022	YES	YES	YES

The average difference between CDC's and our estimates of percent Americans who received vaccines is -8% for one dose, -7% for two doses, and +4% for three vaccine doses. As Figures F1-F3 show, the differences between COVID States Project data and CDC data start small and increase over time as CDC increasingly overcounts first and second vaccine doses. During the time period of our last survey wave, for instance, the CDC data included 9 states where first vaccine doses were seemingly administered to over 100% of the population. Over time, CDC-estimated first vaccine doses for some states in our data reached as high as 115% (for New Hampshire).

We further compared our estimates state by state to those of the CDC for the same time period. In almost all cases, our state data *underestimates* the CDC percentages for people who received one or two doses, and *overestimates* those for a third dose. The mean differences between COVID States and CDC data are relatively smaller for the first four waves of our survey (-4 to 3 percentage points for one dose, -4 to -2 for two doses). After the US population starts receiving boosters, the observed differences increase (-15 to -8 for one dose, -5 to -7 for two doses, +8 to +10 percentage points for boosters). Figure F4 demonstrates these patterns.

In addition, state-level first- and second- dose percent data is highly and significantly correlated between the COVID States Project and CDC after March 2021. The correlation is .63 or higher in April 2021; and higher than .80 for all following waves. The booster data is correlated at .34 in November 2021, .57 in January 2022, and .74 in March 2022. Those patterns are presented on Figures F5 to F7.

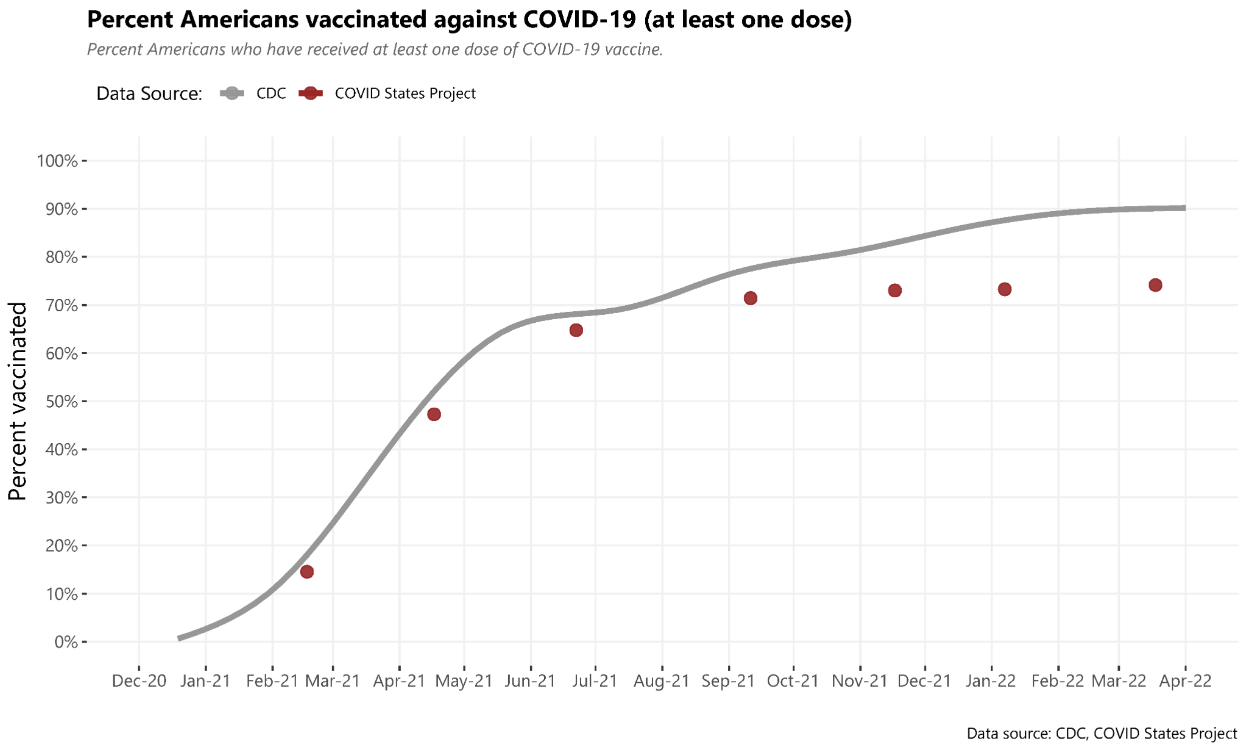

Percent Americans vaccinated against COVID-19 (at least one dose)
Percent Americans who have received at least one dose of COVID-19 vaccine.
Data Source:
CDC
COVID States Project
100%
90%
80%
70%
60%
50%
40%
30%
20%
10%
0%
Percent vaccinated
Dec-20
Jan-21
Feb-21
Mar-21
Apr-21
May-21
Jun-21
Jul-21
Aug-21
Sep-21
Oct-21
Nov-21
Dec-21
Jan-22
Feb-22
Mar-22
Apr-22
Data source: CDC, COVID States Project

Figure F1.

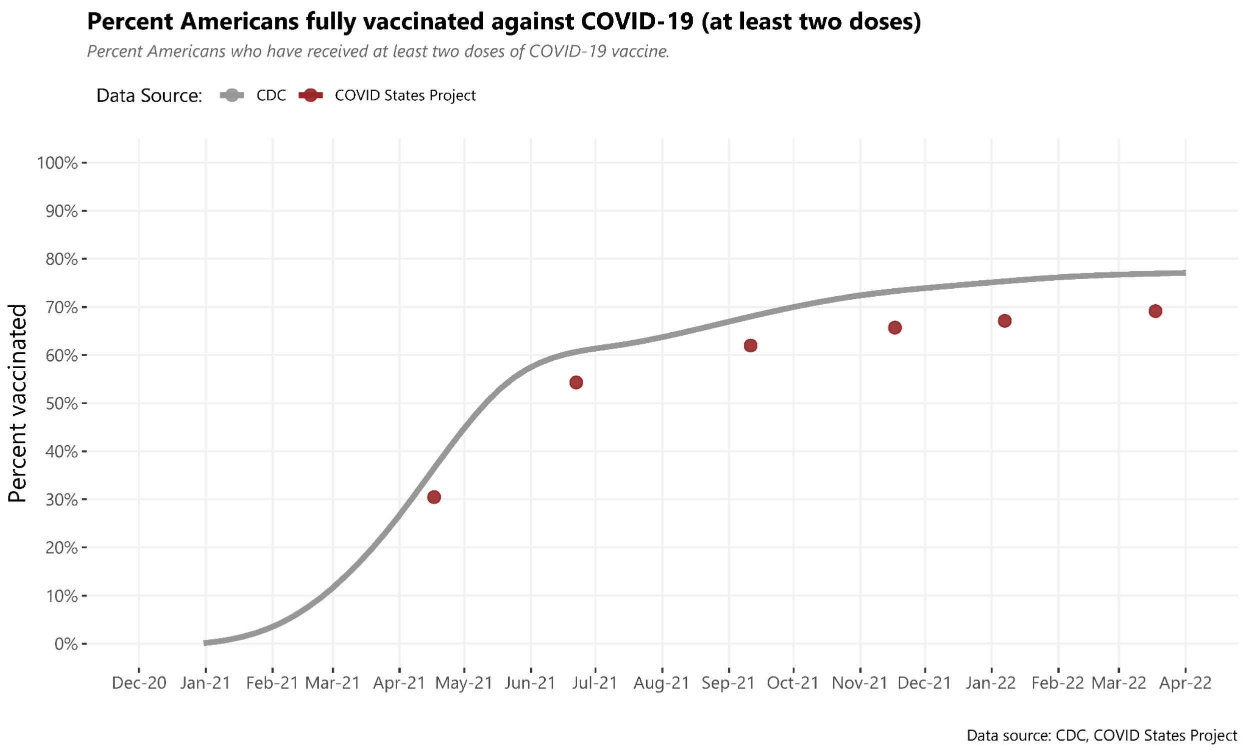

Figure F2.

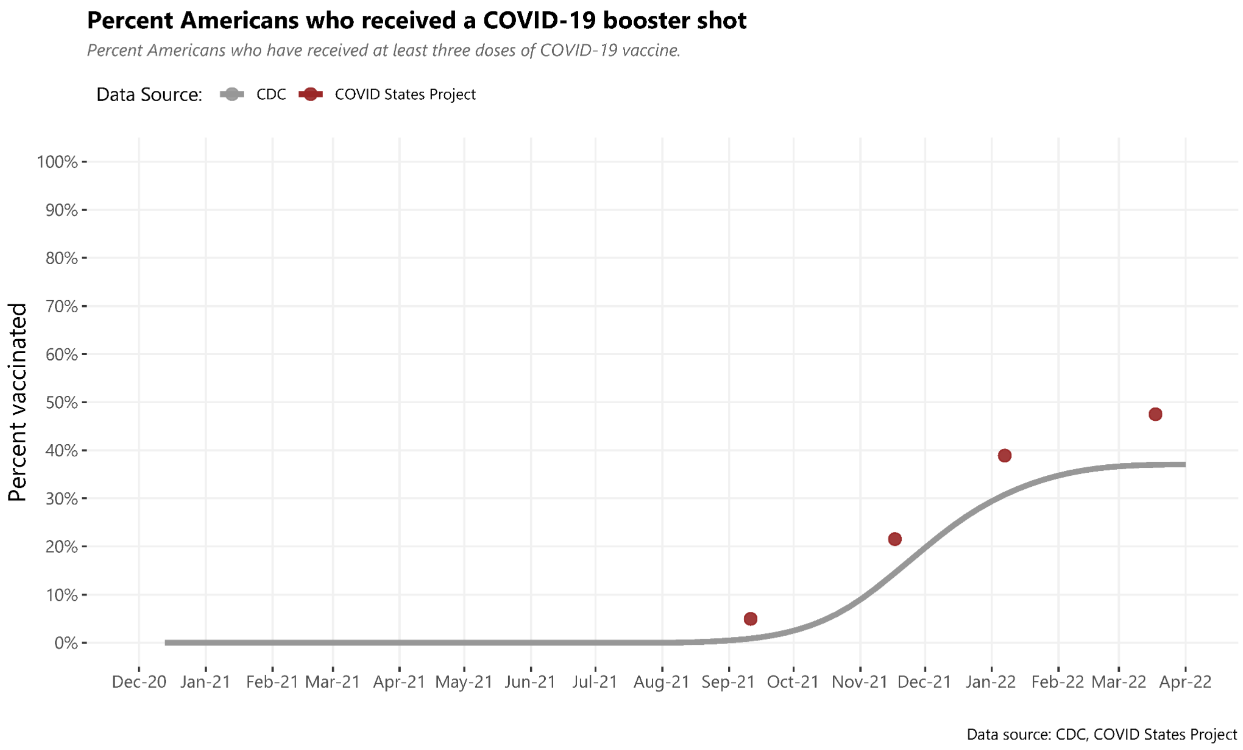

Figure F3.

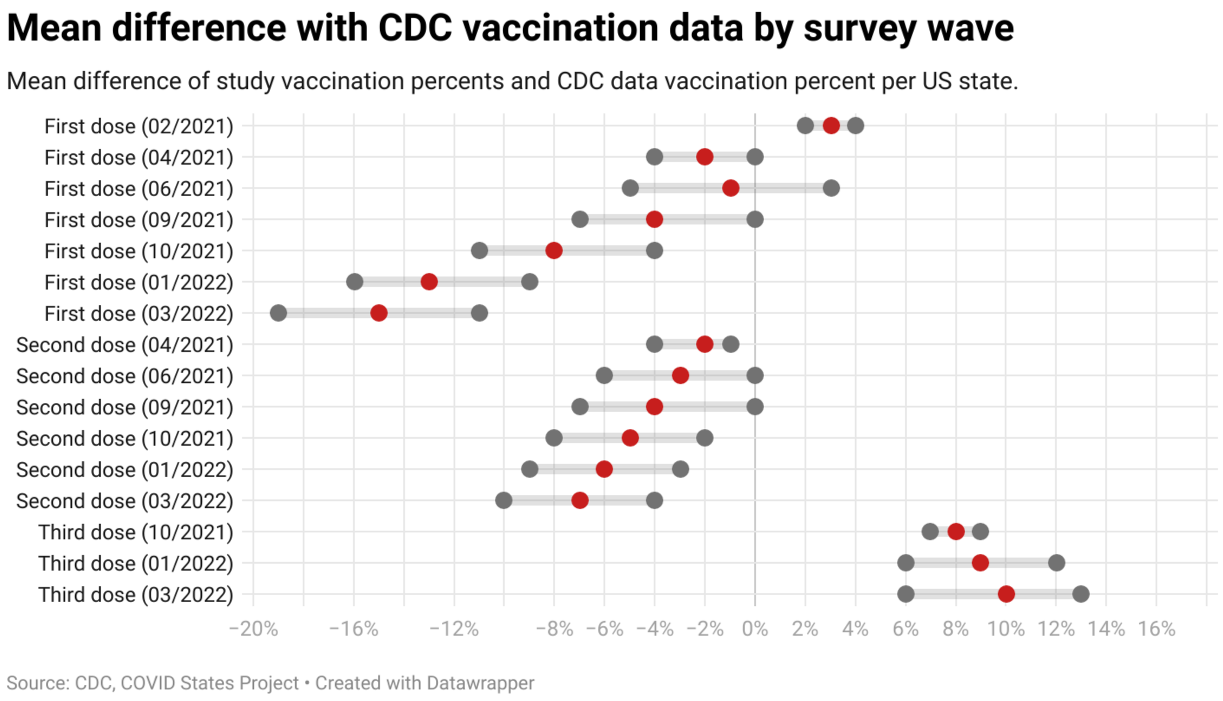
Mean difference with CDC vaccination data by survey wave
Mean difference of study vaccination percents and CDC data vaccination percent per US state.
First dose (02/2021)
First dose (04/2021)
First dose (06/2021)
First dose (09/2021)
First dose (10/2021)
First dose (01/2022)
First dose (03/2022)
Second dose (04/2021)
Second dose (06/2021)
Second dose (09/2021)
Second dose (10/2021)
Second dose (01/2022)
Second dose (03/2022)
Third dose (10/2021)
Third dose (01/2022)
Third dose (03/2022)
−20%
−16%
−12%
−8%
−6%
−4%
−2%
0%
2%
4%
6%
8%
10%
12%
14%
16%
Source: CDC, COVID States Project • Created with Datawrapper

Figure F4.

Adults vaccinated against COVID-19 by state (first dose)

The figure shows percent adult residents in each US state vaccinated against COVID-19. The vertical axis uses CDC numbers (cdc.gov). The horizontal axis uses data from the COVID States Project. Colors represent the times at which the data was collected.

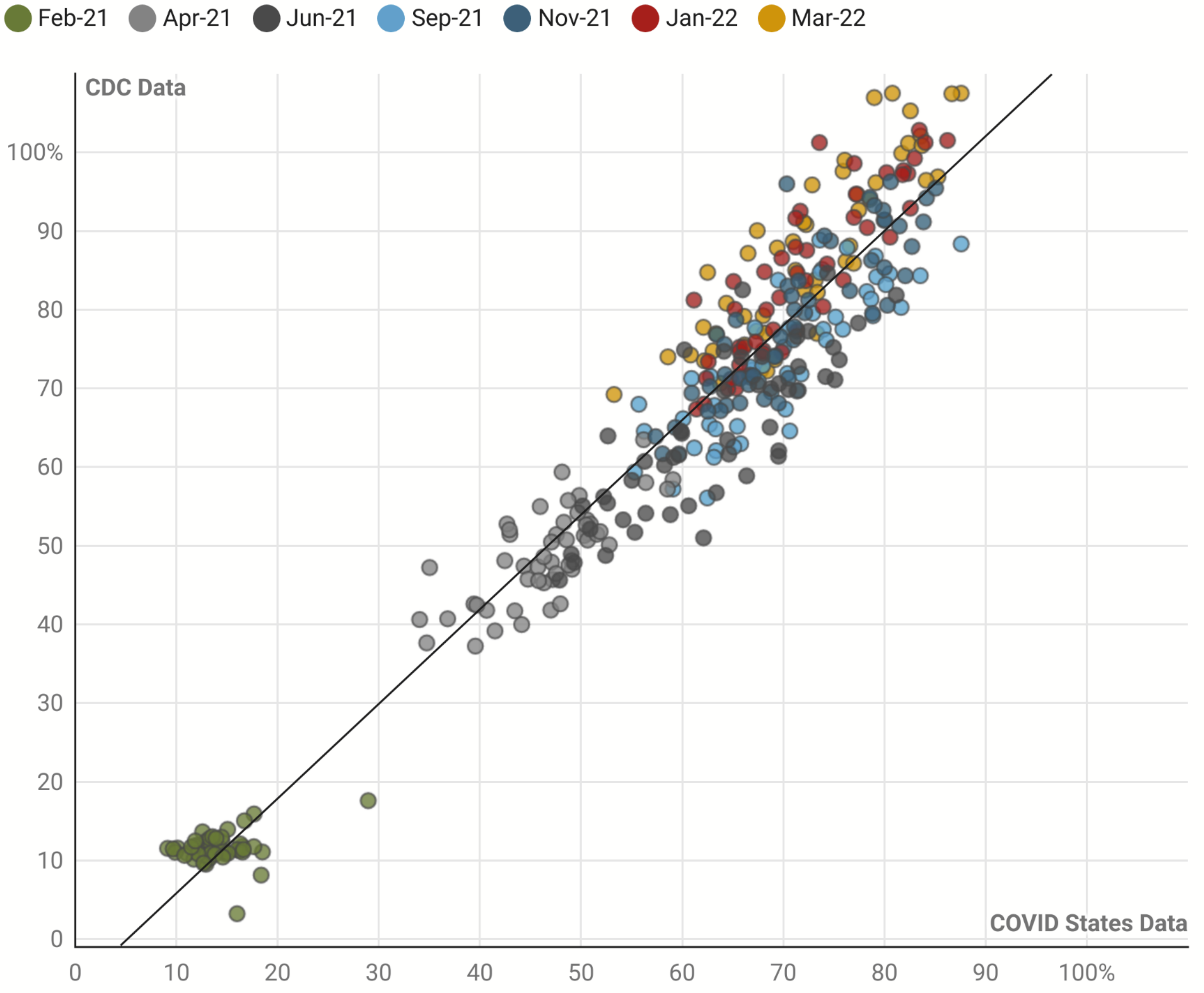

Feb-21: N = 21,500, Time period: 02/05/2021-03/01/2021; Apr-21: N = 21,733, Time period: 04/01/2021-05/03/2021; Jun-21: N = 20,669, Time period: 06/09/2021-07/07/2021; Sep-21: N = 21,079, Time period: 08/26/2021-09/27/2021; Nov-21: N = 22,277, Time period: 11/03/2021-12/02/2021; Jan-22: N = 22,961, Time period: 12/22/2021-01/24/2022; Mar-22: N = 22,234, Time period: 03/02/2022-04/04/2022

Source: CDC, COVID States Project • Created with Datawrapper

Figure F5.

Adults vaccinated against COVID-19 by state (second dose)

The figure shows percent adult residents in each US state vaccinated against COVID-19. The vertical axis uses CDC numbers (cdc.gov). The horizontal axis uses data from the COVID States Project. Colors represent the times at which the data was collected.

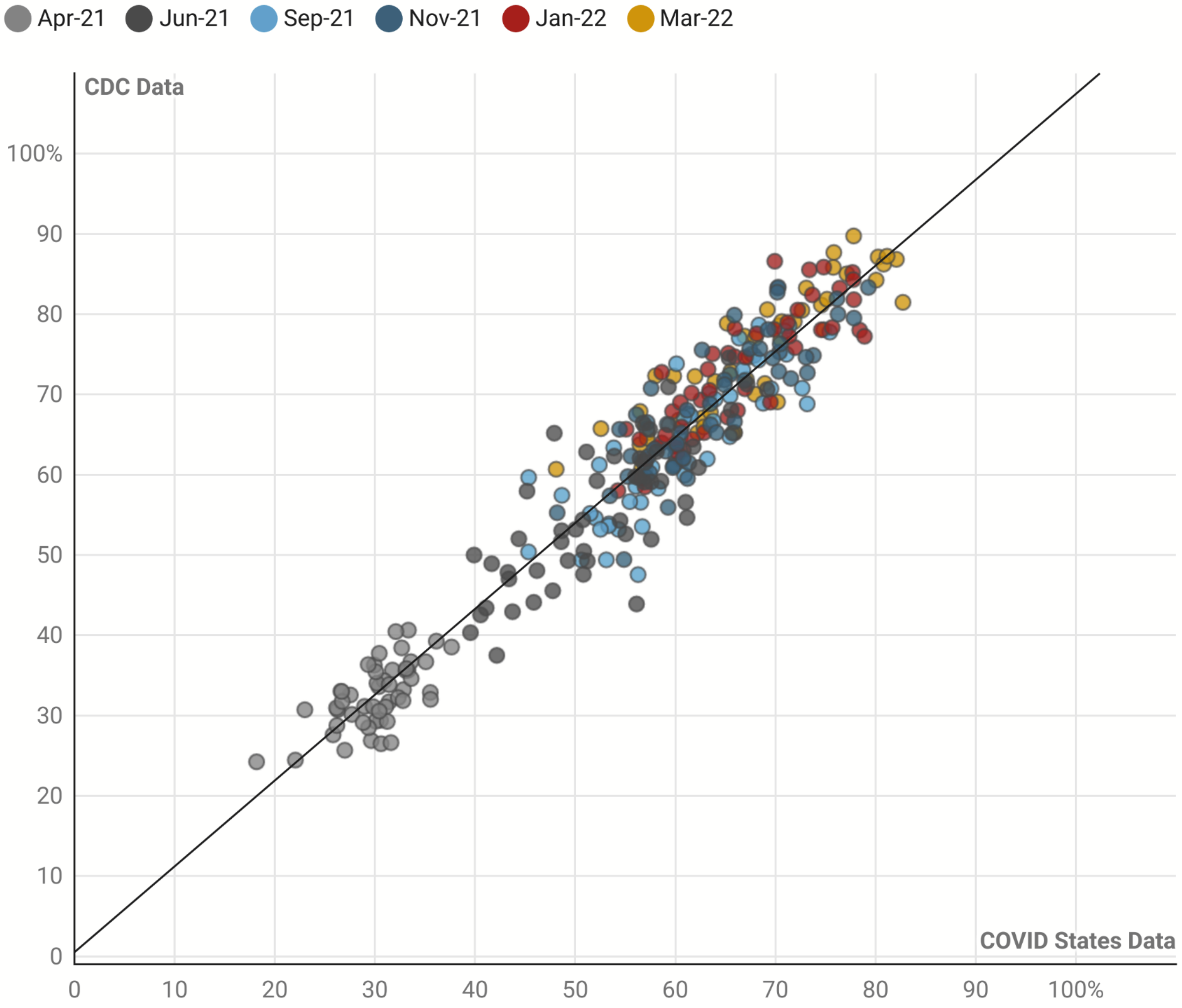

Apr-21: N = 21,733, Time period: 04/01/2021-05/03/2021; Jun-21: N = 20,669, Time period: 06/09/2021-07/07/2021; Sep-21: N = 21,079, Time period: 08/26/2021-09/27/2021; Nov-21: N = 22,277, Time period: 11/03/2021-12/02/2021; Jan-22: N = 22,961, Time period: 12/22/2021-01/24/2022; Mar-22: N = 22,234, Time period: 03/02/2022-04/04/2022

Source: CDC, COVID States Project • Created with Datawrapper

Figure F6.

Adults vaccinated against COVID-19 by state (third dose)

The figure shows percent adult residents in each US state vaccinated against COVID-19. The vertical axis uses CDC numbers (cdc.gov). The horizontal axis uses data from the COVID States Project. Colors represent the times at which the data was collected.

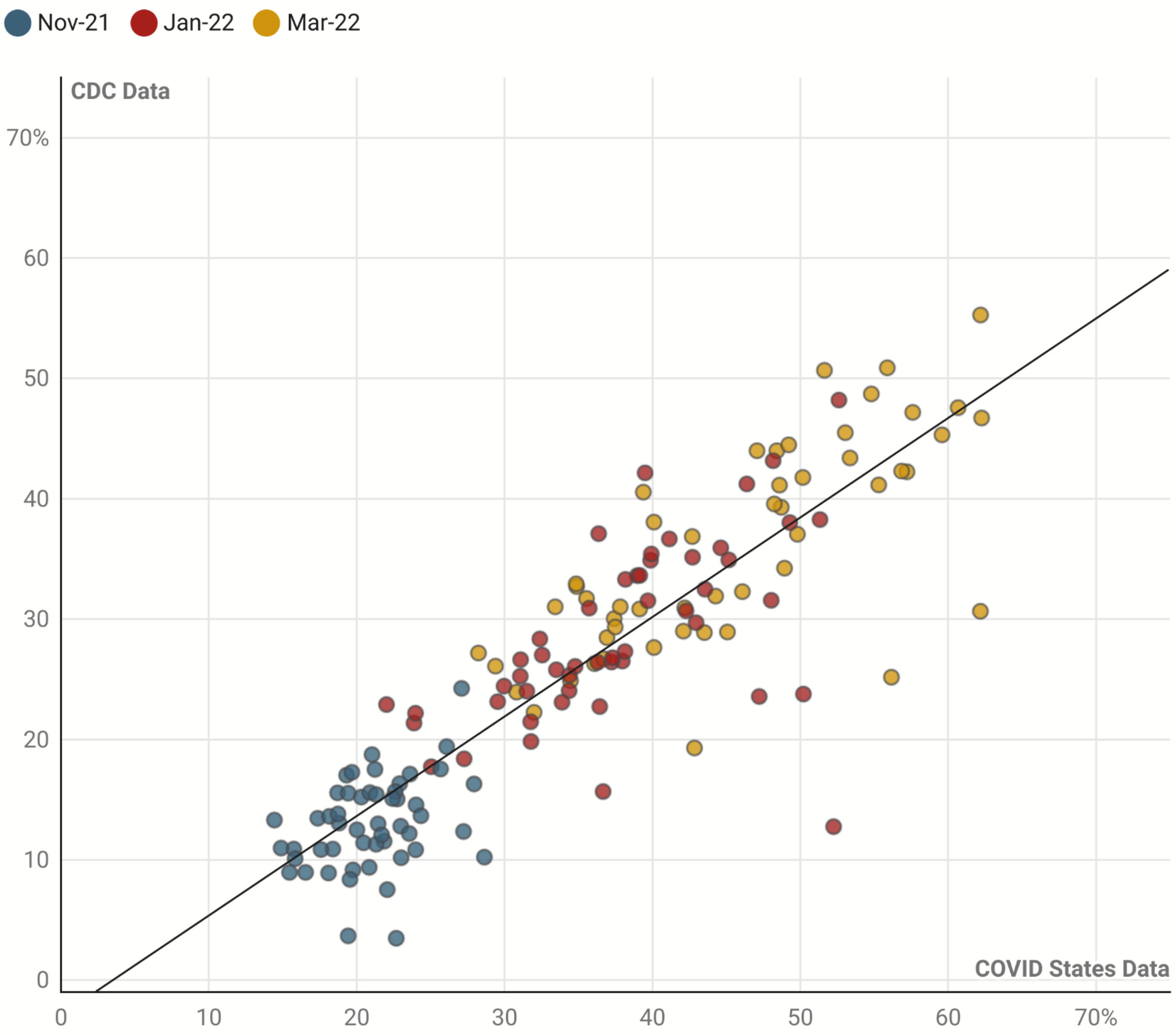

Nov-21: N = 22,277, Time period: 11/03/2021-12/02/2021; Jan-22: N = 22,961, Time period: 12/22/2021-01/24/2022; Mar-22: N = 22,234, Time period: 03/02/2022-04/04/2022

Source: CDC, COVID States Project • Created with Datawrapper

Figure F7.

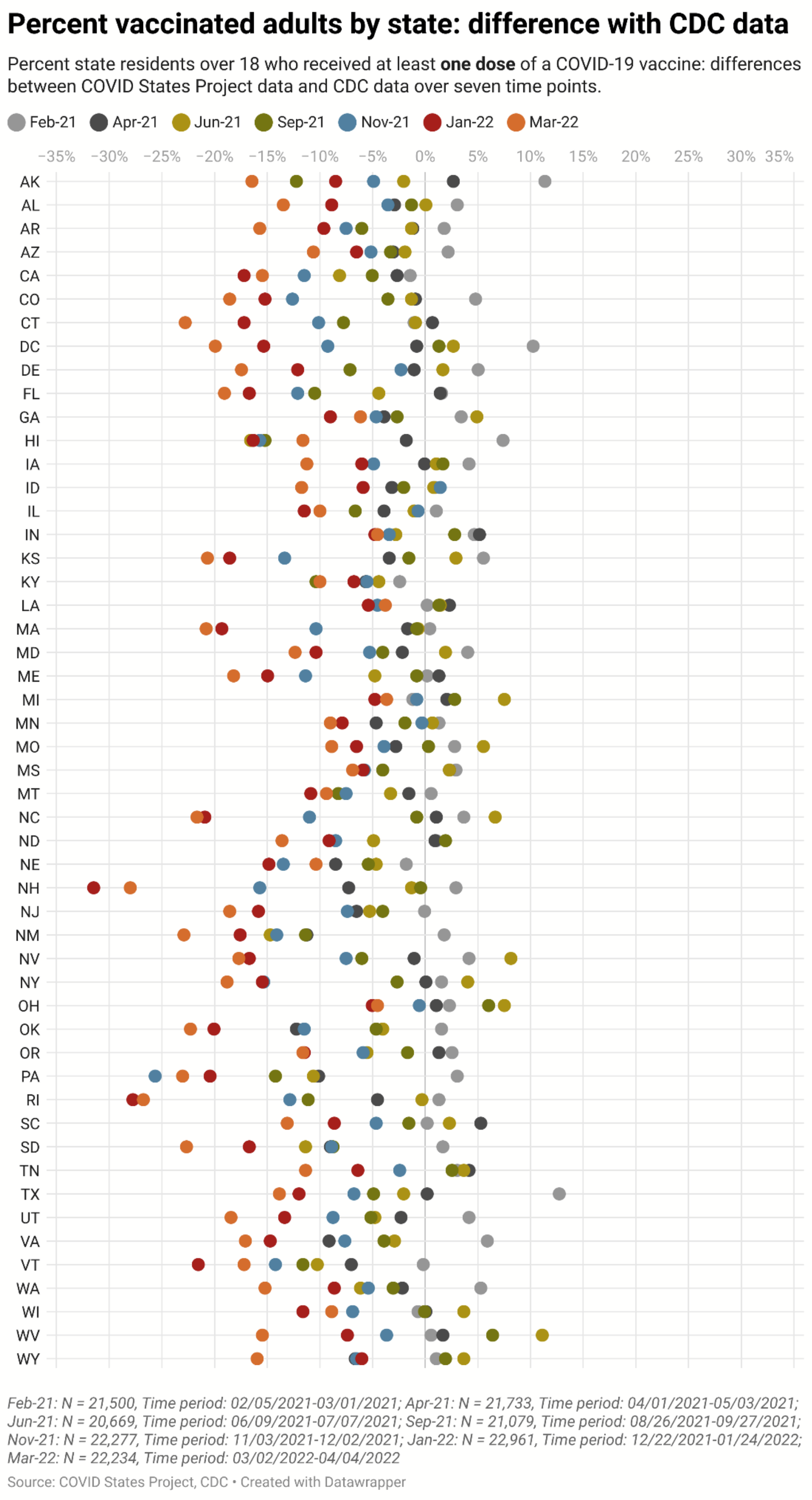

Percent vaccinated adults by state: difference with CDC data
Percent state residents over 18 who received at least **one dose** of a COVID-19 vaccine: differences between COVID States Project data and CDC data over seven time points.
Feb-21
Apr-21
Jun-21
Sep-21
Nov-21
Jan-22
Mar-22
−35%
−30%
−25%
−20%
−15%
−10%
−5%
0%
5%
10%
15%
20%
25%
30%
35%
AK
AL
AR
AZ
CA
CO
CT
DC
DE
FL
GA
HI
IA
ID
IL
IN
KS
KY
LA
MA
MD
ME
MI
MN
MO
MS
MT
NC
ND
NE
NH
NJ
NM
NV
NY
OH
OK
OR
PA
RI
SC
SD
TN
TX
UT
VA
VT
WA
WI
WV
WY
Feb-21: N = 21,500, Time period: 02/05/2021-03/01/2021; Apr-21: N = 21,733, Time period: 04/01/2021-05/03/2021; Jun-21: N = 20,669, Time period: 06/09/2021-07/07/2021; Sep-21: N = 21,079, Time period: 08/26/2021-09/27/2021; Nov-21: N = 22,277, Time period: 11/03/2021-12/02/2021; Jan-22: N = 22,961, Time period: 12/22/2021-01/24/2022; Mar-22: N = 22,234, Time period: 03/02/2022-04/04/2022
Source: COVID States Project, CDC • Created with Datawrapper

Figure F8.

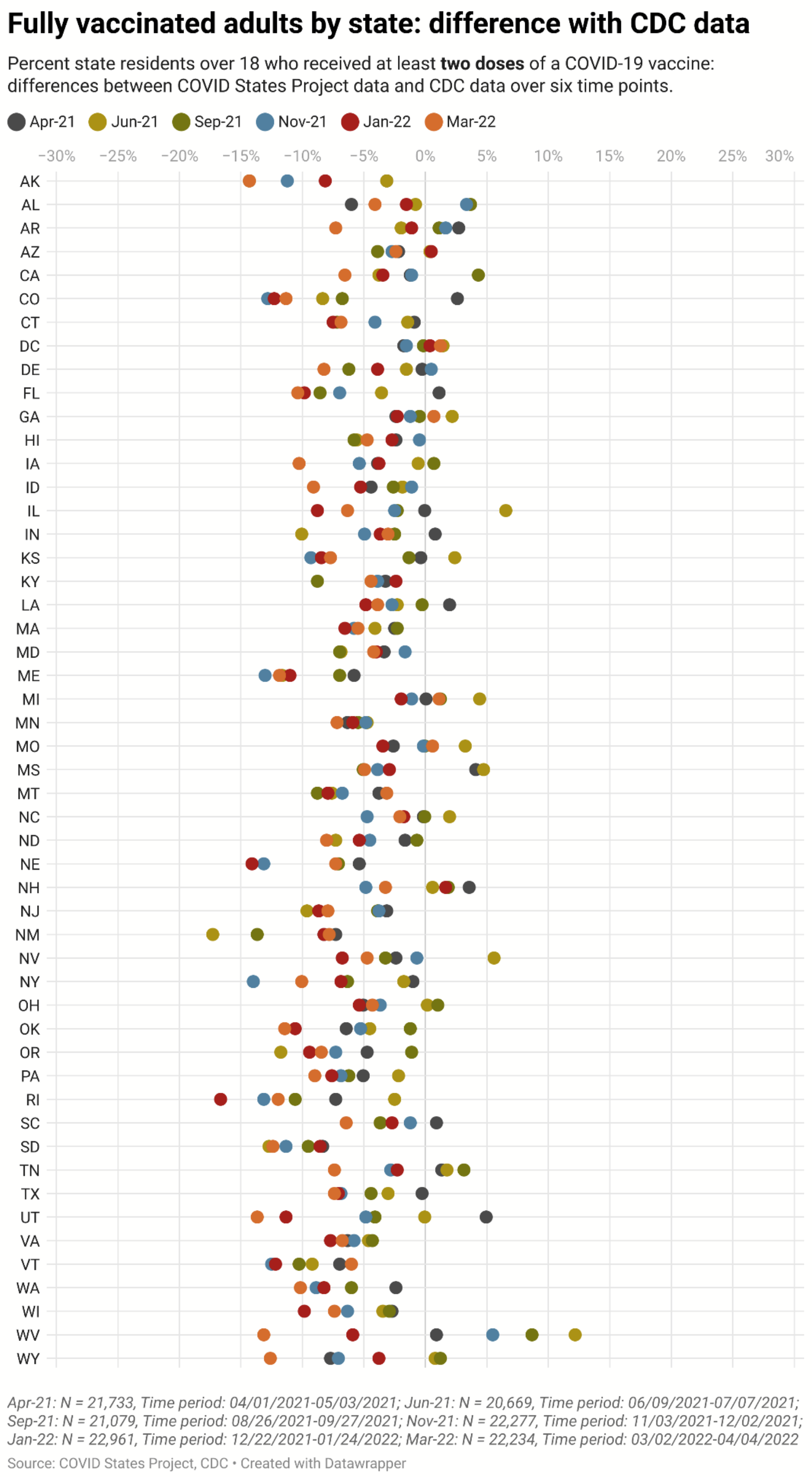
Fully vaccinated adults by state: difference with CDC data
Percent state residents over 18 who received at least **two doses** of a COVID-19 vaccine: differences between COVID States Project data and CDC data over six time points.
Apr-21
Jun-21
Sep-21
Nov-21
Jan-22
Mar-22
−30%
−25%
−20%
−15%
−10%
−5%
0%
5%
10%
15%
20%
25%
30%
AK
AL
AR
AZ
CA
CO
CT
DC
DE
FL
GA
HI
IA
ID
IL
IN
KS
KY
LA
MA
MD
ME
MI
MN
MO
MS
MT
NC
ND
NE
NH
NJ
NM
NV
NY
OH
OK
OR
PA
RI
SC
SD
TN
TX
UT
VA
VT
WA
WI
WV
WY
Apr-21: N = 21,733, Time period: 04/01/2021-05/03/2021; Jun-21: N = 20,669, Time period: 06/09/2021-07/07/2021; Sep-21: N = 21,079, Time period: 08/26/2021-09/27/2021; Nov-21: N = 22,277, Time period: 11/03/2021-12/02/2021; Jan-22: N = 22,961, Time period: 12/22/2021-01/24/2022; Mar-22: N = 22,234, Time period: 03/02/2022-04/04/2022
Source: COVID States Project, CDC • Created with Datawrapper

Figure F9.

Percent boosted adults by state: difference with CDC data

Percent state residents over 18 who received at least **three doses** of a COVID-19 vaccine: differences between COVID States Project data and CDC data over three time points.

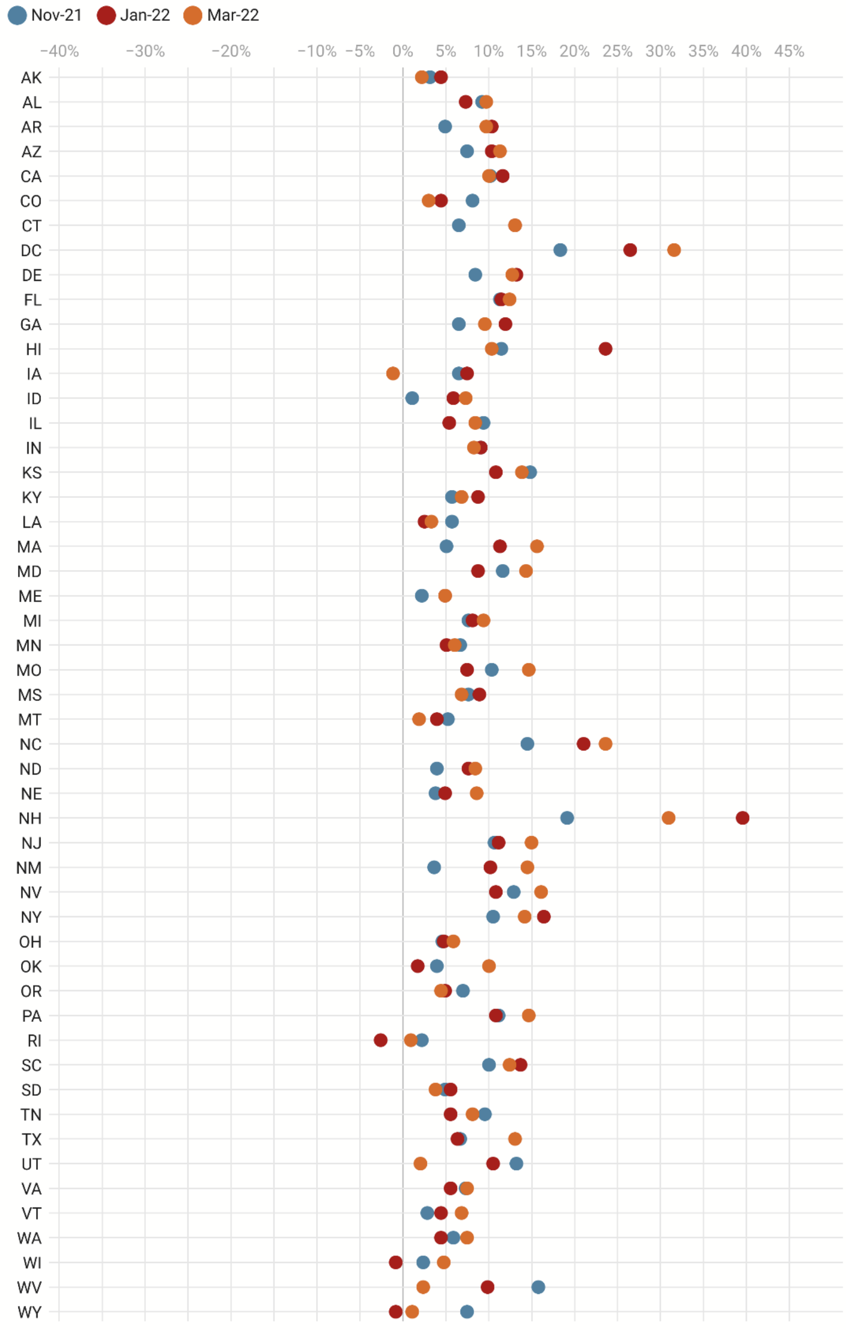

Nov-21: N = 22,277, Time period: 11/03/2021-12/02/2021; Jan-22: N = 22,961, Time period: 12/22/2021-01/24/2022
Mar-22: N = 22,234, Time period: 03/02/2022-04/04/2022

Source: COVID States Project, CDC • Created with Datawrapper

Figure F10.

Reference

Bradley, V. C., Kuriwaki, S., Isakov, M., Sejdinovic, D., Meng, X.-L., & Flaxman, S. (2021). Unrepresentative big surveys significantly overestimated US vaccine uptake. *Nature*, *600*(7890), 695–700. https://doi.org/10.1038/s41586-021-04198-4
