# Supplementary material for: Media use and vaccine resistance
Source: PNAS Nexus. 2023 May 9;2(5):pgad146. doi: 10.1093/pnasnexus/pgad146 (PMC10178922; doi:10.1093/pnasnexus/pgad146)
Supplement: pgad146_Supplementary_Data [file pgad146_supplementary_data.zip › PNASNEXUS-PNASNEXUS-2022-00931-s02.pdf]

Appendix D: Additional Tables and Figures

Figure D1: Vaccine Resistance by News Type, Wave, and Party ID

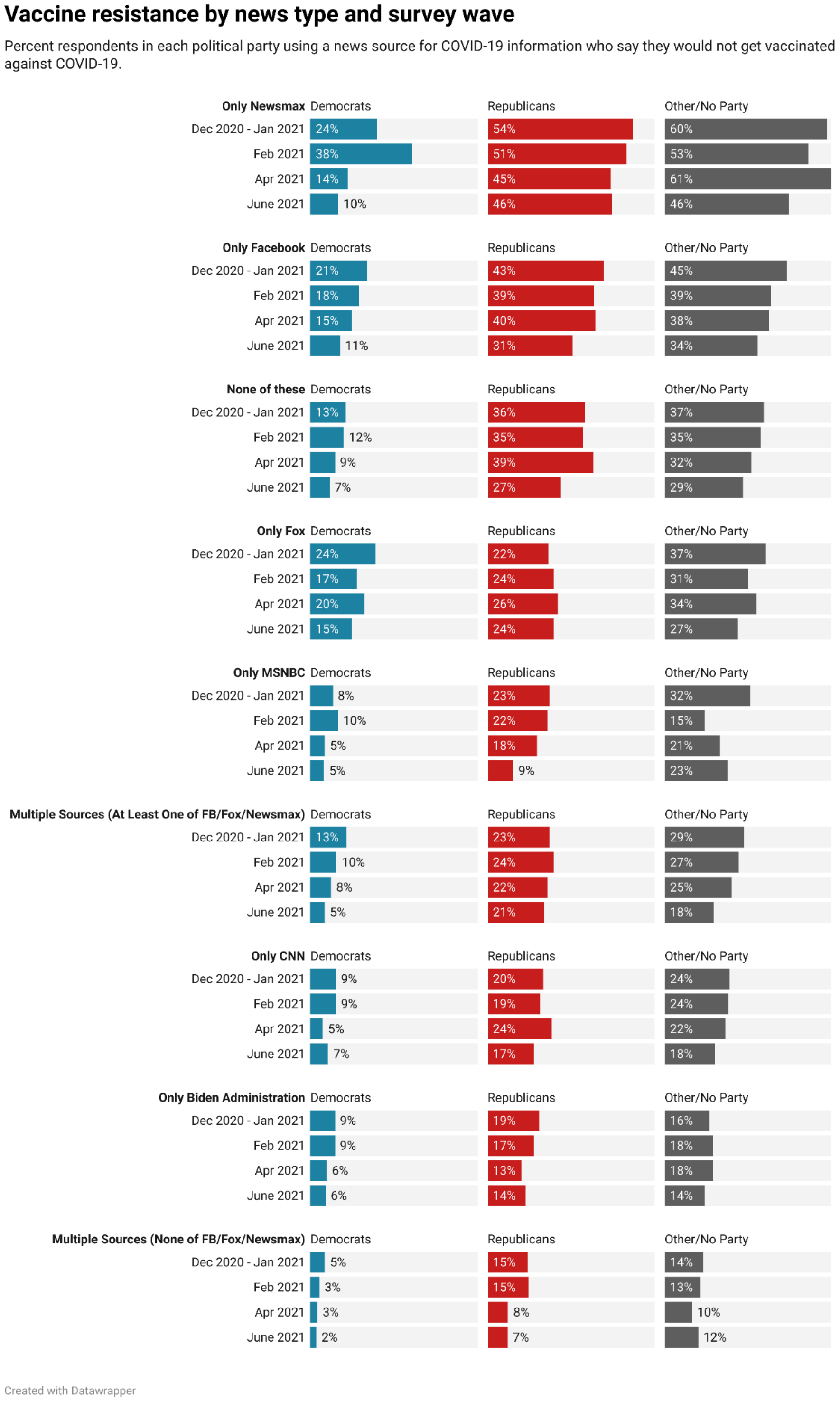

a)

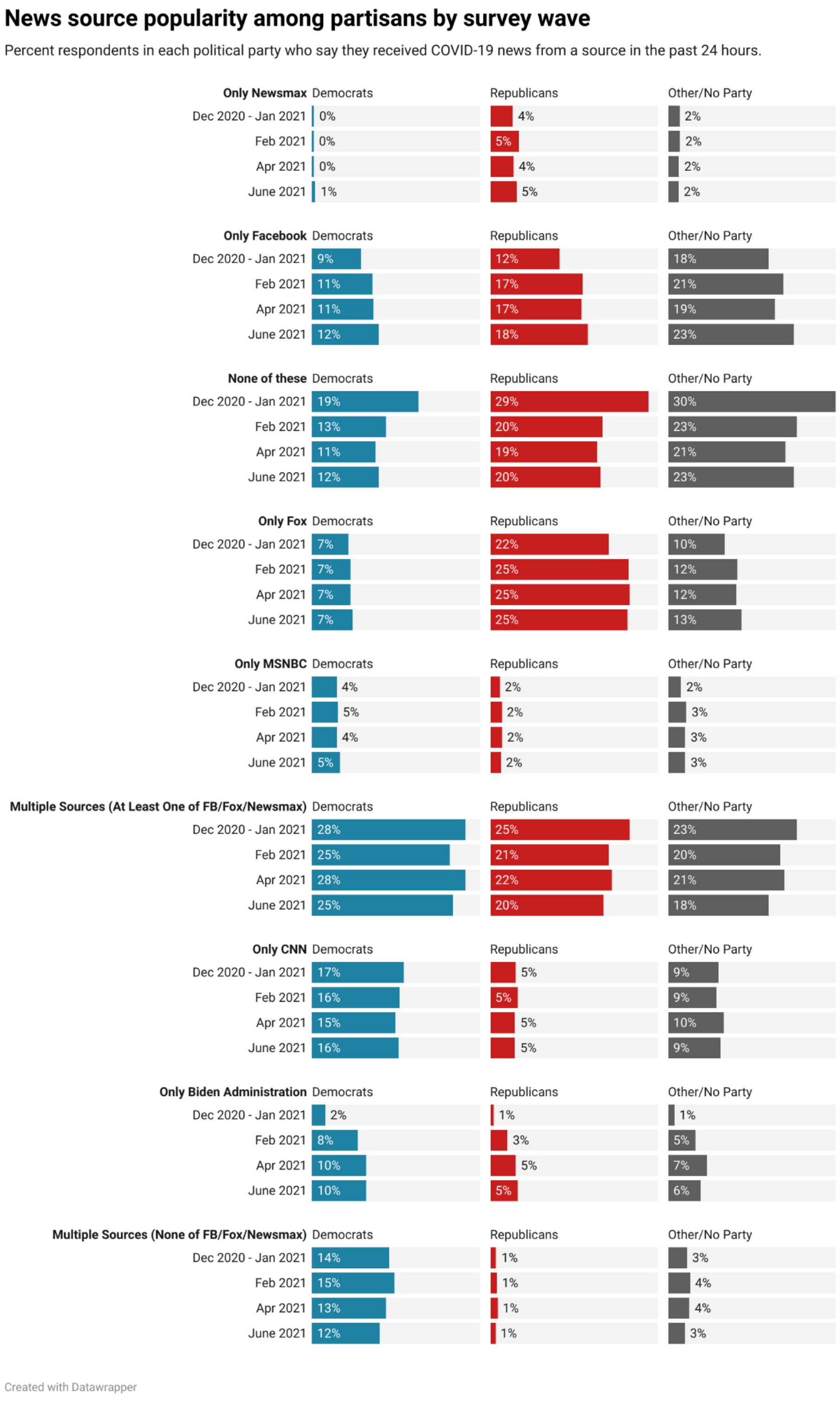

b)

Note: This figure replicates Figure 1 in the main manuscript, broken out by party ID. Proportion using source appears in a separate panel (as opposed to on the right hand side of the figure as reported in Figure 1 of the manuscript) for clarity.

Figure D2: Vaccination by Social Media Use and Age Group (August-September 2021)

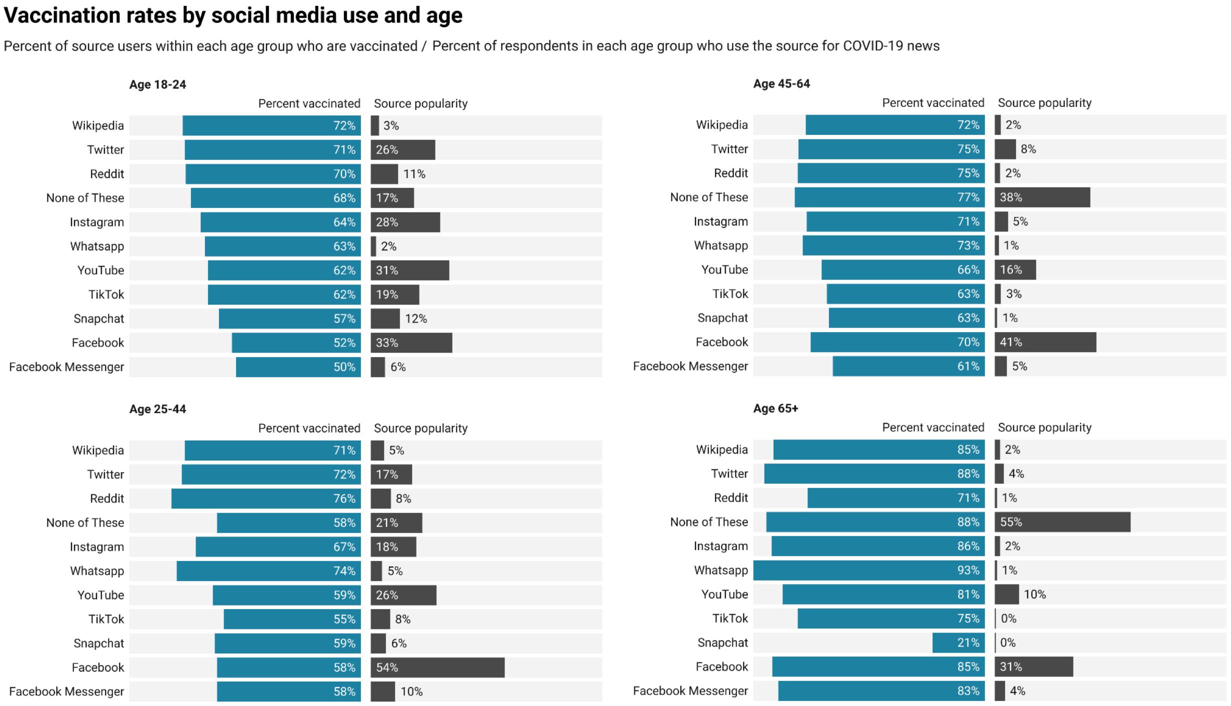

a)

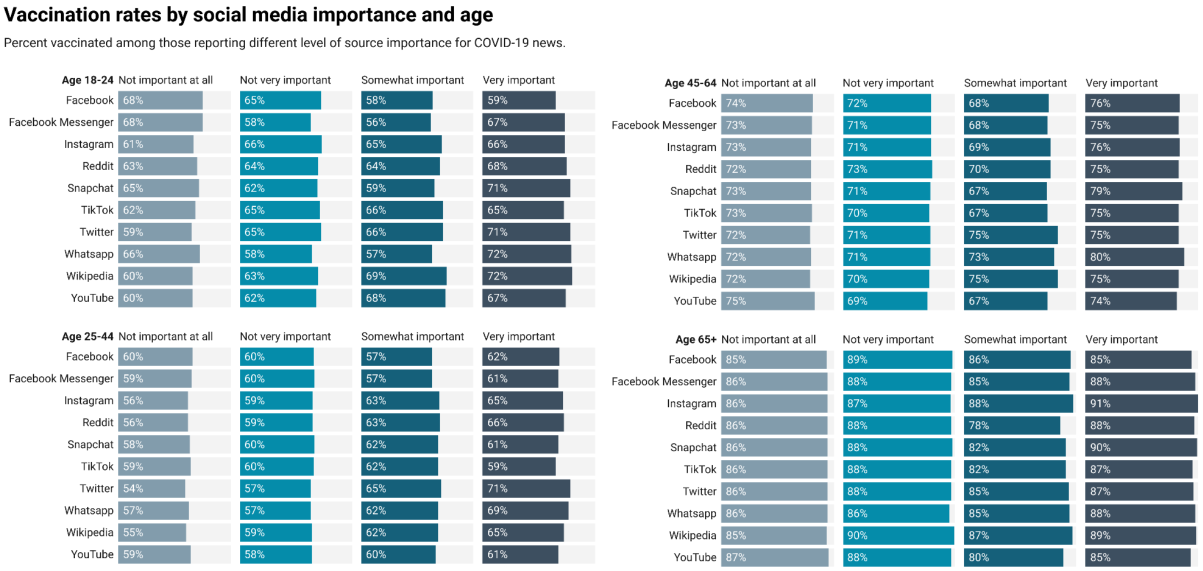

b)

Note: These figures replicate Figures 4a and 4b in the main manuscript, broken out by age group.

Figure D3: How Facebook Users Report Seeing COVID-19 News/Information

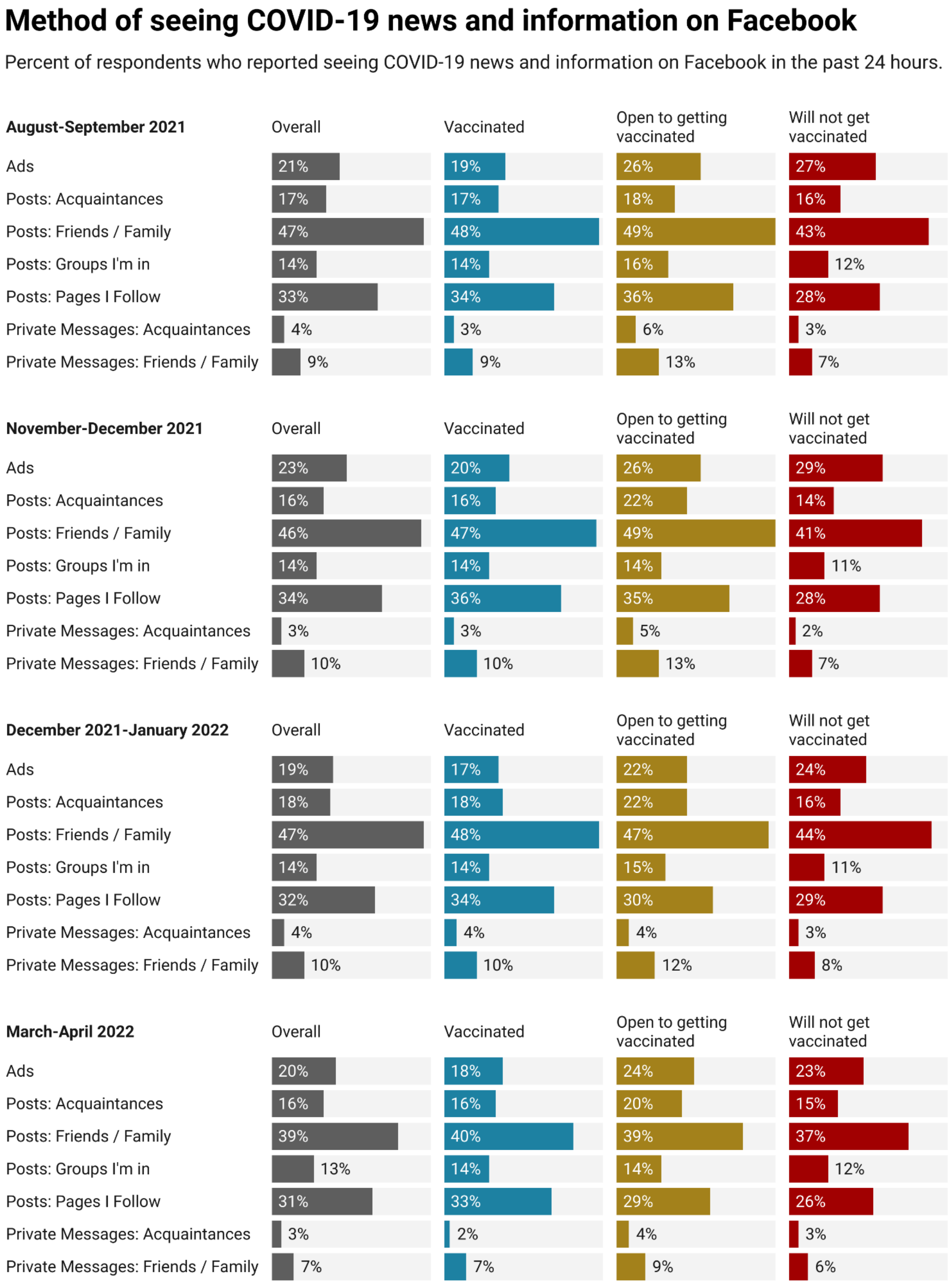

Figure D4: Vaccine Sentiment of COVID-19 News/Information Seen on Facebook by Vaccination Status

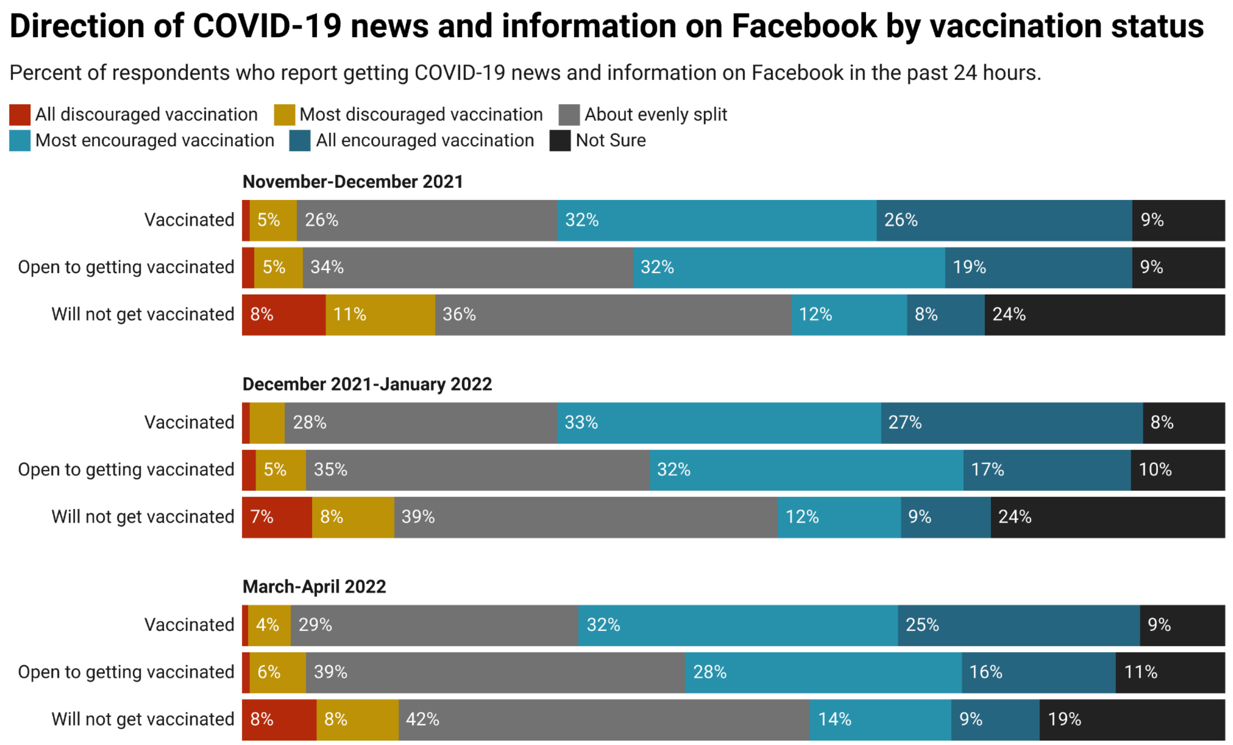

Figure D5: How People Consume Sources for COVID-19 News/Information (March-April 2022)

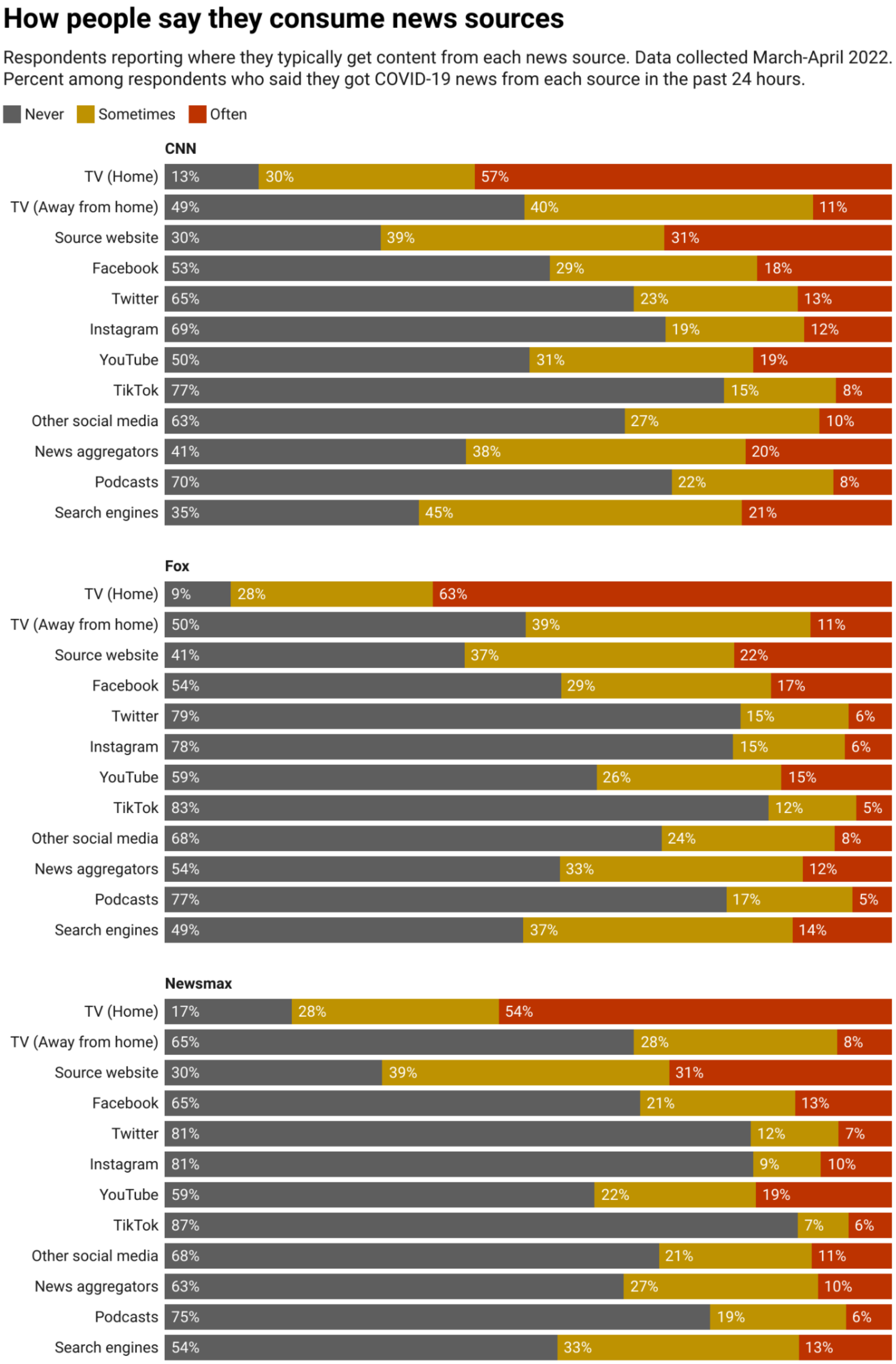

Table D1: News Type Consistency: Initial (December 2020) to Most Recent (As Late As June-July 2021) Response

News Type	Prop. of Those in Type in First Response in Type in Most Recent Response
Multiple Sources, None of FB/Fox/Newsmax	0.484
Only Biden Administration	0.470
Only CNN	0.475
Only MSNBC	0.424
Only Fox	0.604
Only Facebook	0.525
Only Newsmax	0.394
Multiple Sources, At Least One of FB/Fox/Newsmax	0.515
None of these	0.496

Table D2: News Source Consistency: Initial (December 2020) to Most Recent (As Late As June-July 2021) Response

News Source	Prop. Selecting in Most Recent Response After Selecting in First Response
CNN	0.851
Fox News	0.838

MSNBC	0.888
Biden	0.800
Facebook	0.820
Newsmax	0.947
None of these	0.496
