# Supplementary material for: Media use and vaccine resistance
Source: PNAS Nexus. 2023 May 9;2(5):pgad146. doi: 10.1093/pnasnexus/pgad146 (PMC10178922; doi:10.1093/pnasnexus/pgad146)
Supplement: pgad146_Supplementary_Data [file pgad146_supplementary_data.zip › PNASNEXUS-PNASNEXUS-2022-00931-s03.pdf]

Appendix B: Regression Tables

Table B1: Logistic Regression, Vaccine Resistance in December 2020-January 2021 Wave (Between Subjects)

	Model 1	Model 2	Model 3	Model 4
News Type: Only Biden Administration	0.660***	0.631***	0.608***	0.497**
	(0.187)	(0.192)	(0.193)	(0.196)
News Type: Only CNN	0.721***	0.619***	0.603***	0.427***
	(0.115)	(0.117)	(0.117)	(0.120)
News Type: Only MSNBC	0.834***	0.773***	0.732***	0.539***
	(0.142)	(0.146)	(0.146)	(0.150)
News Type: Only Fox	1.629***	1.510***	1.487***	0.852***
	(0.108)	(0.110)	(0.110)	(0.115)
News Type: Only Facebook	2.020***	1.715***	1.668***	1.222***
	(0.105)	(0.108)	(0.108)	(0.112)
News Type: Only Newsmax	2.791***	2.898***	2.854***	2.126***

	(0.136)	(0.142)	(0.142)	(0.147)
News Type: Multiple Sources, At Least One of FB/Fox/Newsmax	1.281***	1.153***	1.130***	0.747***
	(0.103)	(0.106)	(0.106)	(0.109)
News Type: None of these	1.795***	1.707***	1.681***	1.265***
	(0.102)	(0.105)	(0.105)	(0.108)
Age Group: 25-44		0.439***	0.439***	0.436***
		(0.052)	(0.052)	(0.053)
Age Group: 45-64		0.264***	0.260***	0.184***
		(0.053)	(0.053)	(0.055)
Age Group: 65+		−0.334***	−0.340***	−0.480***
		(0.065)	(0.065)	(0.067)
Race: Latino		−0.175**	−0.111	0.045
		(0.068)	(0.069)	(0.071)

Race: Black	0.618***	0.662***	1.136***
	(0.049)	(0.051)	(0.055)
Race: Asian	−0.762***	−0.706***	−0.586***
	(0.100)	(0.101)	(0.103)
Race: Other Race	0.334***	0.337***	0.478***
	(0.076)	(0.077)	(0.078)
Female	0.315***	0.309***	0.380***
	(0.035)	(0.035)	(0.036)
College	−0.830***	−0.803***	−0.756***
	(0.039)	(0.039)	(0.040)
Children in Household	0.181***	0.166***	0.127***
	(0.037)	(0.037)	(0.038)
Income (9pt)	−0.729***	−0.681***	−0.804***
	(0.062)	(0.062)	(0.064)

Census Region: Midwest			0.165***	0.114**
			(0.051)	(0.053)
Census Region: South			0.258***	0.158***
			(0.049)	(0.050)
Census Region: West			0.200***	0.145***
			(0.051)	(0.053)
Suburban			−0.286***	−0.235***
			(0.038)	(0.039)
Urban			−0.429***	−0.291***
			(0.051)	(0.052)
Party ID				1.896***
				(0.062)
Num.Obs.	25640	25640	25640	25494
AIC	26670.6	24856.0	24751.4	23589.5

BIC	26744.0	25019.1	24955.2	23801.3
Log.Lik.	−13326.306	−12408.018	−12350.689	−11768.760

* p < 0.1, ** p < 0.05, *** p < 0.01

Table B2: Logistic Regression, Vaccine Resistance in February 2021 Wave (Between Subjects)

	Model 1	Model 2	Model 3	Model 4
News Type: Only Biden Administration	1.053***	1.005***	1.015***	0.799***
	(0.149)	(0.153)	(0.153)	(0.156)
News Type: Only CNN	0.973***	0.925***	0.920***	0.702***
	(0.139)	(0.142)	(0.143)	(0.145)
News Type: Only MSNBC	1.071***	1.033***	1.019***	0.773***
	(0.165)	(0.169)	(0.170)	(0.173)
News Type: Only Fox	1.917***	1.862***	1.848***	1.146***
	(0.129)	(0.133)	(0.133)	(0.137)

News Type: Only Facebook	2.250***	1.995***	1.958***	1.461***
	(0.127)	(0.130)	(0.130)	(0.134)
News Type: Only Newsmax	2.945***	3.120***	3.096***	2.295***
	(0.152)	(0.159)	(0.159)	(0.164)
News Type: Multiple Sources, At Least One of FB/Fox/Newsmax	1.396***	1.285***	1.271***	0.833***
	(0.128)	(0.131)	(0.131)	(0.134)
News Type: None of these	2.040***	1.993***	1.971***	1.481***
	(0.126)	(0.129)	(0.130)	(0.133)
Age Group: 25-44		0.333***	0.345***	0.340***
		(0.067)	(0.067)	(0.069)
Age Group: 45-64		0.081	0.080	0.010
		(0.067)	(0.067)	(0.069)
Age Group: 65+		−0.607***	−0.608***	−0.734***

	(0.078)	(0.078)	(0.080)
Race: Latino	−0.044	0.041	0.226***
	(0.081)	(0.082)	(0.084)
Race: Black	0.307***	0.381***	0.842***
	(0.063)	(0.065)	(0.069)
Race: Asian	−0.584***	−0.524***	−0.385***
	(0.113)	(0.114)	(0.116)
Race: Other Race	0.308***	0.293***	0.435***
	(0.099)	(0.100)	(0.102)
Female	0.343***	0.332***	0.393***
	(0.041)	(0.041)	(0.042)
College	−0.830***	−0.802***	−0.725***
	(0.043)	(0.043)	(0.044)
Children in Household	0.142***	0.123***	0.118***

	(0.044)	(0.044)	(0.045)
Income (9pt)	−1.078***	−1.014***	−1.207***
	(0.082)	(0.083)	(0.086)
Census Region: Midwest		0.314***	0.247***
		(0.060)	(0.062)
Census Region: South		0.370***	0.269***
		(0.057)	(0.058)
Census Region: West		0.380***	0.334***
		(0.060)	(0.062)
Suburban		−0.290***	−0.238***
		(0.044)	(0.045)
Urban		−0.562***	−0.433***
		(0.059)	(0.061)
Party ID			1.992***

				(0.073)
Num.Obs.	21500	21306	21306	21164
AIC	20943.9	19273.3	19130.8	18193.3
BIC	21015.6	19432.6	19329.9	18400.3
Log.Lik.	−10462.931	−9616.631	−9540.389	−9070.652

* p < 0.1, ** p < 0.05, *** p < 0.01

Table B3: Logistic Regression, Vaccine Resistance in April 2021 Wave (Between Subjects)

	Model 1	Model 2	Model 3	Model 4
News Type: Only Biden Administration	1.133***	0.993***	0.993***	0.718***
	(0.177)	(0.179)	(0.180)	(0.183)
News Type: Only CNN	1.207***	1.038***	1.042***	0.791***
	(0.169)	(0.171)	(0.172)	(0.174)
News Type: Only MSNBC	1.350***	1.163***	1.139***	0.823***

	(0.194)	(0.198)	(0.198)	(0.202)
News Type: Only Fox	2.414***	2.243***	2.240***	1.434***
	(0.158)	(0.160)	(0.160)	(0.164)
News Type: Only Facebook	2.524***	2.169***	2.133***	1.555***
	(0.156)	(0.159)	(0.159)	(0.162)
News Type: Only Newsmax	3.439***	3.327***	3.302***	2.398***
	(0.183)	(0.188)	(0.189)	(0.194)
News Type: Multiple Sources, At Least One of FB/Fox/Newsmax	1.569***	1.518***	1.509***	1.016***
	(0.157)	(0.159)	(0.159)	(0.162)
News Type: None of these	2.409***	2.203***	2.177***	1.621***
	(0.156)	(0.158)	(0.158)	(0.161)
Age Group: 25-44		0.438***	0.441***	0.423***
		(0.071)	(0.071)	(0.073)

Age Group: 45-64	0.227***	0.209***	0.053
	(0.070)	(0.070)	(0.073)
Age Group: 65+	−0.382***	−0.390***	−0.627***
	(0.079)	(0.080)	(0.083)
Race: Latino	−0.276***	−0.171*	0.036
	(0.088)	(0.089)	(0.092)
Race: Black	0.246***	0.336***	0.859***
	(0.067)	(0.069)	(0.074)
Race: Asian	−0.725***	−0.634***	−0.560***
	(0.122)	(0.123)	(0.126)
Race: Other Race	0.212**	0.222**	0.364***
	(0.106)	(0.107)	(0.109)
Female	0.299***	0.274***	0.306***
	(0.044)	(0.044)	(0.045)

College	−0.789***	−0.761***	−0.646***
	(0.047)	(0.047)	(0.048)
Children in Household	0.149***	0.119**	0.145***
	(0.048)	(0.048)	(0.050)
Income (9pt)	−1.314***	−1.226***	−1.402***
	(0.091)	(0.092)	(0.095)
Census Region: Midwest		0.364***	0.286***
		(0.064)	(0.066)
Census Region: South		0.411***	0.295***
		(0.060)	(0.062)
Census Region: West		0.363***	0.260***
		(0.066)	(0.068)
Suburban		−0.262***	−0.200***
		(0.048)	(0.050)

Urban			−0.615***	−0.457***
			(0.063)	(0.066)
Party ID				2.335***
				(0.077)
Num.Obs.	21733	21328	21328	21211
AIC	18686.0	17158.2	17013.9	15932.6
BIC	18757.9	17317.5	17213.1	16139.6
Log.Lik.	−9334.017	−8559.097	−8481.956	−7940.279

* p < 0.1, ** p < 0.05, *** p < 0.01

Table B4: Logistic Regression, Vaccine Resistance in June-July 2021 Wave (Between Subjects)

	**Model 1**	**Model 2**	**Model 3**	**Model 4**
News Type: Only Biden Administration	0.849***	0.694***	0.667***	0.355*
	(0.183)	(0.185)	(0.186)	(0.189)

News Type: Only CNN	1.158***	1.004***	1.012***	0.750***
	(0.170)	(0.171)	(0.172)	(0.174)
News Type: Only MSNBC	0.917***	0.729***	0.744***	0.457**
	(0.206)	(0.208)	(0.209)	(0.212)
News Type: Only Fox	2.102***	1.878***	1.846***	1.045***
	(0.161)	(0.163)	(0.163)	(0.167)
News Type: Only Facebook	2.223***	1.917***	1.880***	1.300***
	(0.158)	(0.161)	(0.161)	(0.164)
News Type: Only Newsmax	2.926***	2.787***	2.729***	1.822***
	(0.185)	(0.188)	(0.189)	(0.194)
News Type: Multiple Sources, At Least One of FB/Fox/Newsmax	1.264***	1.224***	1.195***	0.685***
	(0.160)	(0.162)	(0.163)	(0.166)
News Type: None of these	2.029***	1.866***	1.854***	1.292***

	(0.159)	(0.160)	(0.161)	(0.164)
Age Group: 25-44		0.368***	0.363***	0.346***
		(0.068)	(0.069)	(0.070)
Age Group: 45-64		0.277***	0.264***	0.162**
		(0.069)	(0.070)	(0.072)
Age Group: 65+		−0.155*	−0.162**	−0.335***
		(0.079)	(0.080)	(0.082)
Race: Latino		−0.059	−0.031	0.172**
		(0.077)	(0.079)	(0.082)
Race: Black		0.252***	0.176***	0.688***
		(0.063)	(0.066)	(0.070)
Race: Asian		−0.999***	−0.906***	−0.768***
		(0.145)	(0.146)	(0.149)
Race: Other Race		−0.011	0.015	0.163

	(0.120)	(0.120)	(0.123)
Female	0.269***	0.255***	0.272***
	(0.048)	(0.048)	(0.049)
College	−0.771***	−0.768***	−0.671***
	(0.052)	(0.052)	(0.053)
Children in Household	0.046	0.024	0.033
	(0.048)	(0.049)	(0.050)
Income (9pt)	−1.160***	−1.083***	−1.333***
	(0.097)	(0.098)	(0.101)
Census Region: Midwest		0.480***	0.419***
		(0.073)	(0.075)
Census Region: South		0.724***	0.617***
		(0.069)	(0.070)
Census Region: West		0.264***	0.157**

			(0.075)	(0.077)
Suburban			−0.172***	−0.090*
			(0.052)	(0.053)
Urban			−0.194***	−0.007
			(0.066)	(0.068)
Party ID				2.254***
				(0.082)
Num.Obs.	20669	20669	20669	20569
AIC	16725.3	15839.3	15688.1	14801.1
BIC	16796.7	15998.0	15886.6	15007.3
Log.Lik.	−8353.650	−7899.632	−7819.073	−7374.529

* p < 0.1, ** p < 0.05, *** p < 0.01

Table B5: Logistic Regression, Vaccine Resistance Pooled Across Waves (Between Subjects)

	Model 1	Model 2	Model 3	Model 4

February 2021 Wave	−0.113***	−0.091***	−0.088***	−0.090***
	(0.019)	(0.020)	(0.020)	(0.021)
April 2021 Wave	−0.288***	−0.216***	−0.210***	−0.190***
	(0.020)	(0.022)	(0.022)	(0.022)
June-July 2021 Wave	−0.500***	−0.502***	−0.502***	−0.501***
	(0.023)	(0.024)	(0.024)	(0.025)
News Type: Only Biden Administration	0.877***	0.793***	0.788***	0.566***
	(0.066)	(0.069)	(0.070)	(0.075)
News Type: Only CNN	0.844***	0.758***	0.751***	0.573***
	(0.060)	(0.062)	(0.063)	(0.067)
News Type: Only MSNBC	0.940***	0.850***	0.830***	0.608***
	(0.071)	(0.074)	(0.075)	(0.080)
News Type: Only Fox	1.741***	1.606***	1.591***	0.985***
	(0.058)	(0.060)	(0.061)	(0.065)

News Type: Only Facebook	1.968***	1.698***	1.661***	1.231***
	(0.056)	(0.059)	(0.059)	(0.063)
News Type: Only Newsmax	2.633***	2.604***	2.574***	1.896***
	(0.072)	(0.077)	(0.077)	(0.081)
News Type: Multiple Sources, At Least One of FB/Fox/Newsmax	1.179***	1.111***	1.095***	0.724***
	(0.056)	(0.058)	(0.059)	(0.062)
News Type: None of these	1.802***	1.681***	1.660***	1.244***
	(0.055)	(0.058)	(0.058)	(0.062)
Age Group: 25-44		0.399***	0.400***	0.392***
		(0.032)	(0.033)	(0.033)
Age Group: 45-64		0.221***	0.213***	0.120***
		(0.033)	(0.033)	(0.034)
Age Group: 65+		−0.368***	−0.374***	−0.530***
		(0.040)	(0.040)	(0.041)

Race: Latino	−0.142***	−0.062	0.122***
	(0.040)	(0.041)	(0.042)
Race: Black	0.368***	0.408***	0.888***
	(0.031)	(0.032)	(0.034)
Race: Asian	−0.785***	−0.703***	−0.579***
	(0.062)	(0.063)	(0.063)
Race: Other Race	0.198***	0.206***	0.345***
	(0.052)	(0.052)	(0.054)
Female	0.321***	0.308***	0.355***
	(0.022)	(0.022)	(0.023)
College	−0.845***	−0.817***	−0.736***
	(0.024)	(0.024)	(0.024)
Children in Household	0.126***	0.105***	0.095***
	(0.023)	(0.023)	(0.023)

Income (9pt)	−0.937***	−0.871***	−1.019***
	(0.042)	(0.042)	(0.043)
Census Region: Midwest		0.308***	0.247***
		(0.032)	(0.033)
Census Region: South		0.409***	0.304***
		(0.030)	(0.031)
Census Region: West		0.284***	0.210***
		(0.033)	(0.033)
Suburban		−0.266***	−0.208***
		(0.024)	(0.024)
Urban		−0.476***	−0.327***
		(0.031)	(0.032)
Party ID			2.072***
			(0.036)

Num.Obs.	88438	88438	88438	88438
AIC	82013.72	76318.33	75693.27	71928.13
BIC	82137.79	76545.69	75967.59	72211.83
Log.Lik.	-40994.86	-38136.17	-37818.64	-35935.06

* p < 0.1, ** p < 0.05, *** p < 0.01

Generalized estimating equation corrects for correlations within returning respondents

Table B6: Logistic Regression, Subsequent Vaccination by Previous Winter News Type (Within-Subjects)

Note: PureSpectrum, the survey vendor used to collect these data, subcontracts from multiple vendors and uses a proprietary record linkage algorithm to identify the same person across multiple vendors, assigning them a common identifying number in their system. This record linkage is useful, but not perfect. Occasionally we have identified respondents with different demographic characteristics assigned to the same PureSpectrum ID. As such, we apply two different filters for this model. Both remove respondents who said they were vaccinated and in any subsequent wave said they were not vaccinated. The first (Basic Filter in the columns below) removes respondents who report ages in their first and most recent response that are more than two years apart (accommodating the possibility that they signed up for different survey panels at slightly different times) *and* who are identified as living in different ZIP codes between those two responses. The second, stricter filter (Strict Filter) removes respondents who are mismatched on *either* of these characteristics. In addition to these filters, we also control for the baseline probability of being vaccinated increasing over time in two ways: first, by including a categorical variable for the wave in which the most recent response was recorded; second, by adjusting for the national adult vaccination rate (according to the CDC) on the day the most recent response was recorded. Figure 3 in the manuscript uses coefficients from the first model reported below (Basic Filter, wave of most recent response used to account for baseline probability).

	Basic Filter	Strict Filter	Basic Filter	Strict Filter

Initial Vaccine Stance: Won't Get Vaccinated	−2.633***	−2.733***	−2.734***	−2.842***
	(0.111)	(0.117)	(0.114)	(0.121)
Most Recent Wave: April 2021	2.394***	2.417***		
	(0.090)	(0.093)		
Most Recent Wave: June-July 2021	3.297***	3.325***		
	(0.096)	(0.100)		
US Adult Vaccination Rate at Most Recent Response			0.059***	0.060***
			(0.002)	(0.002)
News Type: Only Biden Administration	−0.282	−0.262	−0.317	−0.291
	(0.256)	(0.261)	(0.252)	(0.257)
News Type: Only CNN	−0.266*	−0.235	−0.268*	−0.235
	(0.161)	(0.167)	(0.158)	(0.164)
News Type: Only MSNBC	−0.061	−0.038	−0.089	−0.070

	(0.206)	(0.211)	(0.202)	(0.207)
News Type: Only Fox	−0.468***	−0.456***	−0.447***	−0.427**
	(0.167)	(0.174)	(0.165)	(0.171)
News Type: Only Facebook	−0.375**	−0.319*	−0.388**	−0.337*
	(0.173)	(0.180)	(0.171)	(0.178)
News Type: Only Newsmax	0.023	0.120	0.048	0.147
	(0.281)	(0.290)	(0.273)	(0.282)
News Type: Multiple Sources, At Least One of FB/Fox/Newsmax	−0.229	−0.155	−0.245	−0.169
	(0.157)	(0.163)	(0.154)	(0.160)
News Type: None of these	−0.391***	−0.390***	−0.389***	−0.383***
	(0.145)	(0.150)	(0.142)	(0.147)
Age Group: 25-44	0.034	0.213	0.051	0.207
	(0.218)	(0.244)	(0.221)	(0.247)

Age Group: 45-64	0.729***	0.944***	0.764***	0.954***
	(0.212)	(0.237)	(0.214)	(0.240)
Age Group: 65+	1.596***	1.819***	1.608***	1.804***
	(0.214)	(0.239)	(0.216)	(0.242)
Race: Latino	−0.147	−0.118	−0.173	−0.140
	(0.207)	(0.217)	(0.206)	(0.217)
Race: Black	−0.172	−0.180	−0.227*	−0.237*
	(0.137)	(0.144)	(0.137)	(0.143)
Race: Asian	−0.049	−0.034	−0.001	0.023
	(0.167)	(0.174)	(0.164)	(0.171)
Race: Other Race	−0.076	−0.033	−0.008	0.031
	(0.229)	(0.239)	(0.227)	(0.236)
Female	−0.022	−0.023	−0.014	−0.016
	(0.074)	(0.076)	(0.073)	(0.075)

College	0.475***	0.461***	0.444***	0.432***
	(0.076)	(0.079)	(0.075)	(0.078)
Children in Household	−0.231**	−0.251**	−0.204**	−0.217**
	(0.102)	(0.107)	(0.101)	(0.107)
Income (9pt)	1.030***	1.055***	0.996***	1.006***
	(0.145)	(0.152)	(0.143)	(0.150)
Census Region: Midwest	0.209**	0.209*	0.205*	0.205*
	(0.106)	(0.111)	(0.105)	(0.109)
Census Region: South	0.080	0.081	0.077	0.080
	(0.102)	(0.106)	(0.101)	(0.105)
Census Region: West	0.058	0.077	0.070	0.086
	(0.107)	(0.111)	(0.106)	(0.110)
Suburban	−0.001	0.043	0.026	0.067
	(0.089)	(0.092)	(0.088)	(0.092)

Urban	0.068	0.107	0.098	0.138
	(0.114)	(0.118)	(0.112)	(0.117)
Party ID	−0.606***	−0.641***	−0.594***	−0.632***
	(0.127)	(0.132)	(0.126)	(0.131)
Num.Obs.	6084	5720	6084	5720
AIC	5259.6	4894.7	5317.6	4952.9
BIC	5454.3	5087.6	5505.6	5139.2
Log.Lik.	−2600.816	−2418.342	−2630.821	−2448.459

* p < 0.1, ** p < 0.05, *** p < 0.01

Table B7: Logistic Regression, Vaccine Resistance (January 2021, Kaiser Family Foundation)

	Model 1	Model 2	Model 3	Model 4
Use: Facebook	0.583***	0.414**	0.372*	0.311
	(0.194)	(0.201)	(0.204)	(0.207)

Use: Other Social Media	0.265	0.235	0.269	0.365
	(0.201)	(0.215)	(0.218)	(0.223)
Use: Fox, OANN, or Newsmax	0.708***	0.732***	0.719***	0.456**
	(0.192)	(0.199)	(0.201)	(0.208)
Use: MSNBC	−0.547**	−0.569**	−0.575**	−0.367
	(0.258)	(0.265)	(0.266)	(0.275)
Use: CNN or Other Media	−0.832***	−0.846***	−0.809***	−0.601**
	(0.220)	(0.228)	(0.230)	(0.240)
Use: No social media or cable	0.312	0.261	0.279	0.217
	(0.238)	(0.242)	(0.243)	(0.247)
Race: Black or African-American		1.658	1.755*	1.995*
		(1.039)	(1.043)	(1.044)
Race: Refused		0.731	0.777	0.658
		(1.189)	(1.195)	(1.198)

Female	0.341**	0.378**	0.451***
	(0.167)	(0.169)	(0.172)
College	−0.356*	−0.282	−0.199
	(0.191)	(0.192)	(0.194)
Age Group: 25-34	0.737*	0.782*	0.752*
	(0.396)	(0.401)	(0.406)
Age Group: 35-44	0.795**	0.716*	0.735*
	(0.396)	(0.400)	(0.406)
Age Group: 45-54	0.783**	0.714*	0.642
	(0.394)	(0.398)	(0.406)
Age Group: 55-64	0.568	0.465	0.336
	(0.397)	(0.402)	(0.410)
Age Group: 65+	−0.018	−0.086	−0.152
	(0.407)	(0.411)	(0.421)

Income: $90k+		−0.008	0.058	0.028
		(0.226)	(0.229)	(0.232)
Income: Don't Know / Refused		−0.372	−0.343	−0.351
		(0.339)	(0.342)	(0.346)
Income: Less than $40k		−0.034	0.046	0.147
		(0.207)	(0.210)	(0.213)
Suburban			−0.318	−0.277
			(0.232)	(0.235)
Urban			−1.059***	−0.892***
			(0.265)	(0.270)
Party ID				0.374***
				(0.067)
Num.Obs.	1562	1548	1548	1548
AIC	1090.6	1080.0	1063.0	1031.8

BIC	1128.1	1197.6	1191.2	1165.5
Log.Lik.	−538.298	−518.011	−507.475	−490.918
RMSE	0.32	0.31	0.31	0.31

Table B8: Logistic Regression, Vaccination by News Type (Real Clear Opinion Research)

	Model 1	Model 2	Model 3
Use: CNN	0.840***	0.817***	0.611***
	(0.133)	(0.142)	(0.149)
Use: Fox News	−0.513***	−0.565***	−0.305**
	(0.113)	(0.120)	(0.130)
Use: MSNBC	0.025	−0.066	−0.157
	(0.163)	(0.173)	(0.178)
Use: Network TV (ABC, NBC, CBS)	0.551***	0.520***	0.442***
	(0.115)	(0.123)	(0.126)
Use: PBS	0.138	−0.029	−0.076

	(0.153)	(0.163)	(0.167)
Use: Facebook	−0.406***	−0.256**	−0.234*
	(0.114)	(0.124)	(0.128)
Use: Other Social Media	−0.107	0.132	0.021
	(0.115)	(0.140)	(0.143)
Use: None of these	−1.832***	−1.653***	−1.544***
	(0.449)	(0.472)	(0.475)
Female		0.320***	0.289**
		(0.116)	(0.119)
Age Group: 25-34		−0.182	−0.172
		(0.211)	(0.215)
Age Group: 35-44		−0.123	−0.042
		(0.213)	(0.219)
Age Group: 45-54		0.192	0.315

	(0.232)	(0.238)
Age Group: 55-64	0.937***	1.117***
	(0.241)	(0.248)
Age Group: 65+	1.193***	1.406***
	(0.262)	(0.270)
Race: Black, Non-Hispanic	−0.137	−0.490**
	(0.207)	(0.220)
Race: Hispanic	0.220	0.069
	(0.135)	(0.141)
Race: Asian, Non-Hispanic	0.867***	0.898***
	(0.215)	(0.220)
Race: Other Race	0.329	0.218
	(0.653)	(0.693)
College	0.937***	0.855***

		(0.130)	(0.133)
Children in Household		−0.216	−0.239*
		(0.134)	(0.137)
Party ID: Democrat			0.493***
			(0.155)
Party ID: Republican			0.114
			(0.168)
Ideology (5pt, Higher = More Conservative)			−0.267***
			(0.053)
Politically Engaged			0.234*
			(0.121)
Num.Obs.	1762	1762	1762
AIC	2066.4	1921.6	1867.4
BIC	2115.7	2036.6	2004.2

Log.Lik.	−1024.207	−939.816	−908.689
RMSE	0.45	0.42	0.41

* p < 0.1, ** p < 0.05, *** p < 0.01

Table B9: Logistic Regression, Vaccine Resistance by News Type (CBS/YouGov)

	**Simple**	**Demo Controls**	**Demo + Geo**	**Demo + Geo + Party**
(Intercept)	−1.419	−1.057	−1.748	−3.309
	(0.067)	(0.218)	(0.276)	(0.328)
Facebook Use: Facebook plus other(s)	−0.351	−0.373	−0.374	−0.327
	(0.131)	(0.136)	(0.137)	(0.141)
Facebook Use: Only Facebook	0.778	0.541	0.512	0.446
	(0.174)	(0.183)	(0.186)	(0.194)
Race: Black		−0.432	−0.409	0.118
		(0.183)	(0.188)	(0.202)

Race: Hispanic	−0.340	−0.251	−0.129
	(0.154)	(0.158)	(0.163)
Race: Other	−0.154	−0.080	0.016
	(0.214)	(0.216)	(0.223)
Female	0.158	0.110	0.201
	(0.116)	(0.118)	(0.122)
Age Group: 30-44	0.358	0.370	0.546
	(0.181)	(0.182)	(0.187)
Age Group: 45-64	0.303	0.238	0.361
	(0.160)	(0.162)	(0.167)
Age Group: 65+	−0.413	−0.476	−0.337
	(0.201)	(0.203)	(0.209)
College	−0.925	−0.875	−0.757

	(0.146)	(0.147)	(0.150)
Income Group: $50-100k	−0.355	−0.306	−0.399
	(0.145)	(0.147)	(0.152)
Income Group: Over $100k	−0.301	−0.207	−0.139
	(0.186)	(0.189)	(0.196)
Income Group: Prefer not to say	0.321	0.376	0.308
	(0.166)	(0.168)	(0.173)
No Children in Household	−0.115	−0.126	−0.140
	(0.145)	(0.147)	(0.152)
Census Region: Midwest		0.412	0.395
		(0.203)	(0.208)
Census Region: South		0.604	0.533
		(0.182)	(0.187)

Census Region: West	0.297	0.283
	(0.197)	(0.203)
Area Type: Suburb	0.182	0.088
	(0.148)	(0.153)
Area Type: Town/Rural area	0.640	0.533
	(0.143)	(0.149)
Area Type: Other	0.655	0.525
	(0.552)	(0.548)
Party ID: Republican		2.003
		(0.200)
Party ID: Independent/Other		1.651
		(0.194)
Party ID: Not sure		1.615

				(0.246)
Num.Obs.	2216	2214	2213	2213
AIC	2143.5	2054.7	2027.6	1896.3
BIC	2160.6	2140.2	2147.3	2033.1
Log.Lik.	−1068.764	−1012.335	−992.776	−924.138
RMSE	0.98	0.96	0.95	0.92

Table B10: Logistic Regression, Vaccination by Social Media Use for COVID-19 Information in Previous 24 Hours (September 2021 Wave)

	Model 1	Model 2	Model 3	Model 4
Use: Facebook	−0.325***	−0.136***	−0.111***	−0.105***
	(0.031)	(0.034)	(0.034)	(0.035)
Use: Twitter	0.364***	0.350***	0.336***	0.191***
	(0.054)	(0.056)	(0.056)	(0.058)
Use: YouTube	−0.372***	−0.232***	−0.236***	−0.260***

	(0.041)	(0.044)	(0.044)	(0.046)
Use: Reddit	0.314***	0.413***	0.407***	0.362***
	(0.083)	(0.086)	(0.087)	(0.090)
Use: Instagram	0.049	0.124**	0.089	0.045
	(0.053)	(0.056)	(0.057)	(0.059)
Use: Snapchat	−0.260***	−0.127	−0.105	−0.085
	(0.088)	(0.092)	(0.092)	(0.096)
Use: Wikipedia	0.186**	0.101	0.100	0.067
	(0.093)	(0.097)	(0.097)	(0.101)
Use: Facebook Messenger	−0.292***	−0.292***	−0.276***	−0.293***
	(0.065)	(0.069)	(0.069)	(0.072)
Use: WhatsApp	0.497***	0.158	0.133	0.018
	(0.112)	(0.116)	(0.116)	(0.121)
Use: TikTok	−0.417***	−0.077	−0.061	−0.059

	(0.065)	(0.069)	(0.069)	(0.072)
Age Group: 25-44		−0.094	−0.086	−0.081
		(0.058)	(0.058)	(0.060)
Age Group: 45-64		0.416***	0.425***	0.552***
		(0.060)	(0.060)	(0.063)
Age Group: 65+		1.107***	1.106***	1.282***
		(0.069)	(0.069)	(0.072)
Race: Latino		0.303***	0.254***	0.127**
		(0.060)	(0.061)	(0.064)
Race: Black		−0.015	−0.009	−0.455***
		(0.054)	(0.056)	(0.060)
Race: Asian		0.839***	0.773***	0.692***
		(0.085)	(0.086)	(0.088)
Race: Other Race		−0.112	−0.118	−0.252***

	(0.087)	(0.087)	(0.091)
Female	−0.023	−0.008	−0.050
	(0.036)	(0.036)	(0.038)
College	0.724***	0.704***	0.595***
	(0.038)	(0.038)	(0.040)
Income (9pt)	1.246***	1.167***	1.341***
	(0.073)	(0.073)	(0.076)
Children in Household	−0.432***	−0.417***	−0.406***
	(0.038)	(0.038)	(0.040)
Suburban		0.355***	0.273***
		(0.042)	(0.044)
Urban		0.442***	0.273***
		(0.052)	(0.054)
Party ID			−2.033***

				(0.061)
Num.Obs.	21079	21079	21079	20974
AIC	25034.1	22909.4	22719.6	21400.5
BIC	25121.6	23084.5	22934.4	21623.2
Log.Lik.	−12506.045	−11432.719	−11332.803	−10672.263

* p < 0.1, ** p < 0.05, *** p < 0.01

Table B11: Logistic Regression, Vaccination by Social Media Importance for COVID-19 Information (September 2021 Wave)

	Model 1	Model 2	Model 3	Model 4
Importance: Facebook	−0.082***	−0.019	−0.001	0.006
	(0.024)	(0.025)	(0.026)	(0.026)
Importance: Twitter	0.262***	0.225***	0.218***	0.151***
	(0.029)	(0.030)	(0.030)	(0.031)
Importance: YouTube	−0.253***	−0.173***	−0.172***	−0.178***
	(0.023)	(0.025)	(0.025)	(0.026)

Importance: Reddit	−0.046	−0.004	−0.006	−0.006
	(0.031)	(0.032)	(0.032)	(0.034)
Importance: Instagram	0.092***	0.124***	0.109***	0.085**
	(0.032)	(0.033)	(0.033)	(0.035)
Importance: Snapchat	−0.101***	−0.075*	−0.064	−0.040
	(0.038)	(0.039)	(0.039)	(0.041)
Importance: Wikipedia	0.148***	0.164***	0.155***	0.126***
	(0.024)	(0.025)	(0.025)	(0.026)
Importance: Facebook Messenger	−0.108***	−0.131***	−0.125***	−0.116***
	(0.030)	(0.032)	(0.032)	(0.033)
Importance: WhatsApp	0.108***	−0.051	−0.056	−0.058
	(0.036)	(0.038)	(0.038)	(0.039)
Importance: TikTok	−0.139***	0.002	0.008	0.013
	(0.031)	(0.032)	(0.033)	(0.034)

Age Group: 25-44	−0.123*	−0.111*	−0.089
	(0.066)	(0.067)	(0.069)
Age Group: 45-64	0.411***	0.429***	0.559***
	(0.068)	(0.068)	(0.071)
Age Group: 65+	1.121***	1.120***	1.286***
	(0.078)	(0.079)	(0.082)
Race: Latino	0.312***	0.255***	0.141**
	(0.066)	(0.067)	(0.070)
Race: Black	−0.045	−0.009	−0.418***
	(0.060)	(0.061)	(0.065)
Race: Asian	0.793***	0.706***	0.654***
	(0.096)	(0.097)	(0.100)
Race: Other Race	−0.170	−0.202*	−0.314***
	(0.105)	(0.106)	(0.109)

Female	−0.034	−0.023	−0.065
	(0.041)	(0.041)	(0.042)
College	0.716***	0.698***	0.596***
	(0.043)	(0.043)	(0.045)
Income (9pt)	1.287***	1.191***	1.341***
	(0.083)	(0.084)	(0.087)
Children in Household	−0.470***	−0.450***	−0.433***
	(0.044)	(0.044)	(0.045)
Suburban		0.288***	0.211***
		(0.051)	(0.053)
Urban		0.332***	0.194***
		(0.061)	(0.063)
Party ID			−1.926***
			(0.069)

Num.Obs.	16640	16640	16640	16563
AIC	19567.7	17870.3	17730.7	16805.5
BIC	19652.6	18040.1	17939.2	17021.5
Log.Lik.	−9772.827	−8913.148	−8838.371	−8374.752

* p < 0.1, ** p < 0.05, *** p < 0.01

Table B12: Logistic Regression, "Only Facebook" News Type by Wave

	December 2020 - January 2021	February 2021	April 2021	June-July 2021
Age Group: 25-44	0.255***	0.241***	0.288***	0.242***
	(0.059)	(0.070)	(0.071)	(0.061)
Age Group: 45-64	−0.352***	−0.307***	−0.279***	−0.315***
	(0.063)	(0.071)	(0.072)	(0.064)
Age Group: 65+	−0.948***	−0.803***	−0.796***	−0.920***
	(0.082)	(0.083)	(0.083)	(0.078)
Race: Latino	−0.130	−0.234***	−0.285***	−0.153**

	(0.080)	(0.088)	(0.090)	(0.074)
Race: Black	−0.482***	−0.577***	−0.516***	−0.426***
	(0.076)	(0.085)	(0.085)	(0.072)
Race: Asian	−0.347***	−0.361***	−0.286***	−0.175*
	(0.100)	(0.104)	(0.100)	(0.093)
Race: Other Race	−0.041	−0.201*	−0.348***	−0.124
	(0.094)	(0.114)	(0.124)	(0.115)
Female	0.607***	0.507***	0.540***	0.475***
	(0.044)	(0.044)	(0.045)	(0.045)
College	−0.231***	−0.163***	−0.167***	−0.211***
	(0.045)	(0.043)	(0.046)	(0.046)
Children in Household	0.156***	0.292***	0.408***	0.283***
	(0.043)	(0.045)	(0.047)	(0.044)
Income (9pt)	−0.592***	−0.464***	−0.894***	−0.744***

	(0.075)	(0.086)	(0.093)	(0.091)
Census Region: Midwest	0.207***	0.138**	0.130**	0.204***
	(0.060)	(0.060)	(0.061)	(0.063)
Census Region: South	0.112*	0.101*	−0.010	0.137**
	(0.058)	(0.057)	(0.058)	(0.060)
Census Region: West	0.097	0.057	0.018	0.108*
	(0.060)	(0.061)	(0.063)	(0.064)
Suburban	−0.195***	−0.213***	−0.184***	−0.181***
	(0.045)	(0.046)	(0.050)	(0.049)
Urban	−0.503***	−0.358***	−0.411***	−0.423***
	(0.063)	(0.061)	(0.063)	(0.063)
Party ID	0.587***	0.614***	0.654***	0.644***
	(0.066)	(0.065)	(0.067)	(0.066)

Num.Obs.	25494	21164	21211	20569
AIC	19013.7	18123.3	17000.4	17568.9
BIC	19160.3	18266.6	17143.7	17711.7
Log.Lik.	−9488.835	−9043.666	−8482.205	−8766.449

* p < 0.1, ** p < 0.05, *** p < 0.01

Table B13: Logistic Regression, "None of these" News Type by Wave

	December 2020 - January 2021	February 2021	April 2021	June-July 2021
Age Group: 25-44	0.027	0.116	0.022	0.038
	(0.050)	(0.071)	(0.071)	(0.065)
Age Group: 45-64	0.195***	0.265***	0.128*	0.084
	(0.049)	(0.069)	(0.068)	(0.063)
Age Group: 65+	0.138**	0.086	−0.166**	−0.068
	(0.054)	(0.074)	(0.072)	(0.068)

Race: Latino	−0.525***	−0.389***	−0.455***	−0.439***
	(0.071)	(0.092)	(0.094)	(0.081)
Race: Black	−0.540***	−0.523***	−0.639***	−0.639***
	(0.059)	(0.081)	(0.088)	(0.078)
Race: Asian	−0.382***	−0.285***	−0.349***	−0.513***
	(0.074)	(0.092)	(0.099)	(0.099)
Race: Other Race	−0.184**	0.084	0.007	−0.136
	(0.078)	(0.101)	(0.109)	(0.114)
Female	0.229***	0.241***	0.402***	0.343***
	(0.031)	(0.038)	(0.042)	(0.042)
College	−0.087***	0.042	−0.162***	−0.002
	(0.033)	(0.039)	(0.043)	(0.042)
Children in Household	−0.436***	−0.422***	−0.528***	−0.534***
	(0.037)	(0.046)	(0.050)	(0.047)

Income (9pt)	−0.261***	−0.105	−0.058	−0.009
	(0.056)	(0.079)	(0.087)	(0.086)
Census Region: Midwest	0.147***	0.058	0.165***	0.149**
	(0.045)	(0.054)	(0.057)	(0.058)
Census Region: South	−0.028	−0.055	−0.054	−0.084
	(0.044)	(0.052)	(0.055)	(0.057)
Census Region: West	0.112**	0.188***	0.229***	0.156***
	(0.045)	(0.054)	(0.058)	(0.059)
Suburban	0.084**	0.084*	0.041	0.030
	(0.037)	(0.045)	(0.049)	(0.049)
Urban	0.0004	−0.082	−0.206***	−0.182***
	(0.048)	(0.058)	(0.062)	(0.061)
Party ID	0.556***	0.671***	0.707***	0.686***
	(0.050)	(0.059)	(0.063)	(0.062)

Num.Obs.	25494	21164	21211	20569
AIC	28406.6	20741.0	18649.0	18791.6
BIC	28553.3	20884.3	18792.4	18934.4
Log.Lik.	−14185.321	−10352.512	−9306.524	−9377.805
RMSE	0.43	0.40	0.37	0.38

* p < 0.1, ** p < 0.05, *** p < 0.01
