# Supplementary material for: Media use and vaccine resistance
Source: PNAS Nexus. 2023 May 9;2(5):pgad146. doi: 10.1093/pnasnexus/pgad146 (PMC10178922; doi:10.1093/pnasnexus/pgad146)

Appendix E: Descriptive Statistics by News Type

The primary analyses in the main manuscript rely on a typology of respondents based on their reported sources of news and information about the COVID-19 pandemic in the previous 24 hours across four survey waves. Here, we provide additional descriptive statistics regarding respondents who fall into each of these news types (including those who did not report consuming COVID-19 related news/information from any of the provided sources) for each of those waves.

Overall Share

Table E1. Overall Distributions of News Type

	December 2020–January 2021	February 2021	April 2021	June–July 2021
**Covid News Source**	**N = 25,640**	**N = 21,500**	**N = 21,733**	**N = 20,669**
Multiple (not FB, Fox, Newsmax)	1,902 (7.4%)	1,705 (7.9%)	1,672 (7.7%)	1,421 (6.9%)
Only Biden Admin.	385 (1.5%)	1,264 (5.9%)	1,655 (7.6%)	1,541 (7.5%)
Only CNN	2,806 (11%)	2,302 (11%)	2,331 (11%)	2,258 (11%)
Only MSNBC	785 (3.1%)	752 (3.5%)	750 (3.5%)	759 (3.7%)
Only Fox	3,165 (12%)	3,030 (14%)	2,992 (14%)	2,883 (14%)
Only Facebook	3,100 (12%)	3,224 (15%)	3,132 (14%)	3,289 (16%)
Only Newsmax	507 (2.0%)	500 (2.3%)	411 (1.9%)	441 (2.1%)
Multiple (including FB, Fox, Newsmax)	6,650 (26%)	4,885 (23%)	5,347 (25%)	4,635 (22%)
None of these	6,342 (25%)	3,840 (18%)	3,443 (16%)	3,442 (17%)

Note: Statistics show weighted number of observations and percentage.

Demographic Crosstabs

Table E2. Demographics (December 2020-January 2021)

Variable	Overall, N = 25,642	Multiple (not FB, Fox, Newsmax), N = 1,902	Only Biden Admin., N = 385	Only CNN, N = 2,806	Only MSNBC, N = 785	Only Fox, N = 3,165	Only Facebook N = 3,100	Only Newsmax N = 507	Multiple (inc. FB, Fox, Newsmax), N = 6,650	None of these, N = 6,342
gender										
Female	13,417 (52%)	882 (46%)	204 (53%)	1,442 (51%)	425 (54%)	1,668 (53%)	2,027 (65%)	231 (45%)	2,990 (45%)	3,548 (56%)
Male	12,223 (48%)	1,020 (54%)	181 (47%)	1,364 (49%)	360 (46%)	1,497 (47%)	1,072 (35%)	276 (55%)	3,659 (55%)	2,794 (44%)
age										
18-24	3,219 (13%)	174 (9.1%)	58 (15%)	465 (17%)	62 (7.9%)	252 (8.0%)	405 (13%)	31 (6.0%)	992 (15%)	781 (12%)
24-44	8,871 (35%)	473 (25%)	75 (20%)	917 (33%)	201 (26%)	756 (24%)	1,542 (50%)	123 (24%)	2,987 (45%)	1,797 (28%)
45-64	8,391 (33%)	689 (36%)	133 (34%)	908 (32%)	289 (37%)	1,205 (38%)	880 (28%)	218 (43%)	1,797 (27%)	2,273 (36%)
65+	5,158 (20%)	566 (30%)	119 (31%)	516 (18%)	233 (30%)	951 (30%)	272 (8.8%)	136 (27%)	874 (13%)	1,490 (24%)
race										
White	16,747 (65%)	1,033 (54%)	251 (65%)	1,497 (53%)	500 (64%)	2,213 (70%)	2,164 (70%)	356 (70%)	3,934 (59%)	4,799 (76%)
Hispanic	3,609 (14%)	312 (16%)	44 (11%)	485 (17%)	99 (13%)	332 (10%)	439 (14%)	86 (17%)	1,197 (18%)	614 (9.7%)
Black	3,127 (12%)	378 (20%)	52 (13%)	489 (17%)	114 (15%)	413 (13%)	265 (8.5%)	28 (5.5%)	915 (14%)	473 (7.5%)
Asian	1,498 (5.8%)	129 (6.8%)	29 (7.6%)	271 (9.7%)	54 (6.9%)	141 (4.5%)	131 (4.2%)	20 (4.0%)	439 (6.6%)	282 (4.5%)
Other	659 (2.6%)	49 (2.6%)	9 (2.3%)	62 (2.2%)	17 (2.2%)	65 (2.1%)	101 (3.3%)	18 (3.5%)	164 (2.5%)	174 (2.7%)
region										

Northeast	4,562 (18%)	437 (23%)	72 (19%)	599 (21%)	144 (18%)	448 (14%)	504 (16%)	78 (15%)	1,232 (19%)	1,049 (17%)
Midwest	5,514 (22%)	342 (18%)	98 (26%)	573 (20%)	154 (20%)	686 (22%)	733 (24%)	115 (23%)	1,233 (19%)	1,578 (25%)
South	9,506 (37%)	657 (35%)	110 (29%)	992 (35%)	301 (38%)	1,300 (41%)	1,144 (37%)	182 (36%)	2,610 (39%)	2,211 (35%)
West	6,058 (24%)	466 (24%)	105 (27%)	641 (23%)	186 (24%)	730 (23%)	719 (23%)	132 (26%)	1,575 (24%)	1,505 (24%)
community										
Rural	3,755 (15%)	190 (10%)	49 (13%)	328 (12%)	139 (18%)	448 (14%)	649 (21%)	91 (18%)	871 (13%)	991 (16%)
Suburban	14,264 (56%)	989 (52%)	212 (55%)	1,490 (53%)	426 (54%)	1,775 (56%)	1,783 (58%)	301 (59%)	3,521 (53%)	3,766 (59%)
Urban	7,620 (30%)	723 (38%)	124 (32%)	987 (35%)	220 (28%)	942 (30%)	667 (22%)	115 (23%)	2,258 (34%)	1,585 (25%)
employment										
Employed	13,341 (52%)	929 (49%)	180 (47%)	1,513 (54%)	376 (48%)	1,395 (44%)	1,629 (53%)	255 (50%)	4,181 (63%)	2,882 (45%)
Unemployed	3,705 (14%)	194 (10%)	35 (9.1%)	342 (12%)	127 (16%)	416 (13%)	627 (20%)	56 (11%)	867 (13%)	1,041 (16%)
Homemaker	1,534 (6.0%)	54 (2.9%)	26 (6.7%)	119 (4.2%)	21 (2.7%)	193 (6.1%)	299 (9.7%)	45 (8.9%)	332 (5.0%)	445 (7.0%)
Student	1,359 (5.3%)	101 (5.3%)	26 (6.8%)	237 (8.5%)	22 (2.9%)	103 (3.3%)	145 (4.7%)	7 (1.4%)	375 (5.6%)	342 (5.4%)
Retired	5,694 (22%)	623 (33%)	118 (31%)	592 (21%)	239 (30%)	1,057 (33%)	398 (13%)	143 (28%)	892 (13%)	1,631 (26%)
education										
No college	2,308 (9.0%)	116 (6.1%)	20 (5.1%)	212 (7.5%)	20 (2.5%)	343 (11%)	480 (15%)	58 (11%)	527 (7.9%)	534 (8.4%)
no college	7,211 (28%)	346 (18%)	87 (22%)	690 (25%)	209 (27%)	1,049 (33%)	1,055 (34%)	151 (30%)	1,784 (27%)	1,840 (29%)

Some college	8,128 (32%)	609 (32%)	138 (36%)	849 (30%)	280 (36%)	1,053 (33%)	933 (30%)	169 (33%)	1,929 (29%)	2,170 (34%)
4-year degree	4,904 (19%)	508 (27%)	87 (23%)	625 (22%)	178 (23%)	487 (15%)	424 (14%)	96 (19%)	1,310 (20%)	1,190 (19%)
Grad degree	3,088 (12%)	323 (17%)	54 (14%)	429 (15%)	99 (13%)	233 (7.3%)	209 (6.7%)	33 (6.6%)	1,101 (17%)	608 (9.6%)
income										
Under 25k	7,319 (29%)	392 (21%)	107 (28%)	739 (26%)	188 (24%)	911 (29%)	1,203 (39%)	123 (24%)	1,827 (28%)	1,829 (29%)
25-75k	9,892 (39%)	781 (41%)	149 (39%)	1,037 (37%)	352 (45%)	1,213 (39%)	1,160 (38%)	203 (40%)	2,357 (36%)	2,640 (42%)
75-100k	4,480 (18%)	398 (21%)	69 (18%)	540 (19%)	135 (17%)	533 (17%)	406 (13%)	100 (20%)	1,240 (19%)	1,058 (17%)
Over 150k	3,796 (15%)	327 (17%)	60 (16%)	480 (17%)	106 (14%)	485 (15%)	291 (9.5%)	81 (16%)	1,193 (18%)	773 (12%)
party										
Republican	7,048 (28%)	76 (4.0%)	43 (11%)	321 (12%)	116 (15%)	1,572 (50%)	870 (28%)	303 (60%)	1,800 (27%)	1,946 (31%)
Democrat	9,461 (37%)	1,349 (71%)	216 (56%)	1,588 (57%)	418 (54%)	688 (22%)	780 (25%)	29 (5.7%)	2,699 (41%)	1,694 (27%)
Indep./Other	9,013 (35%)	474 (25%)	126 (33%)	881 (32%)	245 (31%)	901 (28%)	1,436 (47%)	175 (35%)	2,112 (32%)	2,664 (42%)

Note: Statistics show weighted number of observations and percentage belonging to each gender, region, party, etc.

Table E3. Demographics (February 2021)

**Variable**	**Overall, N = 21,502**	**Multiple (not FB, Fox, Newsmax), N = 1,705**	**Only Biden Admin., N = 1,264**	**Only CNN, N = 2,302**	**Only MSNBC, N = 752**	**Only Fox, N = 3,030**	**Only Facebook, N = 3,224**	**Only Newsmax, N = 500**	**Multiple (including FB, Fox, Newsmax), N = 4,885**	**None of these, N = 3,840**
gender										

Female	11,307 (53%)	777 (46%)	704 (56%)	1,173 (51%)	382 (51%)	1,593 (53%)	2,041 (63%)	238 (48%)	2,236 (46%)	2,162 (56%)
Male	10,193 (47%)	928 (54%)	560 (44%)	1,129 (49%)	370 (49%)	1,437 (47%)	1,182 (37%)	261 (52%)	2,648 (54%)	1,678 (44%)
age										
18-24	2,633 (12%)	206 (12%)	137 (11%)	366 (16%)	60 (7.9%)	264 (8.7%)	405 (13%)	18 (3.5%)	744 (15%)	434 (11%)
24-44	7,348 (34%)	427 (25%)	278 (22%)	718 (31%)	188 (25%)	722 (24%)	1,524 (47%)	98 (20%)	2,281 (47%)	1,112 (29%)
45-64	7,111 (33%)	546 (32%)	425 (34%)	779 (34%)	297 (39%)	1,126 (37%)	940 (29%)	229 (46%)	1,299 (27%)	1,470 (38%)
65+	4,408 (21%)	526 (31%)	424 (34%)	439 (19%)	208 (28%)	918 (30%)	355 (11%)	155 (31%)	560 (11%)	824 (21%)
race										
White	14,091 (66%)	927 (54%)	854 (68%)	1,235 (54%)	454 (60%)	2,137 (71%)	2,303 (71%)	369 (74%)	2,924 (60%)	2,889 (75%)
Hispanic	3,006 (14%)	294 (17%)	128 (10%)	380 (17%)	108 (14%)	313 (10%)	444 (14%)	81 (16%)	867 (18%)	391 (10%)
Black	2,602 (12%)	330 (19%)	172 (14%)	403 (18%)	140 (19%)	378 (12%)	241 (7.5%)	17 (3.4%)	660 (14%)	260 (6.8%)
Asian	1,262 (5.9%)	116 (6.8%)	77 (6.1%)	216 (9.4%)	35 (4.7%)	134 (4.4%)	147 (4.6%)	22 (4.4%)	315 (6.4%)	199 (5.2%)
Other	540 (2.5%)	39 (2.3%)	32 (2.5%)	67 (2.9%)	14 (1.9%)	69 (2.3%)	89 (2.8%)	11 (2.1%)	119 (2.4%)	101 (2.6%)
region										
Northeast	3,848 (18%)	347 (20%)	270 (21%)	503 (22%)	132 (18%)	434 (14%)	508 (16%)	86 (17%)	900 (18%)	667 (17%)
Midwest	4,603 (21%)	333 (20%)	330 (26%)	439 (19%)	162 (22%)	643 (21%)	739 (23%)	116 (23%)	956 (20%)	885 (23%)
South	7,964 (37%)	629 (37%)	395 (31%)	845 (37%)	254 (34%)	1,302 (43%)	1,203 (37%)	207 (41%)	1,886 (39%)	1,244 (32%)

West	5,086 (24%)	397 (23%)	269 (21%)	515 (22%)	204 (27%)	651 (21%)	773 (24%)	91 (18%)	1,142 (23%)	1,044 (27%)
community										
Rural	3,113 (14%)	180 (11%)	138 (11%)	282 (12%)	104 (14%)	434 (14%)	645 (20%)	83 (17%)	657 (13%)	589 (15%)
Suburban	11,990 (56%)	888 (52%)	765 (61%)	1,237 (54%)	433 (58%)	1,702 (56%)	1,812 (56%)	318 (64%)	2,560 (52%)	2,273 (59%)
Urban	6,397 (30%)	637 (37%)	360 (28%)	783 (34%)	215 (29%)	893 (29%)	766 (24%)	98 (20%)	1,667 (34%)	977 (25%)
employment										
Employed	11,055 (51%)	823 (48%)	504 (40%)	1,148 (50%)	357 (48%)	1,317 (43%)	1,812 (56%)	197 (39%)	3,057 (63%)	1,840 (48%)
Unemployed	3,061 (14%)	167 (9.8%)	176 (14%)	315 (14%)	109 (14%)	419 (14%)	577 (18%)	74 (15%)	648 (13%)	576 (15%)
Homemaker	1,235 (5.7%)	58 (3.4%)	74 (5.9%)	122 (5.3%)	32 (4.3%)	186 (6.1%)	237 (7.4%)	28 (5.7%)	263 (5.4%)	235 (6.1%)
Student	1,062 (4.9%)	108 (6.3%)	68 (5.4%)	177 (7.7%)	20 (2.6%)	84 (2.8%)	140 (4.4%)	14 (2.9%)	258 (5.3%)	193 (5.0%)
Retired	5,072 (24%)	549 (32%)	441 (35%)	537 (23%)	234 (31%)	1,024 (34%)	456 (14%)	186 (37%)	659 (13%)	987 (26%)
education										
No college	2,062 (9.6%)	84 (4.9%)	91 (7.2%)	244 (11%)	69 (9.2%)	434 (14%)	387 (12%)	47 (9.4%)	427 (8.7%)	279 (7.3%)
no college	5,980 (28%)	394 (23%)	334 (26%)	598 (26%)	204 (27%)	1,018 (34%)	1,068 (33%)	124 (25%)	1,249 (26%)	992 (26%)
Some college	6,749 (31%)	578 (34%)	434 (34%)	656 (28%)	255 (34%)	879 (29%)	956 (30%)	187 (37%)	1,416 (29%)	1,390 (36%)
4-year degree	4,159 (19%)	412 (24%)	261 (21%)	468 (20%)	150 (20%)	456 (15%)	509 (16%)	101 (20%)	1,009 (21%)	792 (21%)
Grad degree	2,549 (12%)	238 (14%)	143 (11%)	335 (15%)	74 (9.8%)	243 (8.0%)	304 (9.4%)	41 (8.1%)	784 (16%)	386 (10%)

income										
Over 150k	1,422 (6.6%)	141 (8.2%)	57 (4.5%)	195 (8.5%)	46 (6.1%)	170 (5.6%)	141 (4.4%)	28 (5.6%)	427 (8.8%)	217 (5.7%)
Under 25k	6,705 (31%)	433 (25%)	433 (34%)	704 (31%)	228 (30%)	980 (32%)	1,183 (37%)	138 (28%)	1,478 (30%)	1,128 (29%)
75-100k	4,512 (21%)	390 (23%)	228 (18%)	504 (22%)	140 (19%)	575 (19%)	615 (19%)	127 (25%)	1,155 (24%)	779 (20%)
25-75k	8,838 (41%)	742 (44%)	546 (43%)	897 (39%)	336 (45%)	1,304 (43%)	1,279 (40%)	207 (41%)	1,816 (37%)	1,712 (45%)
party										
Republican	5,689 (27%)	66 (3.8%)	166 (13%)	296 (13%)	125 (17%)	1,458 (48%)	982 (31%)	294 (59%)	1,202 (25%)	1,102 (29%)
Democrat	7,864 (37%)	1,167 (69%)	642 (51%)	1,276 (56%)	376 (50%)	580 (19%)	856 (27%)	24 (4.9%)	2,017 (41%)	926 (24%)
Indep./Other	7,867 (37%)	471 (28%)	455 (36%)	726 (32%)	250 (33%)	988 (33%)	1,370 (43%)	177 (36%)	1,655 (34%)	1,775 (47%)

Note: Statistics show weighted number of observations and percentage belonging to each gender, region, party, etc.

Table E4. Demographics (April 2021)

Variable	Overall, N = 21,733	Multiple (not FB, Fox, Newsmax), N = 1,672	Only Biden Admin., N = 1,655	Only CNN, N = 2,331	Only MSNBC, N = 750	Only Fox, N = 2,992	Only Facebook, N = 3,132	Only Newsmax, N = 411	Multiple (including FB, Fox, Newsmax), N = 5,347	None of these, N = 3,443
gender										
Female	11,358 (52%)	781 (47%)	871 (53%)	1,226 (53%)	435 (58%)	1,601 (54%)	2,044 (65%)	176 (43%)	2,180 (41%)	2,044 (59%)
Male	10,375 (48%)	891 (53%)	784 (47%)	1,105 (47%)	316 (42%)	1,391 (46%)	1,087 (35%)	235 (57%)	3,167 (59%)	1,399 (41%)
age										

18-24	2,708 (12%)	188 (11%)	180 (11%)	416 (18%)	58 (7.7%)	235 (7.9%)	394 (13%)	25 (6.2%)	759 (14%)	452 (13%)
24-44	7,469 (34%)	429 (26%)	312 (19%)	763 (33%)	147 (20%)	616 (21%)	1,497 (48%)	100 (24%)	2,637 (49%)	968 (28%)
45-64	7,090 (33%)	551 (33%)	624 (38%)	731 (31%)	284 (38%)	1,200 (40%)	905 (29%)	173 (42%)	1,334 (25%)	1,288 (37%)
65+	4,466 (21%)	505 (30%)	538 (33%)	421 (18%)	262 (35%)	941 (31%)	335 (11%)	112 (27%)	617 (12%)	734 (21%)
race										
White	14,194 (65%)	907 (54%)	1,046 (63%)	1,224 (53%)	459 (61%)	2,100 (70%)	2,226 (71%)	296 (72%)	3,281 (61%)	2,656 (77%)
Hispanic	3,131 (14%)	302 (18%)	257 (16%)	411 (18%)	98 (13%)	410 (14%)	414 (13%)	55 (13%)	857 (16%)	326 (9.5%)
Black	2,612 (12%)	309 (18%)	226 (14%)	415 (18%)	147 (20%)	293 (9.8%)	284 (9.1%)	21 (5.0%)	721 (13%)	197 (5.7%)
Asian	1,256 (5.8%)	116 (6.9%)	91 (5.5%)	231 (9.9%)	28 (3.7%)	124 (4.1%)	130 (4.2%)	22 (5.5%)	348 (6.5%)	166 (4.8%)
Other	540 (2.5%)	39 (2.3%)	34 (2.1%)	50 (2.1%)	19 (2.5%)	65 (2.2%)	78 (2.5%)	17 (4.1%)	140 (2.6%)	98 (2.8%)
region										
Northeast	3,888 (18%)	325 (19%)	341 (21%)	488 (21%)	142 (19%)	486 (16%)	512 (16%)	69 (17%)	962 (18%)	564 (16%)
Midwest	4,566 (21%)	304 (18%)	376 (23%)	399 (17%)	157 (21%)	615 (21%)	767 (25%)	85 (21%)	1,050 (20%)	811 (24%)
South	8,147 (37%)	626 (37%)	559 (34%)	888 (38%)	270 (36%)	1,259 (42%)	1,161 (37%)	131 (32%)	2,100 (39%)	1,152 (33%)
West	5,132 (24%)	417 (25%)	378 (23%)	555 (24%)	182 (24%)	632 (21%)	692 (22%)	126 (31%)	1,235 (23%)	915 (27%)
community										
Rural	3,113 (14%)	171 (10%)	223 (13%)	250 (11%)	125 (17%)	380 (13%)	609 (19%)	73 (18%)	660 (12%)	622 (18%)

Suburban	12,042 (55%)	880 (53%)	920 (56%)	1,254 (54%)	401 (53%)	1,708 (57%)	1,839 (59%)	259 (63%)	2,694 (50%)	2,086 (61%)
Urban	6,579 (30%)	622 (37%)	512 (31%)	828 (36%)	225 (30%)	904 (30%)	683 (22%)	79 (19%)	1,992 (37%)	734 (21%)
employment										
Employed	11,366 (52%)	836 (50%)	630 (38%)	1,200 (52%)	343 (46%)	1,257 (42%)	1,746 (56%)	166 (41%)	3,623 (68%)	1,564 (46%)
Unemployed	2,629 (12%)	150 (9.0%)	220 (13%)	285 (12%)	79 (11%)	368 (12%)	478 (15%)	48 (12%)	495 (9.3%)	505 (15%)
Homemaker	1,381 (6.4%)	50 (3.0%)	101 (6.1%)	127 (5.4%)	46 (6.1%)	193 (6.5%)	296 (9.5%)	28 (6.8%)	271 (5.1%)	270 (7.9%)
Student	1,257 (5.8%)	113 (6.8%)	107 (6.4%)	226 (9.7%)	18 (2.4%)	102 (3.4%)	175 (5.6%)	15 (3.8%)	271 (5.1%)	230 (6.7%)
Retired	5,074 (23%)	522 (31%)	597 (36%)	492 (21%)	264 (35%)	1,066 (36%)	433 (14%)	153 (37%)	683 (13%)	864 (25%)
education										
No college	2,032 (9.3%)	76 (4.5%)	119 (7.2%)	246 (11%)	46 (6.2%)	328 (11%)	419 (13%)	35 (8.6%)	452 (8.4%)	311 (9.0%)
no college	5,969 (27%)	356 (21%)	461 (28%)	604 (26%)	233 (31%)	1,115 (37%)	998 (32%)	129 (31%)	1,130 (21%)	943 (27%)
Some college	6,896 (32%)	529 (32%)	558 (34%)	711 (30%)	253 (34%)	935 (31%)	977 (31%)	156 (38%)	1,495 (28%)	1,282 (37%)
4-year degree	4,232 (19%)	464 (28%)	354 (21%)	466 (20%)	144 (19%)	440 (15%)	482 (15%)	56 (14%)	1,187 (22%)	639 (19%)
Grad degree	2,605 (12%)	247 (15%)	163 (9.9%)	305 (13%)	74 (9.9%)	173 (5.8%)	255 (8.2%)	35 (8.6%)	1,084 (20%)	267 (7.8%)
income										
75-100k	5,376 (25%)	456 (27%)	387 (23%)	554 (24%)	156 (21%)	593 (20%)	640 (20%)	84 (21%)	1,692 (32%)	814 (24%)
25-75k	9,474 (44%)	785 (47%)	764 (46%)	1,022 (44%)	375 (50%)	1,421 (48%)	1,336 (43%)	228 (56%)	1,975 (37%)	1,567 (46%)

Over 150k	1,511 (7.0%)	133 (8.0%)	84 (5.1%)	183 (7.9%)	34 (4.5%)	121 (4.1%)	121 (3.9%)	10 (2.4%)	626 (12%)	199 (5.8%)
Under 25k	5,369 (25%)	298 (18%)	419 (25%)	572 (25%)	186 (25%)	857 (29%)	1,035 (33%)	89 (22%)	1,054 (20%)	861 (25%)
party										
Republican	5,487 (25%)	71 (4.2%)	255 (15%)	263 (11%)	120 (16%)	1,403 (47%)	917 (29%)	228 (56%)	1,235 (23%)	995 (29%)
Democrat	8,788 (41%)	1,216 (73%)	822 (50%)	1,327 (57%)	402 (54%)	644 (22%)	931 (30%)	34 (8.5%)	2,539 (48%)	874 (25%)
Indep./Other	7,392 (34%)	383 (23%)	578 (35%)	729 (31%)	228 (30%)	932 (31%)	1,276 (41%)	146 (36%)	1,561 (29%)	1,559 (45%)

Note: Statistics show weighted number of observations and percentage belonging to each gender, region, party, etc.

Table E5. Demographics (June-July 2021)

Variable	Overall, N = 20,669	Multiple (not FB, Fox, Newsmax), N = 1,421	Only Biden Admin., N = 1,541	Only CNN, N = 2,258	Only MSNBC, N = 759	Only Fox, N = 2,883	Only Facebook, N = 3,289	Only Newsmax, N = 441	Multiple (including FB, Fox, Newsmax), N = 4,635	None of these, N = 3,442
gender										
Female	10,834 (52%)	638 (45%)	817 (53%)	1,155 (51%)	422 (56%)	1,613 (56%)	2,093 (64%)	221 (50%)	1,873 (40%)	2,001 (58%)
Male	9,835 (48%)	784 (55%)	723 (47%)	1,103 (49%)	336 (44%)	1,270 (44%)	1,197 (36%)	220 (50%)	2,763 (60%)	1,441 (42%)
age										
18-24	2,576 (12%)	161 (11%)	178 (12%)	434 (19%)	51 (6.7%)	257 (8.9%)	439 (13%)	22 (5.0%)	594 (13%)	440 (13%)
24-44	7,093 (34%)	330 (23%)	297 (19%)	658 (29%)	179 (24%)	617 (21%)	1,568 (48%)	85 (19%)	2,361 (51%)	997 (29%)
45-64	6,783 (33%)	492 (35%)	554 (36%)	762 (34%)	273 (36%)	1,134 (39%)	950 (29%)	214 (49%)	1,184 (26%)	1,220 (35%)

65+	4,217 (20%)	439 (31%)	511 (33%)	404 (18%)	255 (34%)	874 (30%)	332 (10%)	120 (27%)	497 (11%)	784 (23%)
race										
White	13,307 (64%)	780 (55%)	1,003 (65%)	1,215 (54%)	480 (63%)	1,926 (67%)	2,229 (68%)	310 (70%)	2,748 (59%)	2,616 (76%)
Hispanic	3,114 (15%)	198 (14%)	215 (14%)	425 (19%)	102 (13%)	436 (15%)	479 (15%)	66 (15%)	822 (18%)	372 (11%)
Black	2,535 (12%)	292 (21%)	205 (13%)	380 (17%)	108 (14%)	327 (11%)	311 (9.4%)	34 (7.7%)	644 (14%)	235 (6.8%)
Asian	1,196 (5.8%)	121 (8.5%)	84 (5.5%)	194 (8.6%)	51 (6.7%)	116 (4.0%)	167 (5.1%)	17 (4.0%)	307 (6.6%)	138 (4.0%)
Other	516 (2.5%)	31 (2.2%)	34 (2.2%)	43 (1.9%)	18 (2.4%)	78 (2.7%)	104 (3.2%)	13 (3.0%)	114 (2.5%)	81 (2.3%)
region										
Northeast	3,695 (18%)	321 (23%)	297 (19%)	499 (22%)	160 (21%)	463 (16%)	476 (14%)	58 (13%)	848 (18%)	574 (17%)
Midwest	4,361 (21%)	276 (19%)	365 (24%)	399 (18%)	165 (22%)	571 (20%)	777 (24%)	78 (18%)	889 (19%)	841 (24%)
South	7,767 (38%)	514 (36%)	555 (36%)	832 (37%)	235 (31%)	1,181 (41%)	1,250 (38%)	183 (42%)	1,853 (40%)	1,162 (34%)
West	4,847 (23%)	310 (22%)	324 (21%)	528 (23%)	199 (26%)	668 (23%)	786 (24%)	122 (28%)	1,045 (23%)	865 (25%)
community										
Rural	2,945 (14%)	138 (9.7%)	203 (13%)	246 (11%)	107 (14%)	378 (13%)	650 (20%)	86 (20%)	588 (13%)	548 (16%)
Suburban	11,449 (55%)	767 (54%)	835 (54%)	1,202 (53%)	440 (58%)	1,635 (57%)	1,876 (57%)	251 (57%)	2,351 (51%)	2,093 (61%)
Urban	6,275 (30%)	517 (36%)	502 (33%)	810 (36%)	212 (28%)	870 (30%)	764 (23%)	104 (24%)	1,696 (37%)	800 (23%)
employment										

Employed	10,673 (52%)	689 (49%)	611 (40%)	1,209 (54%)	308 (41%)	1,194 (41%)	1,754 (53%)	170 (38%)	3,136 (68%)	1,601 (47%)
Unemployed	2,841 (14%)	141 (9.9%)	197 (13%)	297 (13%)	106 (14%)	428 (15%)	583 (18%)	61 (14%)	493 (11%)	535 (16%)
Homemaker	1,357 (6.6%)	43 (3.0%)	75 (4.9%)	105 (4.6%)	41 (5.4%)	235 (8.1%)	372 (11%)	33 (7.6%)	222 (4.8%)	229 (6.7%)
Student	903 (4.4%)	76 (5.3%)	81 (5.3%)	164 (7.3%)	24 (3.2%)	74 (2.6%)	123 (3.7%)	9 (2.1%)	185 (4.0%)	167 (4.9%)
Retired	4,889 (24%)	471 (33%)	576 (37%)	482 (21%)	279 (37%)	953 (33%)	456 (14%)	168 (38%)	599 (13%)	906 (26%)
education										
No college	1,977 (9.6%)	71 (5.0%)	130 (8.4%)	195 (8.6%)	67 (8.8%)	398 (14%)	471 (14%)	34 (7.6%)	334 (7.2%)	278 (8.1%)
no college	5,722 (28%)	280 (20%)	409 (27%)	605 (27%)	209 (28%)	989 (34%)	1,103 (34%)	147 (33%)	1,029 (22%)	950 (28%)
Some college	6,521 (32%)	467 (33%)	519 (34%)	724 (32%)	281 (37%)	896 (31%)	976 (30%)	159 (36%)	1,244 (27%)	1,254 (36%)
4-year degree	4,001 (19%)	370 (26%)	295 (19%)	460 (20%)	141 (19%)	413 (14%)	505 (15%)	69 (16%)	1,101 (24%)	648 (19%)
Grad degree	2,449 (12%)	234 (16%)	188 (12%)	274 (12%)	60 (7.9%)	187 (6.5%)	234 (7.1%)	32 (7.3%)	928 (20%)	312 (9.1%)
income										
75-100k	4,727 (23%)	389 (27%)	312 (20%)	515 (23%)	148 (20%)	564 (20%)	606 (18%)	95 (22%)	1,379 (30%)	718 (21%)
Over 150k	1,259 (6.1%)	93 (6.6%)	87 (5.7%)	137 (6.1%)	24 (3.1%)	113 (3.9%)	124 (3.8%)	19 (4.3%)	471 (10%)	190 (5.5%)
25-75k	9,186 (44%)	679 (48%)	714 (46%)	1,008 (45%)	397 (52%)	1,328 (46%)	1,466 (45%)	206 (47%)	1,773 (38%)	1,613 (47%)
Under 25k	5,498 (27%)	259 (18%)	427 (28%)	598 (26%)	190 (25%)	877 (30%)	1,094 (33%)	121 (27%)	1,013 (22%)	919 (27%)
party										

Republican	5,083 (25%)	39 (2.7%)	250 (16%)	242 (11%)	94 (12%)	1,306 (45%)	879 (27%)	247 (56%)	1,063 (23%)	964 (28%)
Democrat	8,280 (40%)	1,032 (73%)	790 (51%)	1,294 (57%)	414 (55%)	653 (23%)	1,005 (31%)	40 (9.1%)	2,170 (47%)	882 (26%)
Indep./Other	7,248 (35%)	348 (25%)	498 (32%)	720 (32%)	250 (33%)	919 (32%)	1,395 (43%)	152 (35%)	1,391 (30%)	1,574 (46%)

Note: Statistics show weighted number of observations and percentage belonging to each gender, region, party, etc.

Institutional Trust and COVID-19 News Interest: Crosstabs

Table E6. Trust in Institutions (December 2020-January 2021)

Variable	Overall, N = 25,642	Multiple (not FB, Fox, Newsmax), N = 1,902	Only Biden Admin., N = 385	Only CNN, N = 2,806	Only MSNBC, N = 785	Only Fox, N = 3,165	Only Facebook, N = 3,100	Only Newsmax, N = 507	Multiple (including FB, Fox, Newsmax), N = 6,650	None of these, N = 6,342
trust White House	2.3 (1.0)	1.6 (0.8)	1.9 (0.9)	1.9 (1.0)	1.9 (1.0)	2.8 (0.9)	2.3 (1.0)	2.9 (1.0)	2.5 (1.0)	2.2 (1.0)
trust CDC	3.1 (0.9)	3.4 (0.7)	3.4 (0.8)	3.3 (0.8)	3.2 (0.8)	2.9 (0.9)	3.0 (0.9)	2.2 (1.0)	3.2 (0.9)	2.9 (1.0)
trust Fauci	3.0 (1.0)	3.7 (0.6)	3.5 (0.8)	3.4 (0.8)	3.4 (0.9)	2.7 (1.0)	2.7 (1.0)	1.8 (1.0)	3.0 (1.0)	2.8 (1.1)
trust scientists	3.4 (0.8)	3.8 (0.5)	3.6 (0.7)	3.6 (0.7)	3.5 (0.7)	3.3 (0.8)	3.2 (0.8)	2.7 (1.0)	3.4 (0.8)	3.2 (0.8)
trust news media	2.3 (1.0)	2.9 (0.8)	2.7 (0.9)	2.6 (0.9)	2.6 (0.9)	2.1 (1.0)	2.1 (0.9)	1.4 (0.7)	2.4 (1.0)	2.0 (0.9)
trust social media	2.1 (0.9)	2.2 (0.9)	2.1 (0.9)	2.2 (0.9)	2.2 (0.9)	2.0 (0.9)	2.1 (0.9)	1.4 (0.7)	2.3 (1.0)	1.8 (0.8)
follow news										
Not at all	1,218 (4.8%)	2 (0.1%)	7 (1.8%)	44 (1.6%)	7 (0.9%)	100 (3.2%)	280 (9.1%)	47 (9.3%)	95 (1.4%)	635 (10%)
Not very closely	3,336 (13%)	43 (2.3%)	35 (9.2%)	211 (7.5%)	52 (6.6%)	328 (10%)	688 (22%)	99 (19%)	369 (5.6%)	1,510 (24%)

Somewhat closely	11,388 (44%)	541 (28%)	163 (43%)	1,265 (45%)	353 (45%)	1,548 (49%)	1,512 (49%)	210 (41%)	2,758 (41%)	3,037 (48%)
Very closely	9,678 (38%)	1,315 (69%)	178 (46%)	1,284 (46%)	373 (48%)	1,186 (37%)	612 (20%)	152 (30%)	3,424 (52%)	1,155 (18%)

Note: Trust variables are on a 1-4 where higher is more trusting. Statistics show weighted mean and standard deviation (weighted n and % for categorical variables)

Table E7. Trust in Institutions (February 2021)

Variable	Overall, N = 21,502	Multiple (not FB, Fox, Newsmax), N = 1,705	Only Biden Admin., N = 1,264	Only CNN, N = 2,302	Only MSNBC, N = 752	Only Fox, N = 3,030	Only Facebook, N = 3,224	Only Newsmax, N = 500	Multiple (including FB, Fox, Newsmax), N = 4,885	None of these, N = 3,840
trust White House	2.7 (1.0)	3.3 (0.8)	3.0 (0.9)	3.0 (0.9)	3.0 (0.9)	2.4 (1.0)	2.5 (1.0)	1.8 (0.9)	2.8 (1.0)	2.4 (1.0)
trust CDC	3.1 (0.9)	3.5 (0.7)	3.4 (0.8)	3.3 (0.7)	3.3 (0.8)	2.8 (1.0)	2.9 (0.9)	2.0 (1.0)	3.1 (0.9)	2.9 (1.0)
trust Fauci	2.9 (1.0)	3.7 (0.7)	3.4 (0.8)	3.4 (0.8)	3.3 (0.9)	2.6 (1.1)	2.7 (1.0)	1.7 (1.0)	3.0 (1.0)	2.7 (1.1)
trust scientists	3.3 (0.8)	3.7 (0.6)	3.6 (0.7)	3.5 (0.6)	3.5 (0.7)	3.2 (0.8)	3.2 (0.8)	2.7 (1.0)	3.4 (0.7)	3.2 (0.8)
trust news media	2.3 (1.0)	2.8 (0.8)	2.5 (0.9)	2.6 (0.9)	2.6 (0.9)	2.0 (1.0)	2.1 (0.9)	1.4 (0.7)	2.4 (1.0)	2.0 (0.9)
trust social media	2.0 (0.9)	2.1 (0.9)	2.1 (0.9)	2.2 (0.9)	2.1 (0.8)	1.9 (0.9)	2.1 (0.9)	1.4 (0.7)	2.3 (1.0)	1.7 (0.8)
follow news										
Not at all	1,116 (5.2%)	2 (0.1%)	37 (3.0%)	48 (2.1%)	6 (0.8%)	114 (3.8%)	295 (9.2%)	58 (12%)	48 (1.0%)	506 (13%)
Not very closely	3,367 (16%)	44 (2.6%)	133 (11%)	233 (10%)	111 (15%)	453 (15%)	813 (25%)	104 (21%)	334 (6.8%)	1,142 (30%)
Somewhat closely	9,789 (46%)	570 (33%)	628 (50%)	1,038 (45%)	360 (48%)	1,561 (52%)	1,583 (49%)	228 (46%)	2,157 (44%)	1,664 (43%)

Very closely	7,221 (34%)	1,089 (64%)	465 (37%)	981 (43%)	274 (36%)	901 (30%)	532 (16%)	109 (22%)	2,343 (48%)	527 (14%)

Note: Trust variables are on a 1-4 where higher is more trusting. Statistics show weighted mean and standard deviation (weighted n and % for categorical variables)

Table E8. Trust in Institutions (April 2021)

Variable	Overall, N = 21,733	Multiple (not FB, Fox, Newsmax), N = 1,672	Only Biden Admin., N = 1,655	Only CNN, N = 2,331	Only MSNBC, N = 750	Only Fox, N = 2,992	Only Facebook, N = 3,132	Only Newsmax, N = 411	Multiple (including FB, Fox, Newsmax), N = 5,347	None of these, N = 3,443
trust White House	2.8 (1.0)	3.5 (0.7)	3.1 (0.9)	3.1 (0.8)	3.1 (0.9)	2.3 (1.0)	2.5 (1.0)	1.8 (0.9)	2.9 (1.0)	2.5 (1.0)
trust CDC	3.1 (0.9)	3.6 (0.7)	3.4 (0.8)	3.3 (0.8)	3.3 (0.8)	2.8 (1.0)	2.9 (1.0)	2.1 (1.0)	3.2 (0.9)	2.9 (1.0)
trust Fauci	2.9 (1.1)	3.7 (0.6)	3.4 (0.9)	3.3 (0.9)	3.4 (0.9)	2.4 (1.1)	2.6 (1.0)	1.7 (1.0)	3.0 (1.0)	2.7 (1.1)
trust scientists	3.3 (0.8)	3.7 (0.5)	3.5 (0.7)	3.5 (0.7)	3.5 (0.7)	3.2 (0.8)	3.1 (0.8)	2.8 (1.0)	3.4 (0.8)	3.2 (0.8)
trust news media	2.3 (1.0)	2.8 (0.8)	2.5 (0.9)	2.6 (0.9)	2.6 (0.9)	2.1 (1.0)	2.1 (0.9)	1.4 (0.7)	2.5 (1.0)	2.0 (0.9)
trust social media	2.1 (0.9)	2.2 (0.8)	2.1 (0.9)	2.3 (0.9)	2.1 (0.9)	1.9 (0.9)	2.1 (0.9)	1.4 (0.7)	2.4 (1.0)	1.8 (0.8)
follow news										
Not at all	1,195 (5.5%)	7 (0.4%)	37 (2.2%)	78 (3.3%)	8 (1.1%)	127 (4.2%)	259 (8.3%)	67 (16%)	105 (2.0%)	507 (15%)
Not very closely	3,433 (16%)	50 (3.0%)	183 (11%)	226 (9.7%)	98 (13%)	472 (16%)	847 (27%)	105 (26%)	394 (7.4%)	1,058 (31%)
Somewhat closely	9,896 (46%)	593 (35%)	855 (52%)	1,120 (48%)	362 (48%)	1,560 (52%)	1,506 (48%)	182 (44%)	2,255 (42%)	1,464 (43%)
Very closely	7,203 (33%)	1,021 (61%)	580 (35%)	907 (39%)	282 (38%)	832 (28%)	520 (17%)	56 (14%)	2,590 (48%)	414 (12%)

Note: Trust variables are on a 1-4 where higher is more trusting. Statistics show weighted mean and standard deviation (weighted n and % for categorical variables)

Table E9. Trust in Institutions (June-July 2021)

Variable	Overall, N = 20,669	Multiple (not FB, Fox, Newsmax), N = 1,421	Only Biden Admin., N = 1,541	Only CNN, N = 2,258	Only MSNBC, N = 759	Only Fox, N = 2,883	Only Facebook, N = 3,289	Only Newsmax, N = 441	Multiple (including FB, Fox, Newsmax), N = 4,635	None of these, N = 3,442
trust White House	2.7 (1.0)	3.4 (0.7)	3.1 (0.9)	2.9 (0.9)	3.1 (0.9)	2.4 (1.0)	2.5 (1.0)	1.7 (0.9)	2.9 (1.0)	2.4 (1.0)
trust CDC	3.0 (1.0)	3.5 (0.7)	3.3 (0.8)	3.2 (0.9)	3.3 (0.8)	2.7 (1.0)	2.9 (1.0)	2.1 (1.1)	3.1 (0.9)	2.9 (1.0)
trust Fauci	2.8 (1.1)	3.6 (0.7)	3.3 (0.9)	3.2 (0.9)	3.3 (0.9)	2.3 (1.1)	2.5 (1.1)	1.6 (1.0)	2.9 (1.1)	2.6 (1.1)
trust scientists	3.3 (0.8)	3.7 (0.5)	3.5 (0.7)	3.5 (0.7)	3.5 (0.7)	3.1 (0.9)	3.1 (0.9)	2.6 (1.0)	3.3 (0.8)	3.2 (0.8)
trust news media	2.3 (1.0)	2.8 (0.8)	2.5 (0.9)	2.5 (0.9)	2.6 (0.9)	2.1 (1.0)	2.1 (0.9)	1.5 (0.8)	2.5 (1.0)	1.9 (0.9)
trust social media	2.1 (0.9)	2.1 (0.9)	2.0 (0.8)	2.2 (0.9)	2.1 (0.9)	1.9 (0.9)	2.1 (0.9)	1.5 (0.8)	2.4 (1.0)	1.7 (0.8)
follow news										
Not at all	1,424 (6.9%)	12 (0.8%)	42 (2.7%)	108 (4.8%)	20 (2.7%)	212 (7.4%)	382 (12%)	49 (11%)	98 (2.1%)	500 (15%)
Not very closely	3,453 (17%)	46 (3.2%)	185 (12%)	260 (12%)	74 (9.7%)	492 (17%)	887 (27%)	105 (24%)	318 (6.9%)	1,087 (32%)
Somewhat closely	9,271 (45%)	524 (37%)	836 (54%)	1,053 (47%)	392 (52%)	1,373 (48%)	1,511 (46%)	185 (42%)	1,929 (42%)	1,469 (43%)
Very closely	6,515 (32%)	839 (59%)	476 (31%)	836 (37%)	272 (36%)	804 (28%)	510 (16%)	102 (23%)	2,291 (49%)	385 (11%)

Note: Trust variables are on a 1-4 where higher is more trusting. Statistics show weighted mean and standard deviation (weighted n and % for categorical variables)

Institutional Trust: Principal Components Analysis

The tables above show average trust scores by news type for each of six relevant people/institutions. The survey waves in question ask respondents to report their trust in sixteen such people/organizations to "do the right thing to best handle the current coronavirus (COVID-19) outbreak":

- Your city government
- Your state government
- The White House
- Congress
- Donald Trump
- Joe Biden
- The CDC (Centers for Disease Control and Prevention)
- Dr. Anthony Fauci of the National Institute of Health
- The FDA (Food and Drug Administration)
- Hospitals and doctors
- Pharmaceutical companies
- Scientists and researchers
- The police
- Banks
- The news media
- Social media companies

To more systematically interrogate the extent to which respondents in different news types differ in their institutional trust, we reduce the dimensionality of responses to these trust items using principal components analysis – estimating the latent dimensions in the data separately by survey wave.

Scree plots for each wave are shown in Figure E1. In each wave, the eigenvalue for the first two components are greater than one; in only one such wave (the earliest) is the third, and in this case it is marginal (1.02). As such, we extract the first two components for each wave.

Figure E1: Scree Plots by Wave (Institutional Trust)

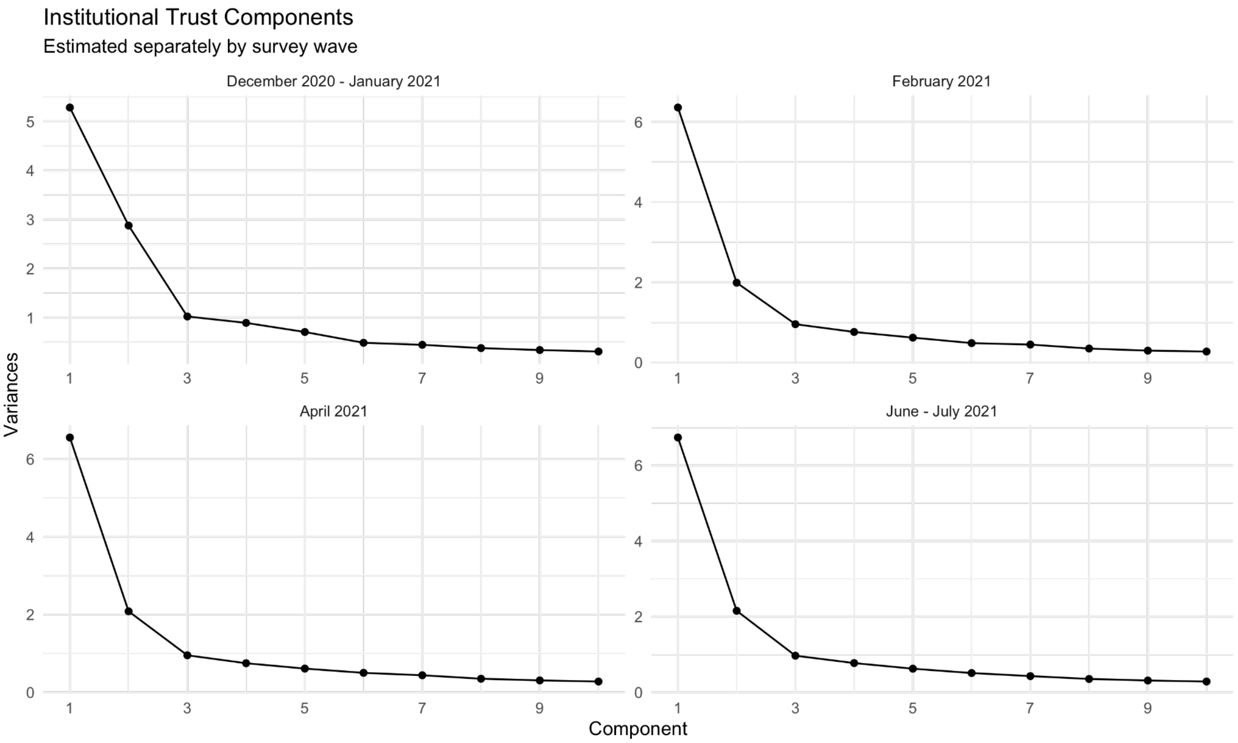

Tables D9 and D10 show the factor loadings for the first and second component in each wave. These components are generally similar in each wave. They are generally consistent across waves, with the exception of variables whose substantive meanings changed following the 2020 general election (such as Donald Trump, the White House, and Congress). In general, higher scores on the first component generally indicate higher trust in institutions overall, including state and local governments, the the FDA and CDC, social media companies, and the news media – as well as left-leaning public figures such as Joe Biden and Dr. Anthony Fauci. By contrast, the second component indicates higher trust in right-leaning individuals/institutions such as the police, banks, and Donald Trump.

Table E10: Institutional Trust First Component Loadings by Wave

	December 2020 - January 2021	February 2021	April 2021	June - July 2021
Your City Government	0.257	0.210	0.215	0.220
Your State Government	0.283	0.234	0.229	0.234
The White House	0.086	0.337	0.343	0.335

Congress	0.230	0.285	0.281	0.273
Donald Trump	-0.084	-0.179	-0.150	-0.121
Joe Biden	0.350	0.370	0.367	0.356
The FDA	0.286	0.262	0.265	0.267
The CDC	0.303	0.290	0.293	0.296
Dr. Anthony Fauci	0.345	0.342	0.348	0.352
Hospitals and Doctors	0.177	0.150	0.146	0.154
Pharmaceutical Companies	0.260	0.215	0.214	0.228
Scientists and Researchers	0.231	0.210	0.202	0.215
The Police	0.156	0.091	0.089	0.105
Banks	0.187	0.133	0.137	0.145
The News Media	0.312	0.287	0.286	0.282
Social Media Companies	0.251	0.216	0.233	0.229

Table E11: Institutional Trust Second Component Loadings by Wave

	December 2020 - January 2021	February 2021	April 2021	June - July 2021

Your City Government	0.119	0.213	0.199	0.184
Your State Government	0.101	0.198	0.196	0.180
The White House	0.508	-0.042	-0.065	-0.062
Congress	0.228	0.066	0.067	0.077
Donald Trump	0.610	0.635	0.621	0.621
Joe Biden	-0.286	-0.263	-0.228	-0.226
The FDA	-0.001	0.055	0.013	-0.005
The CDC	-0.076	-0.040	-0.088	-0.108
Dr. Anthony Fauci	-0.208	-0.185	-0.209	-0.242
Hospitals and Doctors	0.006	0.093	0.067	0.059
Pharmaceutical Companies	0.091	0.194	0.177	0.153
Scientists and Researchers	-0.061	0.018	-0.006	-0.033
The Police	0.306	0.454	0.476	0.471
Banks	0.225	0.367	0.371	0.376
The News Media	-0.067	0.009	0.047	0.059

Social Media Companies	0.046	0.108	0.145	0.148

Finally, we show the average scores for respondents in each news type (unweighted) on each of these first two components by survey wave. These are reproduced in the main manuscript (Figure 9). Some of the average loadings fluctuate between the first (December 2020 - January 2021) wave and the rest, especially with respect to the second component, though as mentioned above this is likely due to the transfer of power following the 2020 election. However, overall there are clear patterns. New types associated with left-leaning sources, such as MSNBC and the Biden administration, tend to have high scores on the first component and low scores on the second. News types associated with right-leaning sources, such as Fox and Newsmax, show the reverse: low scores on the first component, high scores on the second. Finally, the "Only Facebook" and "None of these" types tend to average lower scores on the first dimension but *not* higher scores on the second. They are less trusting of left-leaning and health-related institutions, but they are not particularly trusting in their right-leaning counterparts.

Figure E2: Average Loadings by News Type, First Two Components

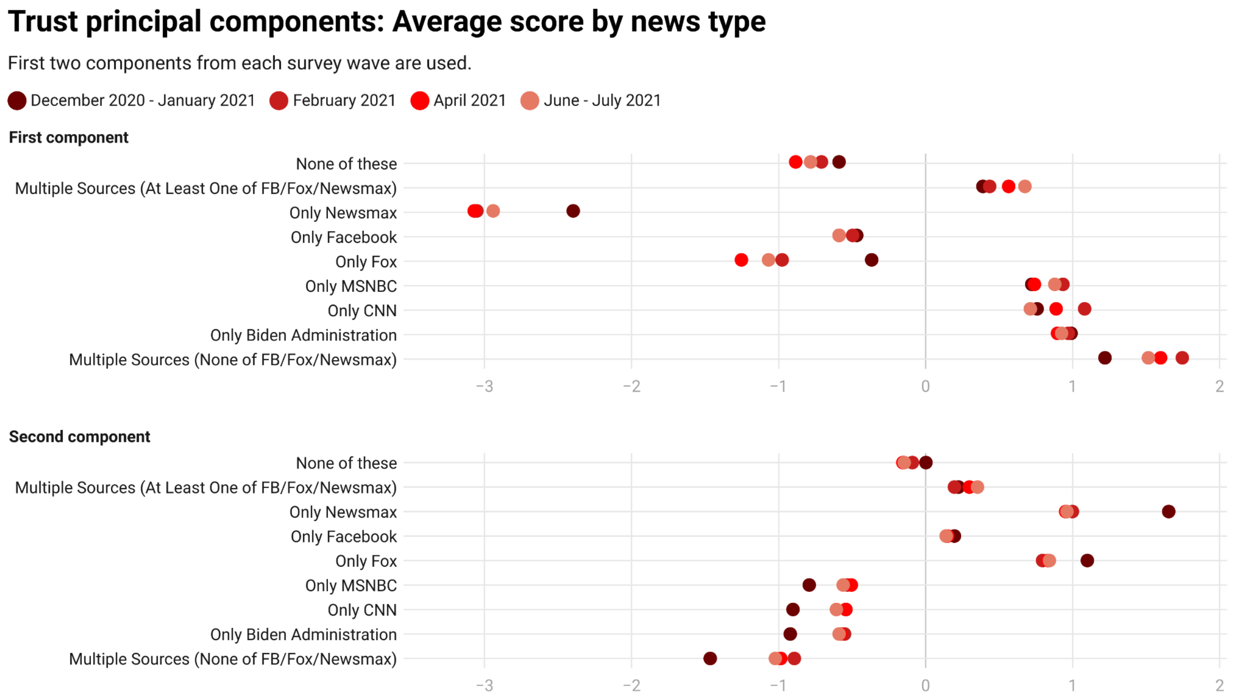

Supplement: pgad146_Supplementary_Data [file pgad146_supplementary_data.zip › PNASNEXUS-PNASNEXUS-2022-00931-s04.pdf]
